# The pipeline for drugs for control and elimination of neglected tropical diseases: 1. Anti-infective drugs for regulatory registration

**DOI:** 10.1186/s13071-022-05581-4

**Published:** 2023-03-01

**Authors:** Kenneth M. Pfarr, Anna K. Krome, Issraa Al-Obaidi, Hannah Batchelor, Michel Vaillant, Achim Hoerauf, Nicholas O. Opoku, Annette C. Kuesel

**Affiliations:** 1 Institute of Medical Microbiology, Immunology and Parasitology, University Hospital Bonn, Bonn, Germany; 2 German Center for Infection Research, Partner Site Bonn-Cologne, Bonn, Germany; 3 Department of Pharmaceutical Technology and Biopharmaceutics, University of Bonn, Bonn, Germany; 4 Strathclyde Institute of Pharmacy and Biomedical Sciences, University of Strathclyde, Glasgow, UK; 5 Competence Center for Methodology and Statistics, Luxembourg Institute of Health, Strassen, Grand Duchy of Luxembourg; 6 Department of Epidemiology and Biostatistics School of Public Health, University of Health and Allied Sciences, Hohoe, Ghana; 7 UNICEF/UNDP/World Bank/WHO Special Programme for Research and Training in Tropical Diseases (WHO/TDR), World Health Organization, Geneva, Switzerland

**Keywords:** Neglected topical diseases, Drug development, Acoziborole, Bedaquiline, Emodepside, Flubentylosin, Fexinidazole, Fosravuconazole, JNJ-64281802, Moxidectin, Oxantel pamoate, Oxfendazole, Implementation research

## Abstract

The World Health Organization ‘Ending the neglect to attain the Sustainable Development Goals: A road map for neglected tropical diseases 2021–2030’ outlines the targets for control and elimination of neglected tropical diseases (NTDs). New drugs are needed to achieve some of them. We are providing an overview of the pipeline for new anti-infective drugs for regulatory registration and steps to effective use for NTD control and elimination. Considering drugs approved for an NTD by at least one stringent regulatory authority: fexinidazole, included in WHO guidelines for *Trypanosoma brucei gambiense* African trypanosomiasis, is in development for Chagas disease. Moxidectin, registered in 2018 for treatment of individuals ≥ 12 years old with onchocerciasis, is undergoing studies to extend the indication to 4–11-year-old children and obtain additional data to inform WHO and endemic countries' decisions on moxidectin inclusion in guidelines and policies. Moxidectin is also being evaluated for other NTDs. Considering drugs in at least Phase 2 clinical development, a submission is being prepared for registration of acoziborole as an oral treatment for first and second stage *T.b. gambiense* African trypanosomiasis. Bedaquiline, registered for tuberculosis, is being evaluated for multibacillary leprosy. Phase 2 studies of emodepside and flubentylosin in *O. volvulus*-infected individuals are ongoing; studies for *Trichuris trichuria* and hookworm are planned. A trial of fosravuconazole in *Madurella mycetomatis*-infected patients is ongoing. JNJ-64281802 is undergoing Phase 2 trials for reducing dengue viral load. Studies are ongoing or planned to evaluate oxantel pamoate for onchocerciasis and soil-transmitted helminths, including *Trichuris*, and oxfendazole for onchocerciasis, *Fasciola hepatica*, *Taenia solium* cysticercosis, *Echinococcus granulosus* and soil-transmitted helminths, including *Trichuris*. Additional steps from first registration to effective use for NTD control and elimination include country registrations, possibly additional studies to inform WHO guidelines and country policies, and implementation research to address barriers to effective use of new drugs. Relative to the number of people suffering from NTDs, the pipeline is small. Close collaboration and exchange of experience among all stakeholders developing drugs for NTDs may increase the probability that the current pipeline will translate into new drugs effectively implemented in affected countries.

**Graphical Abstract:**

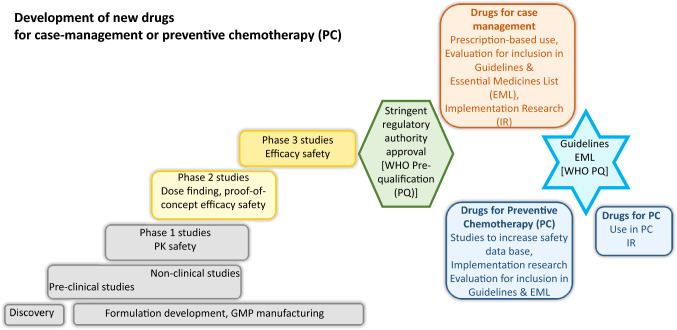

**Supplementary Information:**

The online version contains supplementary material available at 10.1186/s13071-022-05581-4.

## Background

**Fig. 1:**
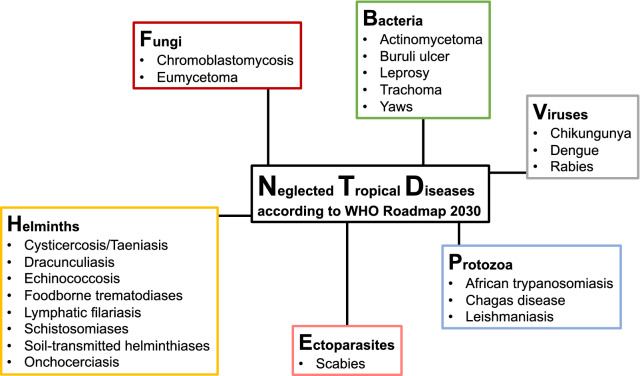
Infectious NTDs included in the Roadmap

Neglected tropical diseases (NTDs) are infectious diseases affecting more than 1 billion people in low- and middle-income countries (LMIC) [1]. The diseases considered NTDs differ slightly between different organizations. We consider here the infectious diseases included in the World Health Organization (WHO) ‘Ending the neglect to attain the Sustainable Development Goals: A road map for neglected tropical diseases 2021–2030’ (subsequently referred to as ‘Roadmap’ [1]) (Fig. 1). The Roadmap was endorsed by the 73rd World Health Assembly (WHA) in 2020 [2].

The Roadmap summarizes current core strategic interventions and invokes or specifies the need for new drugs, optimized drug regimens and/or new formulations to achieve the Roadmap targets for many NTDs [1].

We are providing an overview of new small molecule anti-infective drugs in at least Phase 2 clinical development for regulatory registration for NTDs, summarizing information as available and as we considered it of interest to NTD researchers and public health managers for a particular drug. Vaccines, monoclonal antibodies and drugs to treat signs and symptoms or secondary infections are not covered.

We identified the drugs through search of the WHO International Clinical Trials Registry for studies initiated from 2009 onward. The source registries, with last import date as of the search date 8 October 2021 (Additional File 1 Table S1) and last source registry update by the sponsors/investigators, determined the trials included in the registry at that time and thus the drugs we considered. At the time of writing, the source registry record was accessed for updates. On 3 July 2022, we searched the WHO International Clinical Trials Registry again for any relevant updates (last import date from all source registries 30 or 31 May 2022).

We based our categorization of drugs as in ‘development for regulatory registration’ on information available on study sponsor websites and in publications. In the absence of specific information, we used sponsor track record in bringing drugs to regulatory approval.

As new drugs progress through the stages of clinical development [3], the probability of success, i.e. achieving regulatory registration (also referred to as ‘regulatory approval’ or ‘marketing authorization’), increases. We are therefore separately presenting drugs which have already received regulatory approval in at least one country for at least one NTD indication and those that have not yet received regulatory approval for an NTD in any country.

NTDs are prevalent primarily in LMIC. To ensure, to the extent possible, that readers in LMIC can access the references, all references are open access, accessible as author manuscripts in the US National Institutes of Health’s National Library of Medicine (Pubmed Central, PMC), on the European Molecular Biology Laboratory European Bioinformatics Institute platform (Europe PMC), on institutional websites or accessible via HINARI to the WHO staff co-author (ACK). HINARI is a WHO initiative through which local, not-for-profit institutions in LMIC can obtain free or very low-cost online access to the major journals in biomedical and related social sciences [4]. Currently, institutions in 125 LMIC are eligible for HINARI. Publishers do not provide the same access to the publications in HINARI to all eligible institutions. WHO has a relatively limited level of access and we used WHO staff access as the criterion for ‘accessible via HINARI’. In the absence of publications, the websites of the US Food and Drug Administration (US FDA) and the European Medicines Agency (EMA) as well as trial sponsor websites were searched. For context, the Additional File 1 includes an overview of current core strategic interventions and WHO perspectives on the need for new drugs, combinations or formulations as per the Roadmap (Additional File 1 Table S2) as well as information on the drugs used in these strategies we consider of interest (Additional File 1 "Anti-infective drugs for diseases for which preventive chemotherapy is the main strategic core intervention" and "Anti-infective drugs for diseases for which case management is the main control- and elimination strategy" section).

## Drugs approved for an NTD by at least one stringent regulatory authority

### Fexinidazole

Fexinidazole is a nitroimidazole, which entered preclinical development in the 1970s as a possible broad-spectrum antimicrobial. Investigation of its activity against *Trypanosoma cruzei* and *T. brucei* dates back to the early 1980s. Further research into the anti-trypanosomal activity of fexinidazole [5] and pre-clinical development of fexinidazole as an oral drug candidate for human African trypanosomiasis (HAT) was initiated by the Drugs for Neglected Diseases initiative (DND*i*). The results motivated initiation of Phase 1 pharmacokinetic and safety studies [6] and a legal agreement between DND*i* and Sanofi for manufacturing, regulatory registration and distribution in 2009 [7, 8]. The phase 2/3 study in individuals with stage 1 or early stage 2 *T.b. gambiense* HAT was initiated in 2012 [9]. The results provided the pivotal clinical data supporting a European Medicines Agency (EMA) positive ‘scientific opinion’ through Article 58 of Regulation (EC) No. 726/2004 procedure in the context of cooperation with WHO (now referred to as EU-M4all) in 2018 for treatment of individuals ≥ 6 years and weighing ≥ 20 kg with stage 1 and stage 2 *T.b. gambiense* HAT [10, 11]. For a ‘Scientific Opinion’, the EMA assesses drugs not intended for marketing in the European Union according to the same criteria used for drugs for the European market. Since WHO considers the EMA a ‘stringent regulatory authority’ [12], a positive ‘Scientific Opinion’ facilitates WHO prequalification [13] and registration in endemic countries. In 2019, fexinidazole was added to the WHO Model List of Essential Medicines List (EML) for adults and children [14] and included in the ‘WHO interim guidelines for the treatment of gambiense human African trypanosomiasis’ [15] (for more information, see Additional File 1).

A study evaluating the efficacy and safety of fexinidazole in patients ≥ 6 years old with *T.b. rhodesiense* HAT (Clinical Trials.gov study identifier (CTI): NCT03974178; Pan African Clinical Trials Registry identifier (PACTRI): PACTR202011638938739) is expected to complete primary data collection in June 2022.

DND*i* also initiated clinical development for Chagas disease. A Phase 2 study of fexinidazole in Chagas disease, initiated in 2014 in Bolivia, was discontinued because of safety and tolerability issues with high doses tested for > 14 days. Based on the safety and efficacy data obtained with the lower doses, a second Phase 2 study was initiated in 2022 (CTI: NCT NCT03587766). The data supported the conclusion that low doses have high efficacy with acceptable safety and tolerability and that continuation of development of fexinidazole for Chagas disease is indicated [16].

### Moxidectin

Moxidectin is, like ivermectin, a macrocyclic lactone but is a milbemycin, not an avermectin. Macrocyclic lactones have activity against a broad spectrum of endo- and ecto-parasites. They are agonists of the glutamate-gated chloride channels, present in the neurons and pharyngeal muscles of nematodes and arthropods, but not of humans. Activation of the channels inhibits movement and pharyngeal pumping, leading to paralysis and ultimately death [17–19]. While the major mode of action of both molecules is through binding to glutamate-gated chloride channels, the difference in chemical structure results in different physico-chemical and thus pharmacokinetic and pharmacodynamic properties, which are reflected in different efficacy and safety profiles [19, 20].

Onchocerciasis: The UNICEF/UNDP/World Bank/WHO Special Programme for Research and Training in Tropical Diseases (WHO/TDR) initiated research on moxidectin in the late 1990s, in consultation and with the support of the Onchocerciasis Control Programme in West Africa (OCP) and the African Programme for Onchocerciasis Control (APOC). Clinical development for onchocerciasis and lymphatic filariasis (LF) was supported by the WHO/TDR-funded in vitro and animal pharmacology studies as well as the non-clinical safety studies for veterinary use registration provided by Fort Dodge Animal Health to WHO/TDR for external expert assessment. Clinical development for onchocerciasis was initiated in collaboration with Wyeth Pharmaceuticals. The development plan was informed by ‘scientific advice’ from the EMA in view of obtaining an EMA positive ‘scientific opinion’ through the Article 58 of Regulation (EC) No. 726/2004 procedure in the context of cooperation with WHO (now referred to as EU-M4all). For a ‘Scientific Opinion’, the EMA assesses drugs not intended for marketing in the European Union according to the same criteria used for drugs for the European market. A positive ‘Scientific Opinion’ facilitates WHO prequalification and registration in endemic countries. Following acquisition of Wyeth by Pfizer and Pfizer’s withdrawal from the collaboration agreement WHO and Wyeth had concluded, TDR completed the Phase 3 study on its own (last participant visit May 2012, database lock and unblinding December 2013). In 2014, WHO licensed all moxidectin-related data at its disposal to Medicines Development for Global Health (MDGH), a not-for-profit Australian Health Charity. MDGH raised funding for all activities required for a ‘New Drug Application’ (NDA) to the US FDA by leveraging the prospect of a ‘Priority Review Voucher’ [21]. The US FDA approved an 8 mg moxidectin dose for treatment of onchocerciasis in individuals ≥ 12 years old. US FDA review summaries and the moxidectin label are available [22].

Eight single-dose clinical studies supported the registration. The six pharmacokinetic and safety studies in healthy adult male and female volunteers showed that moxidectin is safe and without clinically significant effect on the QT interval at the highest dose of 36 mg tested, its absorption is not affected in a clinically significant way by administration with or without food, clinically significant drug interactions are unlikely and 0.7% of a single 8 mg maternal dose is excreted into breast milk [23–27]. The Phase 2 and 3 studies in *O. volvulus*-infected individuals in Ghana, Liberia and the Democratic Republic of the Congo (DRC) showed that a single 8 mg dose results in higher and more sustained reduction in skin microfilariae levels than the standard ivermectin dose (150 µg/kg) with a comparable safety profile [28–30].

Modelling using the data from the Phase 2 study suggests that inclusion of moxidectin mass drug administration (MDA) in national onchocerciasis control and elimination policies could accelerate progress towards onchocerciasis elimination [31, 32]. Three ongoing studies are designed to provide additional data for WHO and countries to decide on inclusion of moxidectin in guidelines and policies for onchocerciasis control and elimination: A study to increase the safety database in individuals with and without measurable *O. volvulus* skin microfilariae levels (CTI: NCT04311671) and a study to compare the safety and efficacy of three annual and five biannual doses of 8 mg moxidectin or 150 µg/kg ivermectin (CTI: NCT03876262) are ongoing in DRC. A study to identify a dose for 4–11-year-old children (CTI: NCT03962062) is being conducted in Ghana using the US FDA approved 2 mg tablet, whose size was chosen in view of potential use down to the age of 4 years. The studies are co-funded by MDGH, the Luxembourg National Research Fund and an EDCTP grant [33]. The repeat dose study will provide the first data on the relative effect of repeat dosing with moxidectin or ivermectin on the reproductive capacity of *O. volvulus*, i.e. skin repopulation with microfilariae, the *O. volvulus* life stage which is transmitted. Repeat dosing with ivermectin has a cumulative effect on macrofilariae reproductive capacity [34, 35]. For the impact on parasite transmission, the mechanism through which skin repopulation with microfilariae is reduced or eliminated (permanent sterilization of macrofilariae, reduction of their reproductive life span or reduction of their life span/cidal effect) is irrelevant.

In an in vitro model in which worms were incubated for 6 days, moxidectin, like ivermectin, inhibited *O. volvulus* L3 moulting and L4 motility but with lower IC50 and IC90 absolutely and relative to the maximum serum concentrations and the half-life of moxidectin and ivermectin. Thus, moxidectin could have a ‘prophylactic effect’ against new infections [36]. Should moxidectin have such an effect in humans, this effect could reduce the negative impact of ‘non-compliers’ (those who do not take the drug) on elimination goals by reducing new infections due to transmission of skin microfilariae from the non-compliers.

The MDGH pipeline for moxidectin includes scabies, lymphatic filariasis, soil-transmitted helminths and strongyloidiases (in clinical development) and head lice (in preclinical development).

Scabies: Pre-clinical studies suggest that moxidectin could advance scabies control and clinical trials have been identified as a priority [37–41]. A Phase 2 dose finding study in Australia, Austria and France was completed in February 2022 (CTI: NCT03905265). A study planning to compare a single topically administered dose of moxidectin (cydectin, an animal formulation) and three daily topically administered doses of ivermectin (iverzine) in Egypt was registered in 2019 in the Iranian Registry of Clinical Trials (identifier: IRCT20191030045275N). The record has not been updated since February 2020 and shows that the study is not yet recruiting.

LF: A study in adults comparing the effect of three annual administrations of 200 µg/kg ivermectin or 8 mg moxidectin in combination with 400 mg albendazole or 400 mg albendazole plus 6 mg/kg diethylcarbamazine on *Wuchereria bancrofti* microfilaremia was initiated in Côte d’Ivoire in August 2020 (CTI: NCT04410406).

Soil-transmitted helminthiases (STH) including strongyloidiasis: The Phase 3 study in *O. volvulus*-infected individuals was not designed to compare efficacy of 8 mg moxidectin and 150 µg/kg ivermectin on STH. The data collected supported the hypothesis that moxidectin has better efficacy against hookworms. There were too few individuals with *Ascaris* and *Trichuris* infection at baseline to allow conclusions (supplemental information to [30]).

The Swiss Tropical and Public Health Institute (STPH) conducted studies evaluating the efficacy of a single moxidectin dose using the veterinary formulation cydectin [42] or tablets manufactured at the University of Basel, Switzerland [42–45], alone or in combination with other anthelminthics against *Strongyloides stercoralis*, *Trichuris trichiura* and concomitant other helminths and against schistosomiasis (see Additional File 1 Table S3 for tabulation of results). All studies found that moxidectin was well tolerated.

*Strongyloides stercoralis*: The cure rate (CR) of a single 8 mg moxidectin dose was inferior to that of a single 200 µg/kg ivermectin dose [42]. The dose response showed similar CR for 4, 8 and 12 mg moxidectin [44]. A population pharmacokinetic model supports further exploration of fixed rather than weight-based dosing [46]. A Phase 3 study comparing 8 mg moxidectin to 200 µg/kg ivermectin is planned for 2022 (CTI: NCT04848688). Pharmacometric modelling suggests that a two-dose regimen (e.g. two 8 mg moxidectin doses, 3 weeks apart) should be evaluated to achieve high CR in individuals with moderate to high infection intensity [47].

*Trichuris trichiura*: A single 8 mg moxidectin dose co-administered with 400 mg albendazole was highly efficacious [43]. A dose ranging study confirmed this, and showed that 16 mg or 24 mg moxidectin ﻿do not increase efficacy as monotherapy or in combination with albendazole [45]. Between-study efficacy differences might be due to differences in pre-study egg counts. A study in adolescents and adults comparing 8 mg moxidectin + 400 mg albendazole, 400 mg albendazole and 200 µg/kg ivermectin + 400 mg albendazole [48] was completed in October 2021 (CTI: NCT04726969).

Hookworm: CRs after 8 mg moxidectin or 200 µg/kg ivermectin were moderate [42]. Combination of 8 mg moxidectin with tribendimidine or albendazole improved CR [43]. Increasing the moxidectin dose to 16 mg or 24 mg did not increase egg reduction rates (ERR) or CR for either monotherapy or albendazole combination treatment [45].

*Ascaris lumbricoides*: CRs after 8 mg moxidectin were > 95% [43, 45].

Foodborne trematodiasis: Neither moxidectin nor ivermectin was effective against *Opisthorchis viverrini* [42].

Schistosomiasis: The results to date suggest that potential treatment programmes with 8 mg moxidectin for other infections will have no co-benefit for schistosomiasis control [49].

Paediatric formulation. The size of the approved 2 mg tablet (8.13 mm × 4.55 mm × 2.50 mm average thickness) was chosen to accommodate children ≥ 4 years of age. To facilitate dosing of younger children and in view of the moxidectin development programme for scabies, development of a paediatric formulation is ongoing, co-funded by MDGH, the Luxembourg National Research Fund (FNR no. INTER/EDCTP/19/14338294/MiniMox/Vaillant) and an EDCTP grant (RIA2019PD-2880, MiniMox-Treatment for all: a paediatric formulation of moxidectin for neglected infectious diseases).

## Drugs in at least Phase 2 clinical development for first regulatory registration for an NTD

### Acoziborole

Acoziborole (SCYX-7158, AN5568) is a benzoxaborole. Discovery of novel benzoxaboroles with anti-trypanosomal activity [50, 51] resulted in a lead-optimization programme. Acoziborole emerged from this as a compound with an in vitro and in vivo absorption, distribution, metabolism, elimination (ADME), toxicology and efficacy profile that met the criteria for initiation of preclinical studies intended to qualify acoziborole for clinical development for stage 2 human African trypanosomiasis (HAT) [52, 53]. Research into the mode of action currently points to the RNA cleavage and polyadenylation specificity factor submit 3 (CPSF3) as a target of benzoxaboroles in trypanosomes (as also in *Plasmodium* spp. and *Toxoplasma gondii*) reducing the amount of mRNA and stopping protein synthesis [54, 55].

A Phase 1 study in France in healthy volunteers of sub-Saharan origin showed a half-life of 17 days and no issues preventing further clinical development [55]. The Phase 2/3 study of the efficacy and safety of a single oral 960 mg dose in 208 patients with first- or second-stage *Trypanosoma brucei gambiense* HAT to 18 months post-treatment (CTI: NCT03087955) was analysed in 2021. The data to date, including those of a Phase 1 study to improve the understanding of acoziborole ADME (CTI: NCT04270981) sponsored by DND*i*, will be complemented by non-clinical studies required by the US FDA and the EMA for registration of new chemical entities [56]. A study of the safety and tolerability of acoziborole in *T.b. gambiense* HAT seropositive individuals without parasitological confirmation of infection (CTI: NCT05256017) was initiated by DND*i* in 2021.

Sanofi will be the regulatory sponsor and has committed to manufacturing and providing acoziborole free of charge to HAT-endemic countries within its collaboration with WHO [57], now extended to 2025 [58].

### Bedaquiline

Bedaquiline (TMC207, JNJ-16175328-AAA, R207910) is a diarylchinoline which inhibits mycobacterial ATP synthase, a mode of action different from that of other anti-mycobacterial drugs [59]. Bedaquiline was approved as part of combination treatment of pulmonary multi-drug-resistant tuberculosis in adults by the US FDA in 2012 [60] and in adults and adolescents by the EMA in 2014 [61]. In 2019, the US FDA approval was extended to 12–17-year-old adolescents [62] and has been included in WHO guidelines on treatment of drug-resistant tuberculosis [63, 64].

Based on data from murine models of leprosy [59], Janssen Research & Development is developing bedaquiline for *Mycobacterium leprae* infections. An open-label study is ongoing in Brazil to evaluate the efficacy and safety of bedaquiline monotherapy in individuals with multibacillary leprosy (CTI: NCT03384641).

### Emodepside

Emodepside is an N-methylated cyclic octadepsipeptide authorized in 2005 by the EMA as a spot-on solution combination product with praziquantel for treatment of dogs and cats with or at risk of infection with nematodes (*Toxocara cati, Toxascaris leonina, Ancylostoma tubaeforme*) and cestodes (*Dipylidium caninum, Taenia taeniaeformis, Echinococcus multilocularis*) (sponsor Bayer HealthCare AG) [65]. Since then, modified release tablets have been authorized as well as a combination product with toltrazirul [66].

Cyclic octadepsipeptides have a novel mechanism of action that was recently reviewed together with the majority of pre-clinical pharmacology studies supporting clinical studies for onchocerciasis as well as LF [67, 68].

Emodepside emerged as a drug candidate for human use from the WHO/TDR drug discovery programme [69, 70]. The last step funded by WHO/TDR was an ex vivo study by Drs K Awadzi, S. Tagboto and S. Townson at the Onchocerciasis Chemotherapy Research Center in Ghana in 2006–2007. It examined the effect of emodepside, provided by Bayer, on motility and viability of male and female *O. volvulus* macrofilariae obtained via nodulectomy. The data (unpublished, report from Dr Townson to TDR available from the corresponding author) together with the pharmacology and safety data from the veterinary registration identified emodepside not only as a candidate for onchocerciasis but also for soil-transmitted helminths [65].

#### Onchocerciasis

In 2014, DNDi and Bayer agreed to develop emodepside. Two safety and tolerability studies in healthy male volunteers showed that single and multiple doses of a liquid formulation and single doses of immediate release tablets (5 or 20 mg emodepside) are well tolerated. Emodepside was rapidly absorbed after administration of the liquid in the fasted state and somewhat slower in the fed state [71]. A Phase 2 safety, tolerability, pharmacodynamics, pharmacokinetics and efficacy study in *O. volvulus*-infected individuals in Ghana has started recruitment (PACTRI: PACTR202010898529928). The study is sponsored by DNDi and conducted by researchers at the University of Health and Allied Sciences, Ghana.

In an in vitro model in which worms were exposed to emodepside for 6 days, emodepside inhibited *O. volvulus* L3 moulting and L4 motility with both IC_50_ and IC_90_ around a factor 100 lower than those for moxidectin. Considering the pharmacokinetic profile of emodepside in humans available to date, emodepside might have a prophylactic effect against new infection [36].

#### Trichuris trichiura and hookworm

In 2021, the STPH sponsored a study in Tanzania evaluating efficacy and safety of single doses of 5 mg to 30 mg (CTI: NCT05017194). STPH has obtained a European Research Council grant (grant agreement ID: 101019223, https://doi.org/10.3030/101019223), which includes funding for two dose-selection phase 2a trials and a phase 2b trial comparing efficacy and safety of the selected dose with that of albendazole.

### Flubentylosin (TylAMac, ABBV-4083)

Flubentylosin (TylAMac, ABBV-4083) is a derivative of tylosin A, an antibiotic of the macrolide class approved for veterinary use since the 1990s. Research into the role of the endosymbiotic *Wolbachia* bacteria for filarial development and how antibiotics affect filariae fertility and viability was initiated in the 1990s [72–76]. Studies evaluating the effect of 6 weeks of daily doxycycline treatment provided clinical proof of concept of the macrofilariae sterilizing and cidal effect of anti-*Wolbachia* treatment for onchocerciasis and LF [77–80]. Doxycycline-induced *Wolbachia* depletion killed up to 70% of *O. volvulus* adult female worms within 2 years and rendered the remaining worms sterile [81, 82]. Drugs with a virtually 100% macrofilariae sterilizing or macrofilaricidal effect after a single treatment course could significantly accelerate elimination of *O. volvulus* transmission [83] compared to ivermectin, which has only a partial macrofilaricidal and modest sterilizing effect after at least four annual treatments [35].

Anti-*Wolbachia* drugs will not be effective against *Loa loa*, which does not contain *Wolbachia* [84, 85]. The efficacy of ivermectin against *L. loa* can lead to serious adverse reactions in individuals with high *L. loa* microfilariaemia [86, 87]. In areas which are at least onchocerciasis meso-endemic, this element of ivermectin’s safety profile requires that MDA including ivermectin is implemented with special provisions to ensure prompt identification and management of serious adverse reactions [88]. In other *L. loa* co-endemic areas, alternative strategies for control and elimination of onchocerciasis and lymphatic filariasis are required [89].

The clinical proof of concept spurred further studies with commercially available antibiotics as well as discovery programmes for antibiotics with potentially shorter treatment regimens, no contraindication in children and ideally safe in pregnant women [90–93].

Flubentylosin has emerged from discovery programmes at the Anti-*Wolbachia* Consortium funded by the Bill and Melinda Gates Foundation since 2007 [90, 91, 94] and the pharmaceutical company AbbVie Inc. (North Chicago, IL, USA). Studies in *Litomosoides sigmodontis* mice and Mongolian gerbil models showing that flubentylosin reduced *Wolbachia* levels by ≥ 99.9% [90] encouraged progression into development. The results of Phase 1 studies conducted by AbbVie justified preparation of a Phase 2 study. The Phase 2 study investigating the safety, efficacy and pharmacokinetics of 7 or 14 days of dosing with TylAMac alone or in combination with albendazole, ivermectin or albendazole plus ivermectin started recruitment in 2021 (PACTRI: PACTR202104600961505, CTI: NCT04913610). The study is sponsored by AbbVie and conducted in collaboration between DNDi and researchers in DRC.

### Fosravuconazole

Fosravuconazole l-lysine ethanolate (Fosravuconazole, E1224) is a prodrug of ravuconazole. It is an orally available broad-spectrum antifungal triazole, developed by Esai Co., Japan, since 2007. Since 2018, fosravuconazole containing medication has been available in Japan for treatment of onychomycosis [95, 96].

The in vitro minimum inhibitory concentrations of ravuconazole against *Madurella mycetomatis*, the most common causative species of eumycetoma, were substantially lower than those of ketoconazole [97]. Esai and DNDi concluded a collaboration and license agreement for development of fosravuconazole for Chagas disease in 2009 and for eumycetoma in 2015 [95].

The primary target of ravuconazole in *Trypanosoma cruzi* is the cytochrome P450-dependent sterol C14α demethylase resulting in inhibition of ergosterol biosynthesis, a mechanism of action common to the azoles [98–100].

Fosravuconazole treatment of adults with chronic indeterminate Chagas disease induced sustained parasitological clearance to month 12 in 13/45 (29%) of participants treated with the highest dose (4000 mg over 8 weeks) compared to 37/45 (82%) of participants treated with benznidazole (5 mg/kg/day for 60 days) [101]. These findings resulted in discontinuation of development of fosravuconazole as monotherapy for Chagas disease and initiation of a trial in Bolivia in adults with chronic indeterminate Chagas disease comparing benznidazole regimens with and without fosravuconazole. The rates of sustained parasitological clearance 6 and 12 months after the end of treatment suggested that shorter or lower dose benznidazole treatment has efficacy comparable to that of the standard benznidazole regimen with a better safety profile. Fosravuconazole addition did not increase clearance rates [102]. With only 27 to 30 participants per treatment arm, additional data are needed to confirm the efficacy and safety of shorter or lower dose and thus safer/better tolerable benznidazole regimens [7, 102].

In vitro, minimum inhibitory concentrations against *M. mycetomatis* isolates were lower than those of ketoconazole, itraconazole [97] and other azoles [95] and ravuconazole also showed activity in an invertebrate in vivo model [103]. The in vitro results in combination with fosravuconazole’s half-life of 7–11 days and safety profile motivated initiation of a trial in eumycetoma at the Mycetoma Research Centre in Sudan in 2017 [95, 97, 104]. The trial (CTI: NCT03086226) compares the efficacy of weekly treatment with 200 or 300 mg fosravuconazole in *M. mycetomatis*-infected patients to that of daily treatment with 400 mg itraconazole. Follow-up of all participants has been completed and database lock planned for March 2022 [105].

### JNJ-64281802

Janssen Research & Development, LLC, is conducting three Phase 2 trials to assess the ability of its compound JNJ-64281802 to reduce dengue viral load and to assess its safety (CTI: NCT04480736, NCT05048875, NCT04906980). A study to assess safety and efficacy for the prevention of dengue infection is in preparation (CTI: NCT05201794).

Neither HINARI nor PubMed search for publications including JNJ-64281802 in any field identified any publications. A March 2022 search of the Janssen website and the website on the Johnson&Johnson pharmaceutical pipeline for investors also did not identify any information.

### LXE408

LXE408 emerged from optimization of GNF6702, a selective inhibitor of the kinetoplastid proteasome. While GNF6702 showed excellent efficacy in murine models of leishmaniasis, as well as of Chagas disease and human African trypanosomiasis, formulation of GNF6702 for oral availability would have resulted in an expensive drug. In *Leishmania donovani*-infected mice, oral doses of 1 mg/kg LXE408 twice per day for 8 days resulted in a 95% liver parasite reduction, the reduction achieved with oral doses of 12 mg/kg miltefosine four times a day. Data from murine models suggest that LXE408 may also be a potential drug candidate for cutaneous leishmaniasis [106, 107].

A Phase 1 multiple ascending dose study of LXE408 was completed in September 2021 and a Phase 2 study in patients with visceral leishmaniasis is planned (Clinical Trials Registry-India identifier: CTRI/2022/03/040775). The Drugs for Neglected Diseases initiative (DND*i*) concluded a collaboration and license agreement in early 2022 with Novartis to develop LXE408 as a potential oral treatment for visceral leishmaniasis. The development is financially supported by Wellcome [108]**.**

### Oxantel Pamoate

Oxantel pamoate is a tetrahydropyrimidine derivative and *m*-oxyphenol analogue of pyrantel discovered in the 1970s. Oxantel activates parasite nicotinic acetylcholine receptors (AchR), leading to overstimulation of the muscles, muscle spasms and paralysis [109–111]. An AchR subtype recently discovered in the pig whipworm *Trichuris suis* was named O-AchR because of its sensitivity to oxantel [109]. The responsible O-AchR subunit was also identified in *T. muris* [112]. This receptor may explain oxantel efficacy against *Trichuris* spp.

Oral formulations of oxantel pamoate in combination with pyrantel pamoate and praziquantel have been marketed for companion animals for many years. Oral formulations in combination with pyrantel pamoate are approved for human use in six South American countries and the Philippines, including for children in the Philippines, Ecuador and Peru [111].

Clinical studies demonstrating the efficacy of oxantel pamoate against *T. trichiura* date back to the 1970s and were recently reviewed together with the available non-clinical data [111]. The data support development of oxantel pamoate for approval by a stringent regulatory authority. The Helminth Elimination Platform (HELP) consortium, formed in 2019 to develop drugs for STH and onchocerciasis, is pursuing this with European Union funding (European Union’s Horizon 2020 research and innovation programme grant agreement no. 815628). Studies should include not only oxantel mono-treatment arms but also two or three drug combination treatment arms [113, 114] to inform both regulatory approval and WHO guidelines and country policies.

### Oxfendazole

Oxfendazole is a benzimidazole veterinary anthelmintic approved in the US [115] and in Europe for treatment of roundworms and tapeworms in ruminants and horses and giardiasis in dogs [116].

The ‘Oxfendazole Development Group’ (ODG, [117]), which obtained non-profit status in the US in 2016, is pursuing oxfendazole registration for treatment of *Fasciola hepatica*, *Taenia solium* cysticercosis, *Echinococcus granulosus* and STH, in particular *Trichuris*. The US FDA granted Fast-track designation for trichuriasis recognizing its public health importance and the unmet medical need [118, 119].

An important question for oxfendazole development for tissue-dwelling parasites is whether the required systemic exposure has unacceptable, benzimidazole class effect, toxicity. Such toxicity resulted in discontinuation of flubendazole development for onchocerciasis [120–123]. Non-clinical pharmacology, pharmacokinetic and toxicology data to date were recently reviewed and support development for both gut and tissue dwelling parasites [116, 124].

A single and a multiple ascending dose Phase 1 study with a veterinary oral liquid formulation in healthy volunteers [125, 126] identified no safety concerns. The pharmacokinetic data showed a significant food effect as well as significant nonlinear pharmacokinetics with less than dose-proportional exposure attributed to absorption because of low oxfendazole solubility. A single and multiple dose Phase 1 trial (CTI: NCT04920292) of a tablet formulation is expected to complete primary outcome measure collection in September 2022.

An ODG and Asociacion Benefica Prisma sponsored Phase 2 trial (CTI: NCT03435718; Clinical Trials Peruvian Registry identifier PER-083–20) comparing single 6 mg/kg, 15 mg/kg or 30 mg/kg oxfendazole doses, three daily (15 mg/kg) oxfendazole doses and a single 400 mg albendazole dose in *T. trichiura* infection is planned to start recruitment in July 2022 in Peru. An Asociacion Benefica Prisma sponsored Phase 2 trial (CTI: NCT04713787) in Peru plans to compare single 400 mg or 800 mg oxfendazole doses with a single 400 mg albendazole dose against *T. trichiura*.

Treatment of the rodent filaria *L. sigmodontis* for 3–5 days showed macrofilaricidal without microfilaricidal activity [127], suggesting oxfendazole could be a macrofilaricide for control and elimination of onchocerciasis (and potentially LF) safe in loiasis co-endemic areas because of the lack of microfilaricidal activity. An STPH-sponsored Phase 1 trial in Tanzania (CTI: NCT04920292,) was initiated in May 2022 to examine pharmacokinetics, safety and tolerability of single and multiple doses of a new tablet formulation of oxfendazole. Should the dose of oxfendazole needed for anti-onchocercal activity prove well tolerated, the EU-funded HELP consortium will conduct a Phase 2 trial comparing oxfendazole with ivermectin in onchocerciasis. In addition, clinical trials are being designed to investigate the efficacy and safety of oxfendazole for STH.

In an in vitro assay exposing *O. volvulus* larvae for 6 days to oxfendazole, the IC_50_ and IC_90_ for inhibition of L3 moulting were higher than those of emodepside but lower than those of moxidectin and ivermectin. In conjunction with available pharmacokinetic data, it was concluded that oxfendazole could have a prophylactic effect against new infections [36].

Close collaboration between the ODG and the HELP consortium could make development of oxfendazole more cost-effective and address potential safety concerns, e.g. whether undiagnosed neurocysticercosis could result in adverse reactions to oxfendazole in participants of trials for onchocerciasis or STH.

## From first registration to effective use for NTD control and elimination

### Regulatory approval

Not all drugs described above may ultimately obtain regulatory approval. Based on historical data, out of 2–5 drugs entering Phase 2 development on average only 1–2 enter Phase 3 and out of those on average one will achieve regulatory approval [3].

Drugs for NTDs which are not also a health problem in high income countries, i.e. are of economic interest, face the additional hurdle of the willingness of pharmaceutical company partners to continue the development/collaboration and/or the willingness or ability of donors to continue financial support to complete all activities required for a submission for regulatory approval. As described above, a case in point for this hurdle is moxidectin development, which was put in jeopardy, and regulatory registration delayed by several years, because Pfizer withdrew from the collaboration agreement with WHO [29, 30].

Prior to use in any LMIC, the regulatory authority of that country needs to approve the use of the drug in their country. The dossier that sponsors have to submit for regulatory approval has to include all information from the required non-clinical studies, clinical studies and pharmaceutical development and qualification of the drug substance and drug product. An overview of the elements of a dossier is available in guideline M4 of the ‘Common Technical Document’ of the ‘International Council for Harmonisation of Technical Requirements for Pharmaceuticals for Human Use’ (ICH). A dossier consequently includes hundreds of thousands of pages and demands extensive regulatory authority staff and time capacity. For example, the New Drug Application submitted by MDGH to the US FDA for registration of moxidectin included more than 400,000 pages and at least 28 US FDA staff were involved in the review as deduced from the publicly available 525 pages of US FDA review summaries [128]. In recognition of the fact that the required review capacity is not available in all LMIC, WHO has established the ‘Collaborative Procedure for Accelerated Registration’ of Medicines that have been prequalified by WHO or approved by a ‘stringent regulatory authority’ (SRA) [12]). A SRA is defined as a regulatory authority which is a member of the ICH, being: the European Commission, the US Food and Drug Administration and the Ministry of Health, Labour and Welfare of Japan, also represented by the Pharmaceuticals and Medical Devices Agency (as before 23 October 2015); or is an ICH observer, being the European Free Trade Association, as represented by Swissmedic, and Health Canada (as before 23 October 2015); or is a regulatory authority associated with an ICH member through a legally binding, mutual recognition agreement, including Australia, Iceland, Liechtenstein and Norway (as before 23 October 2015) [12]. The list of stringent regulatory authorities and the list of countries participating in the Collaborative Procedure for SRA approved drugs are available on the WHO website.

### Inclusion in WHO guidelines and country policies

Notwithstanding the increasing requirement for pricing agreements between pharmaceutical companies and health care payers [129, 130], for drugs that will be marketed by a company for prescription by physicians, regulatory approval (marketing authorization) can be regarded as the ‘final milestone’. This is not necessarily the case for drugs that will be used by the public health systems of LMICs. For such drugs, inclusion into WHO guidelines is frequently a prerequisite for inclusion in country policies. Inclusion in WHO guidelines may require evidence beyond that needed for regulatory registration [131].

Additional data are, in particular, needed for drugs for disease control and elimination via ‘Preventive Chemotherapy’ (PC), i.e. administration of the drug to specified populations irrespective of the presence of symptoms or infection, such as occurs for lymphatic filariasis, onchocerciasis, soil-transmitted helminths, taeniasis and cysticercosis, schistosomiasis, foodborne trematodiases, yaws, trachoma or scabies (Additional File 1 Table S2) [1, 132, 133]. During PC many of the people taking the drug will not be infected and will consequently not derive a direct benefit from taking the drug. This means that any adverse reactions to the drug have to be minimal to ensure the risk is acceptable relative to the indirect benefit that the uninfected individuals will derive from PC. The indirect benefit is the reduced probability of becoming infected via the impact on parasite transmission due to the treatment of infected community members. The number of uninfected individuals included in studies conducted for regulatory registration, typically not exceeding a few hundred healthy volunteers in Phase 1 studies, is insufficient to support decisions on use of drugs for PC, and consequently additional studies need to be conducted. During PC, the availability of trained health care staff to assess and, as required, treat any adverse reactions is significantly lower than when physicians prescribe a drug for case management of an individual patient. Consequently, the safety database for infected individuals required to recommend use of a new drug for PC also needs to be larger than required for regulatory approval of a drug for case management. Thus, infected individuals also need to be included in additional studies to ensure data are available on the type and frequency of rare adverse reactions in a study population representative of that which will be included in PC.

For drugs to be used for case management (see Additional File 1 Table S2 for NTDs whose control and/or elimination strategy is case management based), the need for additional data to inform WHO guidelines may not be as extensive since the population included in clinical studies for regulatory approval is more representative of the population that will be treated by the health systems. Furthermore, patients will be treated and followed up by health care professionals similar to how they were treated and followed up during the clinical studies. For example, the inclusion of fexinidazole in the 2019 ‘WHO interim guidelines for the treatment of gambiense human African trypanosomiasis’ was based exclusively on the clinical studies submitted to the EMA for registration [15].

Regulatory agencies such as the US FDA and the EMA have established processes through which sponsors can obtain advice on drug/indication specific data the agencies will expect and plan the relevant studies accordingly. In contrast, WHO guidelines are usually developed ‘post-hoc’, i.e. based on systematic review and evaluation of publicly available data, initiated when ‘Member States, WHO country offices, external experts or other stakeholders ask for guidance on a clinical or public health problem or policy area’. It is at the discretion of the members of the ‘Guideline Development Group,’ established by WHO for a specific guideline under consideration, to decide whether the data available are those they consider necessary to support a new guideline [131]. This approach may reflect the fact that, in the past, few drugs have been developed specifically to address health problems that do not provide the prospect of return/profit from investment, i.e. for diseases in LMIC, the countries that are typically requesting WHO guidelines to inform their policies [134–136]. Exceptions were the development of ivermectin for onchocerciasis and triclabendazole for human fascioliasis (see Additional File 1). Both development programmes occurred when WHO guideline development was less formalized. For sponsors and funders of drugs developed for NTDs and in particular for PC, the current approach to WHO guideline development constitutes a big problem: resources and time into studies to complement the data acquired for regulatory registration are invested based on consultation with experts and possibly WHO NTD staff. Their views on the type and amount of data required to inform guidelines may be different from the views of the Guideline Development Group. WHO has started to address this problem by piloting a ‘WHO Coordinated Scientific Advice procedure’ [137].

### Implementation research

Effective translation of WHO Guidelines and country policies into practice may require implementation research (IR, also referred to as ‘operational research’), defined here as research to identify, understand and address barriers to effective and quality implementation of health interventions, strategies and policies in a particular setting [131].

Depending on the differences between the drug currently used for PC and the new drug, in particular with respect to treatment regimen, safety profile and eligible population, IR may be needed on how best to implement PC with the new drug and to gather information on the acceptability of the new treatment and how it is provided at the individual, community and health system level. Beyond data on the adverse reactions that will impact acceptability by the population (defined here as the percentage of the eligible population willing to take the drug each time it is provided and the willingness of the communities to engage in drug distribution, if applicable), such data cannot be obtained during clinical studies for registration or post-registration studies to gather additional safety and efficacy data since those are typically double-blinded studies. Even if not double-blinded, such studies include many elements that are not part of distribution during PC (or case management) and will impact participants’ perceptions of the drug. Such elements include informed consent/assent, pre- and post-treatment examinations and compensation for time lost from gainful activities due to study participation.

‘Acceptability’ of a new drug to the individuals/communities to be treated and other stakeholders, such as local health care workers, national programmes/Ministries of Health, collaborating non-governmental organisations and funders as well as effective integration of the new intervention into local practice depend on many factors. These include not only the benefit/risk ratio of the drug but also the specific economic, health system and political and social-cultural context in which the new intervention will be implemented. Locally adapted IR studies may also be needed to address implementation challenges. IR may also be needed to provide further evidence to inform guidelines and country policies. Ideally such research would be conducted based on a common ‘core protocol’ to ensure data can be analysed across studies/countries and should be implemented in collaboration between different national programmes and researchers to ensure timely sharing of lessons learnt. Such an approach has been initiated to address research needs identified in the ‘WHO consolidated guidelines on drug-resistant tuberculosis treatment’ [138–140].

Based on the certainty of evidence that informed a guideline and on the knowledge gaps identified in the systematic reviews and by the Guideline Development Group, WHO guidelines specify research needs [131].

## Conclusions

Between 1975–1999 and between 2000–2011, 13 and five drugs, respectively, were approved by the US FDA and/or the EMA for an NTD [141, 142]. Even assuming our criteria for categorization drugs as in ‘development for regulatory registration’ were too strict, the number of drugs in at least Phase 2 clinical development and the 2018 registration of fexinidazole for treatment of first and second stage *T.b. gambiense* HAT and moxidectin for onchocerciasis show that investment into drug development continues to be disproportionate to the number of people affected by NTDs. Another sad ‘continuation’ is that development of drugs for NTDs depends heavily on public funding (government, intergovernmental organization) or funding from private not-for-profit organizations.

Entry of new actors such as the HELP consortium and the Oxfendazole Development Group is encouraging. Development for regulatory approval requires specific experience and expertise to ensure all elements of a submission meet regulatory requirements for registration and to ensure that the post-registration obligations can be met (e.g. regular updates on safety data acquired in any context and anywhere in the world). Such experience, expertise and systems are concentrated in pharmaceutical companies. Lack of such expertise was identified as a significant obstacle in a 2019 workshop ‘Repurposing of off-patent drugs: Research and Regulatory Challenges’, hosted by the US National Institute for Health National Center for Advancing Translational Sciences (NCATS) and co-sponsored by the NCATS Cures Acceleration Network Review Board, NCATS Drug Development Partnership Programs, US FDA and the Reagan-Udall Foundation for the US FDA [143]. We hope that relevant expertise is available to, or will be sought by, the HELP consortium and the Oxfendazole Development Group. An ‘NTD drug developer working group’ joining all organisations developing drugs for registration for NTDs might be helpful to share lessons learnt. Accessing such expertise as well as synergistic, and thus cost-effective, investments into development of new drugs for NTDs would benefit from a central resource on: (1) all for-profit and not-for-profit organisations working on pre-clinical and clinical development of drugs for NTDs and/or funding such development, (2) the expertise they have available and might be willing to contribute to projects in other organisations, (3) the drugs in pre-clinical and clinical development (see e.g. website on drugs in different stages of development for cystic fibrosis, https://apps.cff.org/trials/pipeline), (4) the funding available and funding gaps and (5) investigators with experience in conducting clinical trials in LMIC. Some of this information is currently available on the WHO Global Observatory on Health R&D (https://www.who.int/observatories/global-observatory-on-health-research-and-development). Funders of capacity strengthening on health research are collaborating in the ‘Essence for Health Research’ initiative (https://tdr.who.int/groups/essence-on-health-research/about-us).

The short period between the first ‘registration’ (EMA ‘positive scientific opinion’) of fexinidazole and registration in *T.b. gambiense* HAT-endemic countries, inclusion in the WHO EML, WHO EML for Children [64] and WHO interim guidelines [15] has been attributed to continuing interactions among all stakeholders, including DNDi, Sanofi, the EMA and WHO [8]. Equivalent interactions had been in place for development of moxidectin. These interactions were ‘disrupted’ by the withdrawal of Pfizer from the collaboration agreement with WHO as well as by the dissolution of APOC. The ‘Expanded Special Project for Elimination of Neglected Tropical Diseases’ in WHO/AFRO (ESPEN, [144]), while sometimes perceived as the APOC ‘successor’, differs substantially from APOC in terms of its mandate, resources and operating model as does the WHO/NTD department. The moxidectin development plan to registration had been generated based on a series of meetings with the EMA for scientific advice in view of submission for an EMA ‘scientific opinion’. MDGH chose submission to the US FDA rather than the EMA because they could leverage the possibility of a priority review voucher for the funds they needed [21]. APOC’s ‘Technical Consultative Committee’ [21] had provided input into the clinical development plan as well as the initial plans for studies to acquire additional data needed to inform guidelines. Through APOC, country NTD/onchocerciasis programmes and the non-governmental organizations supporting onchocerciasis control programmes in the endemic countries were kept up to date. While the moxidectin example shows that ‘the best laid plans’ may be compromised by extraneous events, the collaborations ‘disrupted’ for moxidectin but successfully concluded for fexinidazole appear to be the approach of choice for development of new drugs for NTDs to registration, WHO guidelines, EML inclusion, country policies and practice. The ‘WHO Coordinated Scientific Advice procedure’ during its pilot phase and beyond will streamline sponsors receiving feedback from different WHO departments.

## Supplementary Information

**Additional file 1: Table S1.** Source trial registries and last import date into the WHO International Clinical Trials Registry Platform as of 8 October 2021. **Table S2.** Anti-infective drugs, core strategic interventions and gaps in anti-infective drugs as per Roadmap. **Table S3.** Cure and egg reduction rates in phase 2 studies evaluating moxidectin efficacy against *Strongyloides stercoralis, Trichuris trichiura *and concomitant helminths and against *Schistosomahaematobium *and S. mansoni. Section 1 Anti-infective drugs for diseases for which preventive chemotherapy is the main strategic core intervention strategy, Section 2 Anti-infective drugs for diseases for which case management is the main control- and elimination strategy.

## Data Availability

Not applicable.

## Abbreviations

ADME: Absorption, distribution, metabolism, elimination
APOC: African Programme for Onchocerciasis Research
CR: Cure rate
CTI: Clinical Trials.gov Identifier
DNDi: Drugs for neglected diseases initiative
EDCTP: European & Developing Countries Clinical Trials Partnership
EMA: European Medicines Agency
EML: WHO Essential Medicines List
ERR: Egg reduction rate
HAT: Human African trypanosomiasis
ICH: International Council for Harmonisation of Technical Requirements for Pharmaceuticals for Human Use
LMIC: Low- and middle-income countries
IR: Implementation research
LF: Lymphatic filariasis
MDGH: Medicines Development for Global Health
NDA: New drug application
NECT: Nifurtimox-eflornithine combination treatment
NTD: Neglected tropical disease
OCP: Onchocerciasis Control Programme in West Africa
ODG: Oxfendazole development group
PACTRI: Pan African clinical trials registry identifier
PC: Preventive chemotherapy
SRA: Stringent regulatory authority
STH: Soil-transmitted helminths
STPH: Swiss Tropical and Public Health Institute
TDR: UNICEF/UNDP/World Bank/WHO Special Programme for Research and Training in Tropical Diseases
US FDA: United States of America Food and Drug Administration
WHO: World Health Organization
