# Supplementary material for: The pipeline for drugs for control and elimination of neglected tropical diseases: 1. Anti-infective drugs for regulatory registration
Source: Parasit Vectors. 2023 Mar 1;16:82. doi: 10.1186/s13071-022-05581-4 (PMC9979492; doi:10.1186/s13071-022-05581-4)
Supplement: Supplementary file 1 — Additional file 1: Table S1. Source trial registries and last import date into the WHO International Clinical Trials Registry Platform as of 8 October 2021. Table S2. Anti-infective drugs, core strategic interventions and gaps in anti-infective drugs as per Roadmap. Table S3. Cure and egg reduction rates in phase 2 studies evaluating moxidectin efficacy against Strongyloides stercoralis, Trichuris trichiura and concomitant helminths and against Schistosomahaematobium and S. mansoni. Section 1 Anti-infective drugs for diseases for which preventive chemotherapy is the main strategic core intervention strategy, Section 2 Anti-infective drugs for diseases for which case management is the main control- and elimination strategy. [file 13071_2022_5581_MOESM1_ESM.pdf]

Additional File 1

to

The pipeline for drugs for control and elimination of Neglected Tropical Diseases: 1. Anti-infective drugs for regulatory registration

Kenneth M. Pfarr[1†], Anna K. Krome[2†], Issraa Al-Obaidi[3†], Hannah Batchelor[3†], Michel Vaillant[4], Achim Hoerauf[1], Nicholas O. Opoku[5] and Annette C. Kuesel[6*]

† Kenneth M. Pfarr, Anna K. Krome, Issraa Al-Obaidi, Hannah Batchelor contributed equally to this work

[1] Institute of Medical Microbiology, Immunology and Parasitology, University Hospital Bonn, Bonn, Germany, German Center for Infection Research, Partner Site Bonn-Cologne, Bonn, Germany

[2] Department of Pharmaceutical Technology and Biopharmaceutics, University of Bonn, Bonn, Germany

[3] Strathclyde Institute of Pharmacy and Biomedical Sciences, University of Strathclyde, UK

[4] Competence Center for Methodology and Statistics, Luxembourg Institute of Health, Strassen, Grand Duchy of Luxembourg

[5] Department of Epidemiology and Biostatistics School of Public Health, University of Health and Allied Sciences, Hohoe, Ghana

[6] UNICEF/UNDP/World Bank/WHO Special Programme for Research and Training in Tropical Diseases (WHO/TDR), World Health Organization, Geneva, Switzerland.

* Correspondence: kuesela@who.int

Table of Contents

Tables 3

Table S1: Source trial registries and last import date into the WHO International Clinical Trials Registry Platform as of 8 October 2021 3

Table S2: Anti-infective drugs, core strategic interventions and gaps in anti-infective drugs as per Roadmap 4

Table S3: Cure and egg reduction rates in Phase 2 studies evaluating moxidectin efficacy against *Strongyloides stercoralis, Trichuris trichiura* and concomitant helminths; and against *Schistosoma haematobium* and *S. mansoni* 21

1 Anti-infective drugs for diseases for which preventive chemotherapy is the main strategic core intervention strategy 23

1.1 Ivermectin 24

1.2 Albendazole, mebendazole and triclabendazole 24

1.3 Diethylcarbamazine 25

1.4 Praziquantel 25

1.5 Azithromycin 26

1.6 Benzathine penicillin 26

1.7 Topical scabicides 26

1.7.1 Permethrin ... 26
1.7.2 Benzyl benzoate ... 27
1.7.3 Malathion ... 27
1.7.4 Sulphur ointment ... 27
2 Anti-infective drugs for diseases for which case management is the main control- and elimination strategy ... 28
2.1 Antibiotics ... 28
2.1.1 Rifampicin ... 28
2.1.2 Dapsone ... 28
2.1.3 Clofazimine ... 28
2.1.4 Clarithromycin ... 29
2.1.5 Moxifloxacin ... 29
2.1.6 Tetracycline ... 29
2.2 Anti-trypanosomal drugs ... 29
2.2.1 Fexinidazole ... 29
2.2.2 Pentamidine ... 30
2.2.3 Suramin ... 30
2.2.4 Melarsoprol ... 31
2.2.5 Eflornithine ... 31
2.2.6 Benznidazole ... 32
2.2.7 Nifurtimox ... 32
2.3 Antileishmanial drugs ... 32
2.3.1 Pentavalent antimoniate ... 32
2.3.2 Amphotericin B and liposomal amphotericin B ... 33
2.3.3 Miltefosine ... 33
2.3.4 Paromomycin ... 34
2.4 Antifungal drugs ... 34
2.4.1 Itraconazole ... 34
3 References ... 34

Tables

Table S1: Source trial registries and last import date into the WHO International Clinical Trials Registry Platform as of 8 October 2021

Australian New Zealand Clinical Trials Registry	5 July 2021
Chinese Clinical Trial Registry	5 July 2021
ClinicalTrials.gov	5 July 2021
EU Clinical Trials Register (EU-CTR)	5 July 2021
ISRCTN	5 July 2021
The Netherlands National Trial Register	5 July 2021
Brazilian Clinical Trials Registry (ReBec)	24 June 2021
Clinical Trials Registry - India	24 May 2021,
Clinical Research Information Service - Republic of Korea	24 June 2021
Cuban Public Registry of Clinical Trials	21 June 2021
German Clinical Trials Register	21 June 2021
Iranian Registry of Clinical Trials	22 June 2021
Japan Primary Registries Network	15 June 2021
Pan African Clinical Trial Registry	21 June 2021
Sri Lanka Clinical Trials Registry	21 June 2021
Thai Clinical Trials Registry (TCTR)	21 June 2021
Peruvian Clinical Trials Registry (REPEC)	28 June 2021
Lebanese Clinical Trials Registry (LBCTR)	21 June 2021

Table S2: Anti-infective drugs, core strategic interventions and gaps in anti-infective drugs as per Roadmap

NTD, species (WHO Fact Sheet link)	Anti-infective drugs as per 2030 Roadmap [1]	Anti-infective drug-based core strategic interventions [1]<br>Selected complementary core interventions [1]	Gaps in anti-infective drugs as per 2030 roadmap [1]
**NTDs targeted for eradication**			
**Dracunculiasis (Guinea worm)**<br>· *Dracunculus medinensis*<br>https://www.who.int/health-topics/dracunculiasis	None	· NA<br>**Selected core interventions**<br>**WASH**<br>· Village-based active surveillance for rapid case detection and containment<br>· Communication for behavioral impact<br>· Vector control<br>· Tethering of dogs to prevent contamination of the environment [1, 2]	Drugs to treat cases.
**Yaws**<br>· *Treponema pallidum pertenue*<br>https://www.who.int/news-room/fact-sheets/detail/yaws	**Azithromycin**<br>EML: oral liquid 200 mg per 5 mL; 250 mg, and 500 mg oral solid capsule (anhydrous).<br>**Benzathine benzylpenicillin**<br>EML: IM 900 mg in vial benzylpenicillin powder for injection (= 1.2 million IU); 1.44 g in vial benzylpenicillin powder for injection (= 2.4 million IU), indications do not include Yaws [1]	**Preventive chemotherapy**<br>· Initial Total community treatment (TCT): single oral azithromycin (30 mg/kg to maximum of 2g) or<br>· Total targeted treatment (TTT, all active clinical cases and contacts during repeat surveys or in response to localized outbreak): single oral azithromycin (30 mg/kg), liquid formulation for <6 year old children may be available to replace crushing tablets and mixing with water.<br><br>Eligible population: ≥6 months, including pregnant and breastfeeding women.<br>IM Benzathine penicillin (0.6 m units for < 10 years, 1.2 m units for ≥10 years) for people who cannot be treated with azithromycin, fail azithromycin or when azithromycin is not available<br>**Treatment**<br>· Azithromycin (single oral dose) at 30 mg/kg (maximum 2 g) is the preferred treatment.<br>· Benzathine penicillin (single intramuscular dose) at 0.6 million units (children aged under 10 years) and 1.2 million units (people aged over 10 years) can be used for patients with suspected clinical treatment failure after azithromycin, or patients who cannot be treated with azithromycin.<br>[3, 4, 5]	

NTD, species (WHO Fact Sheet link)	Anti-infective drugs as per 2030 Roadmap [1]	Anti-infective drug-based core strategic interventions [1]<br>Selected complementary core interventions [1]	Gaps in anti-infective drugs as per 2030 roadmap [1]
**NTDs targeted for elimination (interruption of transmission)**			
**Human African trypanosomiasis**<br>· *Trypanosoma brucei gambiense*<br>https://www.who.int/health-topics/human-african-trypanosomiasis	**Fexinidazole**<br>EML: 600 mg oral solid.<br>**Eflornithine**<br>EML: IV: 100 mg per mL in 100 mL bottle (hydrochloride).<br>**Nifurtimox**<br>EML: 120 mg tablet<br>**Pentamidine**<br>EML: IM, 200 mg (as isethionate) powder for injection.	**Case management**<br>**Patients aged ≥ 6 years and body weight ≥ 20 kg AND in first-(haemo-lymphatic) stage or non-severe second-(neurological) stage (WBC in CSF < 100/µL)**<br>· Oral fexinidazole, 10 days, weight category dependent dosing<br>**Patients in severe second-stage (WBC in CSF ≥ 100/µL)**<br>· NECT: Oral nifurtimox 5mg/kg TID 10 days, eflornithine 400 mg/kg 7 days<br>**Children aged < 6 years or body weight < 20 kg**<br>· 1st stage disease: IM pentamidine, 4 mg/kg<br>· 2nd stage disease: NECT<br>**Pregnant and lactating women**<br>· 2nd, 3rd trimester: fexinidazole or pentamidine<br>· If clinical condition does not allow to delay treatment, fexinidazole, eflornithine monotherapy or NECT, [6]<br>**Selected complementary core interventions [1]**<br>· WASH<br>· Vector control<br>· Treatment of animals (cattle, pigs)	Safe and efficient single oral dose for both stages (e.g. acoziborole) to help integration of treatment into primary health system<br>Oral formulation for age group <6 years
**Leprosy**<br>· *Mycobacterium leprae*<br>https://www.who.int/health-topics/leprosy	**Rifampicin**<br>EML: 150 mg and 300 mg oral solid.<br>**Dapsone**<br>EML: 25 mg, 50 mg, and 100 mg tablet.<br>**Clofazimine**<br>EML: 50 mg and 100 mg oral solid.	**Case management**<br>**Treatment**<br>· Multibacillary: 12 months treatment, rifampicin, dapsone, clofazimine (doses age dependent) [7]<br>· Paucibacillary: 6 months treatment, rifampicin, dapsone (doses age dependent) [7]<br>**Treatment of drug-resistant leprosy**<br>· Rifampicin resistance: at least two of the following second-line drugs: clarithromycin, minocycline or a quinolone (ofloxacin, levofloxacin or moxifloxacin), plus clofazimine daily for 6 months, followed by clofazimine plus one of the second-line drugs daily for an additional 18 months [7]	New effective medicine or combinations

NTD, species (WHO Fact Sheet link)	Anti-infective drugs as per 2030 Roadmap [1]	Anti-infective drug-based core strategic interventions [1]<br>Selected complementary core interventions [1]	Gaps in anti-infective drugs as per 2030 roadmap [1]
		· Rifampicin and ofloxacin resistance: clarithromycin, minocycline and clofazimine for 6 months followed by clarithromycin or minocycline plus clofazimine for an additional 18 months [7]<br>**Chemoprophylaxis (contacts of leprosy patients, ≥ 2 years upon consent of index case to disclose the disease and adequate management of contacts)**<br>· Single dose rifampicin [7]	
**Onchocerciasis**<br>· *Onchocerca volvulus*<br>https://www.who.int/health-topics/onchocerciasis	**Ivermectin**<br>EML: 3mg tablet (scored).	**Preventive chemotherapy**<br>Depending on pre-control endemicity in combination with (1) co-endemicity of loiasis due to risk of adverse reactions to ivermectin in individuals very high *Loa loa* microfilaraemia and (2) co-endemicity with lymphatic filariasis (LF, see recommendations for LF below)<br>**Onchocerciasis meso- or hyperendemic areas (prevalence of onchocercal nodules among men >20%)**<br>**Areas without loiasis:**<br>· Once to twice yearly single dose of ivermectin [1, 8, 9].<br>**Areas with loiasis co-endemicity:**<br>· *Loa loa* microfilaraemia prevalence <20% or RAPLOA prevalence <40% [8, 10]<br>· *Loa loa* microfilaraemia prevalence ≥20% or RAPLOA prevalence ≥40%: Ivermectin MDA after preparation as per [8] and adaptation of treatment strategy depending on number and treatment coverage of prior ivermectin MDA rounds and occurrence of serious adverse reactions.<br>**Onchocerciasis hypoendemic areas (with or without loiasis):**<br>· Individual, clinic-based ivermectin treatment [8, 11]<br><br>WHO is preparing for new guidance for onchocerciasis elimination strategies for hypoendemic areas where loiasis is co-endemic [1] considering the recommendations of its advisory committees [1, 12, 13].<br>**Dose:** 150µg/kg<br>**Eligible population excludes:** < 90 cm, too sick to be treated (i.e. present for treatment), women having given birth in the last week, pregnant women (note those	Macrofilaricide.<br>Efficacy and safety of moxidectin in children and community settings.<br>Safe drugs safe in *Loa loa* co-endemic areas

NTD, species (WHO Fact Sheet link)	Anti-infective drugs as per 2030 Roadmap [1]	Anti-infective drug-based core strategic interventions [1]<br>Selected complementary core interventions [1]	Gaps in anti-infective drugs as per 2030 roadmap [1]
		excluded should be treated as soon as the exclusion criterion is not met anymore) [11, 14]	
**NTDs targeted for elimination as a public health problem**			
**Chagas disease**<br>· *Trypanosoma cruzi*<br>https://www.who.int/health-topics/chagas-disease	**Benznidazole**<br>EML: 12.5 mg tablet, 50 mg tablet (scored), and 100 mg tablet.<br>**Nifurtimox**<br>EML: 30 mg tablet, 120 mg tablet, and 250 mg tablet.	**Case management**<br>· Benznidazole or nifurtimox treatment to cure infection during acute or early chronic phase, prevent or curb disease progression, in cases of congenital infection<br>· Acute cases: 60 day treatment with<br>· first option - Benznidazole 7.5-10 mg/kg/day for ≤40 kg, 5-7 mg/kg/day for >40 kg in 2-3 fractional daily doses<br>· second option – Nifurtimox 10-15 mg/kg/day for ≤40 kg, 8-10 mf/kg/day for >40 kg in 2-3 fractional doses/day<br>· Congenital cases: 60 day treatment with<br>· First option – Benznidazole 10 mg/kg/ day in 2 to 3 fractional daily doses<br>· Second option – Nifurtimox 10-15 mg/kg in 2 to 3 fractional daily doses<br>· Recent chronic infection<br>· Benznidazole 7.5 mg/kg/day for ≤40 kg, 5 mg/kg/day for >40 kg in 2-3 fractional daily doses<br>Benznidazole ineligible cases: pregnant women, people with kidney or liver failure, chronic cases with specific organ damage<br>Nifurtimox ineligible cases: pregnant women, people with kidney or liver failure, background of neurological or psychiatric disorders, chronic cases with specific organ damage [1, 15, 16]<br><br>**Selected complementary core interventions [1]**<br>· Treatment of disease manifestations<br>· Treatment of women of childbearing potential to prevent congenital transmission<br>· WASH<br>· Vector control<br>· Blood screening	Dosage and duration of benznidazole and nifurtimox treatment, combination treatment, new drugs

NTD, species (WHO Fact Sheet link)	Anti-infective drugs as per 2030 Roadmap [1]	Anti-infective drug-based core strategic interventions [1] Selected complementary core interventions [1]	Gaps in anti-infective drugs as per 2030 roadmap [1]
**Human African trypanosomiasis**<br>· *T. brucei rhodesiense*<br>https://www.who.int/health-topics/human-african-trypanosomiasis	**Suramin sodium**<br>EML: IV, 1 g in vial.<br>**Melarsoprol**<br>EML: IV, 3.6% in 5 mL in ampoule as solution (180 mg active compound).	**Case management**<br>· First stage: Suramin (test dose 4-5 mg/kg day 1, 20 mg/kg every 7 days for 5 weeks (maximum dose/injection 1g)<br>· Second stage: Melarsoprol 2.2 mg/kg per day for 10 days<br>[17]<br>**Selected complementary core interventions** [1]<br>· WASH<br>· Vector control<br>· Treatment of animals (cattle, pigs)	Safe, efficient treatments (e.g., fexinidazole, acoziborole) to replace toxic arsenic-based melarsoprol.
**Visceral leishmaniasis (VL)**<br>· *Leishmania donovani*<br>· *L. infantum*<br>https://www.who.int/health-topics/leishmaniasis	**Pentavalent antimonials**<br>EML: Meglumin antimoniate, IM, 30% in 5 mL ampoule (81 mg/ml), equivalent to about 8.1% antimonate,<br>Sodium stibogluconate, IM-IV, 30-ml vial of 100 mg/ml<br>**Liposomal amphotericin B**<br>EML: IV, 50 mg powder for injection in vial as liposomal complex.<br>**Paromomycin**<br>EML: IM, 750 mg paromomycin as sulfate.<br>**Miltefosine**<br>EML: 10 mg and 50 mg tablet	The treatment of leishmaniasis depends on various factors, e.g., disease, concomitant pathologies, parasite species, and location [1].<br>**Case management for visceral leishmaniasis (VL) based on national guidelines [1]**<br>· *L. infantum*: pentavalent antimonials, liposomal amphotericin B<br>· *L. donovani*: pentavalent antimonials, liposomal amphotericin B, paromomycin, miltefosine<br>For details and ranking of treatment options by causative species and region by the WHO Expert Committee on the Control of Leishmaniases, 2010 see [17]<br>· HIV co-infected patients in patients in East Africa and South-East Asia: liposomal amphotericin B + miltefosine [18, 19, 20]<br>WHO is working on expanding guidance [1]<br>**Selected complementary core interventions** [1]<br>· Vector control<br>· Early diagnosis and treatment	New, safe, cheap oral drugs not requiring cold chain.<br>Shorter first line regimens in East Africa.<br>More treatment options including combination treatments to mitigate risk of resistance.
**Trachoma**<br>· *Chlamydia trachomatis*<br>https://www.who.int/health-topics/trachoma	**Azithromycin**<br>EML: 1.5% Eye drops (solution); oral liquid 200 mg per 5 mL; 250 mg, and	**Preventive chemotherapy**<br>**Evaluation units in which the prevalence of trachomatous inflammation—follicular (TF) among 1–9-year-old children is ≥5%:**	-

NTD, species (WHO Fact Sheet link)	Anti-infective drugs as per 2030 Roadmap [1]	Anti-infective drug-based core strategic interventions [1]<br>Selected complementary core interventions [1]	Gaps in anti-infective drugs as per 2030 roadmap [1]
	500 mg oral solid capsule (anhydrous)<br>**Tetracycline**<br>EML: 1% (hydrochloride) eye ointment.	· Annual MDA of oral azithromycin (20 mg/kg) offered to all aged ≥6 months, until TF prevalence drops to <5%.<br>· If azithromycin is unavailable or contraindicated: tetracycline eye ointment BID to both eyes for 6 weeks.<br>**Selected complementary core interventions** [1]<br>· Surgery for trachomatous trichiasis<br>· Facial cleanliness<br>· Environmental improvement, specifically improvements in access to water and sanitation<br>[21, 22, 23, 24]	
**Lymphatic filariasis**<br>· *Wuchereria bancrofti*<br>· *Brugia malayi*<br>· *Brugia timori*<br>https://www.who.int/health-topics/lymphatic-filariasis	**Ivermectin**<br>EML: 3mg tablet (scored)<br>**Diethylcarbamazine**<br>EML: 50mg and 100 mg tablet.<br>**Albendazole**<br>EML: 400 mg tablet (chewable)	**Preventive chemotherapy**<br>Depending on (1) co-endemicity of loiasis and onchocerciasis due to risk of adverse reactions to ivermectin and diethylcarbamazine and (2) status of MDA programme.<br>**Countries without onchocerciasis or loiasis:**<br>· Annual diethylcarbamazine (6 mg/kg) with 400 mg albendazole (DA)<br>· Annual Ivermectin, diethylcarbamazine plus albendazole (IDA) in areas with less than four two drug treatment rounds; not having met stopping criteria with DA despite having met coverage targets; with infections suggesting local transmission post MDA or post-validation. [25]<br>**Eligible population** excludes pregnant women, children under 2 years of age, and the severely ill. [11]<br>**Countries with onchocerciasis (due to risks of ocular adverse reactions to diethylcarbamazine in *O. volvulus* infected individuals, for review (Awadzi et al., 2015)) in areas without loiasis co-endemicity**<br>· Annual ivermectin (150-200 ug/kg) with 400 mg albendazole (IA), except in areas where biannual ivermectin treatment is being delivered for onchocerciasis [25]<br>**Eligible population** excludes pregnant women, children <90 cm (≈15 kg), severely ill individuals [11]<br>**Countries with onchocerciasis where loiasis is co-endemic (due to risks of severe adverse reactions to ivermectin in individuals with high *Loa loa* microfilaraemia,** [26, 27]**)**	Macrofilaricide, drug safe in *Loa loa* infected individuals

NTD, species (WHO Fact Sheet link)	Anti-infective drugs as per 2030 Roadmap [1]	Anti-infective drug-based core strategic interventions [1]<br>Selected complementary core interventions [1]	Gaps in anti-infective drugs as per 2030 roadmap [1]
		· Biannual albendazole (400 mg) in loiasis co-endemic areas where ivermectin has not already been distributed for onchocerciasis or LF [28]<br>**Eligible population** excludes children in the 1st year of life; pregnant women in the 1st trimester of pregnancy. [11]<br>**Selected complementary core interventions [1]**<br>Vector management in appropriate settings (in particular where *Loa loa* co-endemicity prohibits ivermectin use [28]	
**Schistosomiasis**<br>**Intestinal**<br>· *S. japonicum*<br>· *S. mansoni*<br>· *S. mekongi*<br>**Urinary**<br>· *S. haematobium*<br>https://www.who.int/health-topics/schistosomiasis	**Praziquantel**<br>EML: 600 mg tablet.	**Preventive chemotherapy**<br>Endemic communities with ≥10% *Schistosoma* spp infection prevalence:<br>· Annual treatment with a single dose of all ≥ 2 years of age targeting ≥75% treatment coverage<br>· Consideration should be given to twice yearly preventive chemotherapy in areas with demonstrated lack of appropriate response to annual preventive chemotherapy or high endemicity areas with baseline prevalence ≥50% in school-age children<br><br>Endemic communities with <10% *Schistosoma* spp infection prevalence with ongoing preventive chemotherapy:<br>· Continuation with same or reduced frequency towards interruption of transmission<br>**Test and treat approach**<br>In endemic communities with <10% *Schistosoma* spp infection<br>**Dose**: 40 mg/kg, based on height pole for people ≥94 cm or ≥ 4 years<br>**Eligible population:** all ≥2 years, including pregnant women after the first trimester and lactating women [11, 29]	Improved praziquantel and pediatric formulation.<br>New drugs to complement praziquantel in case of resistance.
**Soil-transmitted helminthiases including strongyloidiasis**<br>· *Ascaris lumbricoides*<br>· Hookworms<br>o *Ancylostoma duodenale*	**Albendazole**<br>EML: 400 mg tablet (chewable).<br>**Mebendazole**<br>EML: 100 mg tablet (chewable),	**Preventive chemotherapy**<br>***A. lumbricoides, T. trichiura, Ancylostoma duodenale, Necator americanus***<br>· Annual single-dose albendazole or mebendazole where baseline prevalence of any soil-transmitted infection is 20% - 50%<br>· Biannual single-dose albendazole or mebendazole where baseline prevalence of any soil-transmitted infection is >50%<br>**Dose**: albendazole 200 mg for 12-23 months, else 400 mg, mebendazole 500 mg	More effective medicines and drug combinations against *T. trichiura* and hookworm infections<br>Drugs and drug combinations to be used

NTD, species (WHO Fact Sheet link)	Anti-infective drugs as per 2030 Roadmap [1]	Anti-infective drug-based core strategic interventions [1]<br>Selected complementary core interventions [1]	Gaps in anti-infective drugs as per 2030 roadmap [1]
o *Necator americanus*<br>· *Trichuris trichiura*<br>· *Strongyloides stercoralis*<br>https://www.who.int/health-topics/soil-transmitted-helminthiases	and 500 mg tablet (chewable).<br>**Ivermectin**<br>EML: 3mg tablet (scored).	**Eligible population**: all young (12-23 months), preschool (24-59 months), school-age children, non-pregnant adolescent girls and women of reproductive age, pregnant women in 2nd and 3rd trimester, lactating women [11, 30]<br>· WHO is preparing guidelines for addition of ivermectin where prevalence of *S. stercoralis* exceeds 10% and in areas with high prevalence of *T. trichiura* [1]<br>**Case management**<br>· Treatment of individuals living in areas endemic for STH and *S. stercoralis* [1]<br>**Selected complementary core interventions [1]**<br>· WASH	in case of emergence of drug resistance
**Rabies**<br>· *Rabies lyssavirus*<br>https://www.who.int/health-topics/rabies	None.	**Case management of confirmed or suspected cases of human rabies**<br>· Thorough wound washing<br>· Post-exposure prophylaxis (PEP) with the rabies vaccine immediately after exposure to a potentially rabid animal<br>· Rabies immunoglobulin for category III exposures immediately after exposure to a potentially rabid animal<br>· Palliative care<br>**Selected complementary core interventions [1]**<br>· Vaccination of dogs and dog population management<br>· Vaccination of people at high risk of exposure to the rabies virus, e.g., laboratory staff working with the rabies virus, veterinarians and animal handlers [1, 31, 32]	Monoclonal antibodies.<br>Anti-virals and agents promoting entry of drugs, antibodies and immune effectors cells across the blood-brain barrier (World Health Organization, 2018g)
**NTDs targeted for control**			
**Cutaneous Leishmaniasis (CL)**<br>· *Leishmania donovani*<br>· *L. infantum*<br>· *L. tropica*<br>· *L. major*<br>· *L. aethiopica*<br>· *L. mexicana*<br>· *L. amazonensis*	**Pentavalent antimoniate**<br>EML: Meglumin antimoniate, IM, 30% in 5 mL ampoule, equivalent to about 8.1% antimonate; Sodium stibogluconate, IM-IV, 30-ml vial of 100 mg/ml<br>**Liposomal amphotericin B**	The treatment of leishmaniasis depends on various factors, e.g., disease, concomitant pathologies, parasite species, location and national guidelines [1]:<br>**Case management**<br>· Topical/intralesional treatment: pentavalent antimonials, paromomycin/methylbenzethonium chloride, cryotherapy, thermotherapy.<br>· Systemic treatment: fluconazole, ketoconazole, liposomal amphotericin B, amphotericin B deoxycholate, pentamidine, pentavalent antimonials (with or without allopurinol), paromomycine, miltefosine.	CL: oral/topical treatment suitable for health center and community level use.

NTD, species (WHO Fact Sheet link)	Anti-infective drugs as per 2030 Roadmap [1]	Anti-infective drug-based core strategic interventions [1]<br>Selected complementary core interventions [1]	Gaps in anti-infective drugs as per 2030 roadmap [1]
· *L. venezuelensis*<br>· *L. Viannia braziliensis*<br>· *L. (V.) guyanensis*<br>· *L. (V.) panamensis*<br>· *L. (V.) peruviana*<br>https://www.who.int/health-topics/leishmaniasis	EML: IV, 50 mg powder for injection in vial as liposomal complex.<br>**Paromomycin**<br>EML: IM, 750 mg paromomycin as sulfate.<br>**Miltefosine**<br>EML: 10 mg and 50 mg tablet	For dose regimens and ranking of treatment options by causative species and region by the WHO Expert Committee on the Control of Leishmaniases, 2010 see [33]<br>**Selected complementary core interventions [1]**<br>· Vector control<br>· Early diagnosis and treatment	
**Scabies**<br>and other ectoparasites<br>· *Sarcoptes scabiei var hominis*<br>Scabies https://www.who.int/news-room/fact-sheets/detail/scabies	**Ivermectin**<br>EML: 3mg tablet (scored)<br>**Permethrin**<br>EML: topical, 1% lotion and 5% cream<br>**Benzyl benzoate**<br>EML: topical, 25% lotion<br>**Malathion ointment**<br>**Sulfur ointment**	**Preventive chemotherapy [1]**<br>· MDA with ivermectin.<br>· Topical scabicides<br>WHO will be preparing guidelines taking into consideration the outcome of an informal consultation (World Health Organization, 2020b)<br>**Case management [1]**<br>· Topical scabicides (permethrin, benzylbenzoate, malathion and sulfur ointment)<br>· Ivermectin<br>· Specialist case management of crusted scabies cases<br>**Selected complementary core interventions [1]**<br>· Treatment of household contacts<br>· WASH	Determine efficacy of single dose IVM for programmatic use and safe dose in children <15 kg, <90 com or <5 years; Identify alternative strategies for ivermectin MDA including for loiasis co-endemic areas; Evaluate moxidectin [34]
**Buruli ulcer**<br>· *Mycobacterium ulcerans*<br>https://www.who.int/news-room/fact-sheets/detail/buruli-ulcer-(mycobacterium-ulcerans-infection)	**Rifampicin**<br>EML: not listed for the indication<br>**Clarithromycin**<br>EML: not listed for the indication<br>**Moxifloxacin**<br>EML: not listed for the indication	**Case management**<br>**Direct Observed Treatment after laboratory confirmed diagnosis**<br>· Any age, including in pregnancy: Oral rifampicin (10 mg/kg) daily for 8 weeks and oral clarithromycin (7.5 mg/kg) twice daily for 8 weeks (including for pregnant women)<br>· Adults only: Oral rifampicin (10 mg/kg) once daily for 8 weeks and oral moxifloxacin (400 mg) by mouth once daily for 8 weeks.<br>**Selected complementary core interventions** [1]<br>· Surgery (debridement, skin grafting, scar revision)	New treatment options with reduced treatment duration and lower toxicity, especially for children.

NTD, species (WHO Fact Sheet link)	Anti-infective drugs as per 2030 Roadmap [1]	Anti-infective drug-based core strategic interventions [1]<br>Selected complementary core interventions [1]	Gaps in anti-infective drugs as per 2030 roadmap [1]
		· In case of joint involvement or movement limitation, appropriate positioning with frequent exercise. [35]	
**Actinomycetoma**<br>· Actinomycetoma<br>o *Actinomadure madura*<br>o *A. pelletieri*<br>o *Nocardia brasiliensis*<br>https://www.who.int/health-topics/mycetoma-chromoblastomycosis-and-other-deep-mycoses	**None specified**<br>EML: Indication not included	**Case management**<br>· long term treatment with antibiotic combinations<br>· Wound cleaning, dressing<br>**Selected complementary core interventions [1]**<br>· WASH<br>· Protective clothing and shoes	Better treatment regimens (shorter duration, higher efficacy).
**Eumycetoma**<br>o *Madurella mycetomatis*<br>o *Mycetoma mycetomatis*<br>https://www.who.int/health-topics/mycetoma-chromoblastomycosis-and-other-deep-mycoses	**Itraconazole**<br>EML: Indication not included	**Case management**<br>· Antifungals (mainly itraconazole 400 mg/day) – combined with surgery<br>· Wound cleaning, dressing<br>**Selected complementary core interventions [1]**<br>· WASH<br>· Protective clothing and shoes	Better treatment regimens (shorter duration, higher efficacy).
**Chromoblastomycosis**<br>· *Cladophialophora carrionii*<br>· *C. bantiana*<br>· *Fonsecaea pedrosoi*<br>· *Fonsecaea compacta*<br>· *Phialophora verrucosa*<br>**Other Deep Mycoses**<br>· *Paracoccidioides spp.*<br>· *Sporothrix spp.*	**Itraconazole**<br>EML chromoblastomycosis, paracoccidioidomycosis, sporotrichosis: oral liquid, 10 mg per mL,<br>oral solid 100 mg<br>**Amphotericin B**<br>EML sporotrichosis: 50 mg powder for injection (as	**Case management [1]**<br>· No “gold standard” treatment; treatment options include antifungals (itraconazole), physical therapies, immune adjuvants and surgery for minor lesions<br>· Treatment of choice for paracoccidioidomycosis and sporotrichosis: itraconazole<br>**Selected complementary core interventions [1]**<br>· WASH<br>· Protective clothing and shoes	Prospectively obtained effectiveness of itraconazole and other antifungals;<br>Improved treatment regimens (shorter duration and increased efficacy)

NTD, species (WHO Fact Sheet link)	Anti-infective drugs as per 2030 Roadmap [1]	**Anti-infective drug-based core strategic interventions [1]**<br>**Selected complementary core interventions [1]**	Gaps in anti-infective drugs as per 2030 roadmap [1]
https://www.who.int/health-topics/mycetoma-chromoblastomycosis-and-other-deep-mycoses	deoxycholate or liposomal complex)		
**Dengue and Chikungunya**<br>· Flavivirus Dengue virus<br>· Alphavirus Chikungunya virus<br>https://www.who.int/health-topics/dengue-and-severe-dengue<br>https://www.who.int/health-topics/chikungunya	None	NA<br>**Selected complementary core interventions** [1]<br>· WASH<br>· Vector control<br>· Symptomatic treatment [1, 36, 37]	Anti-viral drugs.
**Echinococcosis [World Health Organization , 2019a]**<br>· *Echinococcus granulosus*<br>· *Echinococcus multilocularis*<br>https://www.who.int/health-topics/echinococcosis	**Albendazole**<br>EML: 400 mg tablet (chewable).<br>**Mebendazole**<br>EML: Solid: 100 mg tablet (chewable); 500 mg tablet (chewable)	**Case management**<br>· Treatment with albendazole or mebendazole Albendazole (drug of choice) 10-15mg/kg/day, in two divided doses, with a fat rich meal to increase its bioavailability. Mebendazole may be used at 40-50mg/kg daily, in three divided doses if albendazole is not available or not tolerated.<br>· Other options for cystic echinococcosis include percutaneous methods + albendazole prophylaxis with the PAIR (Puncture, Aspiration, Injection, Re-aspiration) technique, standard catheterization, or the modified catheterization technique, surgery (cyst removal +albendazole prophylaxis, and “watch and wait”<br>· Other option for alveolar echinococcosis: curative surgery [1, 38, 39, 40]<br>**Selected complementary core interventions (WHO, 2020): ‘One Health’ approach**<br>**In collaboration with veterinary and food safety authorities**<br>· WASH<br>· Periodic deworming of dogs with praziquantel<br>· Livestock vaccination<br>· where feasible, anthelminthic baiting of foxes. [1, 39, 40, 41]	Identification of optimal albendazole treatment courses (indicates that drugs with improved efficacy would add value).

<table>
<tr><th>NTD, species (WHO Fact Sheet link)</th><th>Anti-infective drugs as per 2030 Roadmap [1]</th><th>Anti-infective drug-based core strategic interventions [1]
Selected complementary core interventions [1]</th><th>Gaps in anti-infective drugs as per 2030 roadmap [1]</th></tr>
<tr><td>Foodborne trematodiases
· Clonorchis sinensis
· Dicrocoelium dendriticum
· D. hospes
· Fasciola hepatica
· F. gigantica
· Fasciolopsis buski
· Heterophyes heterophyes
· Metagonimus yokogawai
· Opisthorchis viverrine
· O. felineus
· Paragonimus westermani
· P. kellicotti
https://www.who.int/health-topics/foodborne-trematode-infections</td><td>Praziquantel
EML: 250 mg and 600 mg tablet.
Triclabendazole
EML: 250 mg tablet.</td><td>Preventive chemotherapy [1]
· Small liver flukes and Paragonimus spp.: MDA with praziquantel
· Fasciola spp and Paragonimus spp: MDA with triclabendazole
Case management (World Health Organization, 2007; 2008; 2011; 2020a)
· Praziquantel
· Triclabendazole
Selected complementary core interventions [1]:
· WASH
· Veterinary public health: treatment of livestock and other domestic animals,
· Management practices in fish farming
· Snail control
· Outbreak investigation and control [36]</td><td>None</td></tr>
<tr><td>Taeniasis and cysticercosis [World Health Organization , 2018b]
· Taenia solium
https://www.who.int/health-topics/taeniasis-and-cysticercosis</td><td>Albendazole
EML: 400 mg tablet (chewable)
Praziquantel
EML: 600 mg and 150 mg tablet
Niclosamide
EML: 500 mg tablet (chewable)</td><td>Preventive Chemotherapy
· Endemic populations: Single dose praziquantel (10 mg/kg) or, if active surveillance and medical referral of neurological adverse events is in place, niclosamide (2 g, dose adjusted for children), or albendazole 400 mg/day for 3 consecutive days [1, 42].
· Endemic population in communities with school based preventive chemotherapy for soil-transmitted helminths and reporting system with active surveillance and medical referral of neurological adverse events: co-administration of single dose praziquantel (10 mg/kg) and single dose albendazole (400 mg) to school age children [42].
Case management
· Taeniasis: Single administration of praziquantel (10 mg/kg) or niclosamide (single dose, adults and children >6 years: 2 g, children 2-6 years 1g, children < 2 years 0.5 g, after light meal followed after 2 hours by laxative) [43]</td><td>Efficacy of current treatment strategies</td></tr>
</table>

NTD, species (WHO Fact Sheet link)	Anti-infective drugs as per 2030 Roadmap [1]	Anti-infective drug-based core strategic interventions [1]<br>Selected complementary core interventions [1]	Gaps in anti-infective drugs as per 2030 roadmap [1]
		· Neurocysticercosis: long courses of praziquantel and/ or albendazole and supporting therapy with corticosteroids and/or antiepileptic medicines. Doses and duration depending on number, size, location and developmental stage of the cysts, surrounding inflammatory edema, acuteness and severity of signs or symptom. Usual doses 15 mg/ kg/day albendazole and 50 mg/kg/day praziquantel, divided in two to three daily doses; proposed length of treatment from one to two weeks for parenchymal and ≥ 1 month for subarachnoid lesions. [40, 43]<br>**Selected complementary core interventions [1]:**<br>in collaboration with Food and Agriculture Organization of the United Nations (FAO) and the World Organization for Animal Health (OIE), including<br>· Water, Sanitation and Hygiene (WASH) [1, 43]<br>· Prevention and control in pigs: improved pig farming, pig vaccination with TSOL18 and treatment with oxfendazole, meat inspection and processing, [43]	

References

1. World Health Organization. Ending the neglect to attain the Sustainable Development Goals – A road map for neglected tropical diseases 2021–2030. 2020:177. https://www.who.int/neglected_diseases/WHONTD-roadmap-2030/en/.
2. World Health Organization. Eradicating Guinea-worm disease - the last painful steps. 1998. https://apps.who.int/iris/handle/10665/64506.
3. World Health Organization. Summary report of a consultation on the eradication of yaws, 5-7 March 2012, Morges, Switzerland. 2012; WHO/HTM/NTD/IDM/2012.2. https://apps.who.int/iris/handle/10665/75528.
4. World Health Organization. Eradication of yaws: a guide for programme managers. World Health Organization. 2018. https://apps.who.int/iris/handle/10665/259902.
5. World Health Organization: Yaws. https://www.who.int/news-room/fact-sheets/detail/yaws (2022). Accessed 7/19/2022 2022.
6. World Health Organization. WHO interim guidelines for the treatment of gambiense human African trypanosomiasis. 2019. https://apps.who.int/iris/handle/10665/326178.
7. World Health Organization Regional Office for South-East Asia. Guidelines for the diagnosis, treatment and prevention of leprosy. 2018. https://apps.who.int/iris/handle/10665/274127.

8. Mectizan Expert Committee and APOC Technical Consultative Committee: Recommendations for the treatment of onchocerciasis with Mectizan in areas co-endemic for onchocerciasis and loiasis. https://mectizan.org/wp-content/uploads/2018/06/englishmectccloarecs-june04.pdf (2004). Accessed 7/11/2022 2022.
9. World Health Organization. Community-directived treatment with ivermectin: a pratical guide for trainers of community-directed distributors. 1998. https://apps.who.int/iris/handle/10665/275546.
10. UNDP/World Bank/WHO Special Programme for Research and Training in Tropical Diseases. Guidelines for rapid assessment of *Loa loa*. 2002; TDR/IDE/RAPLOA/02.1. https://apps.who.int/iris/handle/10665/67250.
11. Crompton D.W.T., World Health Organization: Preventive chemotherapy in human helminthiasis : coordinated use of anthelminthic drugs in control interventions : a manual for health professionals and programme managers. Geneva: World Health Organization; 2006.
12. World Health Organization. Report of the 1st meeting of the WHO onchocerciasis technical advisory subgroup, Varembé Conference Centre, Geneva, Switzerland, 10-12 October 2017. 2018. https://apps.who.int/iris/handle/10665/273705.
13. World Health Organization. Report on the fourth meeting of the WHO onchocerciasis technical advisory subgroup: virtual meeting, 28-29 October 2020. 2021. https://apps.who.int/iris/handle/10665/348383.
14. World Health Organization African Programme for Onchocerciasis Control. Community-directed treatment with ivermectin: a pratical guide for trainers of community-directed distributors. African Programme for Onchocerciasis Control. 1998. https://apps.who.int/iris/handle/10665/275546.
15. Pan American Health Organization. Guidelines for the diagnosis and treatment of Chagas disease. 2019. https://iris.paho.org/bitstream/handle/10665.2/49653/9789275120439_eng.pdf.
16. World Health Organization. Chagas disease (American trypanosomiasis) — factsheet (revised in August 2012). Weekly Epidemiological Record = Relevé épidémiologique hebdomadaire. 2012;87 51-52:519-22. https://apps.who.int/iris/handle/10665/242006.
17. World Health Organization and Expert Committee on the Control and Surveillance of Human African Trypanosomiasis. Control and surveillance of human African trypanosomiasis: report of a WHO expert committee. Technical Report Series. 2013. https://apps.who.int/iris/handle/10665/95732.
18. World Health Organization. WHO guideline for the treatment of visceral leishmaniasis in HIV co-infected patients in East Africa and South-East Asia. 2022. https://apps.who.int/iris/handle/10665/354703.
19. World Health Organization. WHO guideline for the treatment of visceral leishmaniasis in HIV co-infected patients in East Africa and South-East Asia: web annex B: evidence-to-decision tables. 2022. https://apps.who.int/iris/handle/10665/354547.

20. World Health Organization & Cochrane Response. WHO guideline for the treatment of visceral leishmaniasis in HIV co-infected patients in East Africa and South-East Asia: web annex A: a systematic review on the treatment of visceral leishmaniasis in HIV-Leishmania co-infected persons in East Africa and South-East Asia. 2022. https://apps.who.int/iris/handle/10665/354546.
21. Solomon AW, World Health O, London School of H, Tropical M, International Trachoma I. Trachoma control : A guide for programme managers. 2006. https://apps.who.int/iris/handle/10665/43405.
22. World Health Organization. Report of the 2nd Global scientific meeting on trachoma: Geneca 25-27 August, 2003. 2003. https://apps.who.int/iris/handle/10665/329076.
23. World Health Organization. Technical consultation on trachoma surveillance: meeting report. September 11-12, 2014, Task Force for Global Health, Decatour, USA. 2015. https://apps.who.int/iris/handle/10665/174085.
24. World Health Organization. Report of the 4th global scientific meeting on Trachoma: Geneva, 27-29 November 2018. 2019. https://apps.who.int/iris/handle/10665/325121.
25. World Health Organization. Guideline: alternative mass drug administration regimens to eliminate lymphatic filariasis. 2017; WHO/HTM/NTD/PCT/2017.07. https://apps.who.int/iris/handle/10665/259381.
26. Boussinesq M, Gardon J, Gardon-Wendel N, Chippaux JP. Clinical picture, epidemiology and outcome of *Loa*-associated serious adverse events related to mass ivermectin treatment of onchocerciasis in Cameroon. Filaria J. 2003;2 Suppl 1:S4; doi: 10.1186/1475-2883-2-S1-S4. http://www.ncbi.nlm.nih.gov/pubmed/14975061.
27. Boussinesq M, Kamgno J, Pion SD, Gardon J. What are the mechanisms associated with post-ivermectin serious adverse events? Trends Parasitol. 2006;22 6:244-6; doi: 10.1016/j.pt.2006.04.006. https://www.ncbi.nlm.nih.gov/pubmed/16632406.
28. World Health Organization. Provisional strategy for interrupting lymphatic filariasis transmission in loiasis-endemic countries: report of the meeting on lymphatic filariasis, malaria and integrated vector management, Accra, Ghana, 5-9 March 2012. 2012; WHO/HTM/NTD/PCT/2012.6. https://apps.who.int/iris/handle/10665/75139.
29. World Health Organization. WHO guideline on control and elimination of human schistosomiasis. 2022. https://apps.who.int/iris/handle/10665/351856.
30. World Health Organization. Guideline: preventive chemotherapy to control soil-transmitted helminth infections in at-risk population groups. 2017. https://apps.who.int/iris/handle/10665/258983.
31. World Health Organization. Rabies vaccines: WHO position paper, April 2018 - Recommendations. Vaccine. 2018;36 37:5500-3; doi: 10.1016/j.vaccine.2018.06.061. https://www.ncbi.nlm.nih.gov/pubmed/30107991.

32. World Health Organization. WHO expert consultation on rabies: third report. 2018. https://apps.who.int/iris/handle/10665/272364.
33. WHO Expert Committee on the Control of the Leishmaniases & World Health Organization. Control of the leishmaniases: Report of a meeting of the WHO Expert Commitee on the Control of Leishmaniases, Geneva, 22-26 March 2010. WHO technical report series. 2010. https://apps.who.int/iris/handle/10665/44412.
34. World Health Organization. WHO informal consultation on a framework for scabies control: World Health Organization Regional Office for the Western Pacific: Manila, Philippines, 19–21 February 2019: meeting report. 2020. https://apps.who.int/iris/handle/10665/333154.
35. World Health Organization. Treatment of *Mycobacterium ulcerans* disease (buruli ulcer): guidance for health workers. 2012. https://apps.who.int/iris/handle/10665/77771.
36. World Health Organization. Foodborne disease outbreaks: guidelines for investigation and control. 2008. https://apps.who.int/iris/handle/10665/43771.
37. World Health Organization Regional Office for South-East Asia. Guidelines on clinical management of chikungunya fever. 2008. https://apps.who.int/iris/handle/10665/205178.
38. Brunetti E, Kern P, Vuitton DA. Expert consensus for the diagnosis and treatment of cystic and alveolar echinococcosis in human. Acta Trop. 2010;114 1:1-16; doi: 10.1016/j.actatropica.2009.11.001. https://pubmed.ncbi.nlm.nih.gov/19931502/.
39. World Health Organization. Fact sheet on echinococcosis (updated May 2019) Wkly Epidemiol Rec. 2019;94 48:574-9. https://apps.who.int/iris/handle/10665/330007.
40. World Health Organization. WHO guidelines on management of *Taenia solium* neurocysticercosis. 2021. https://apps.who.int/iris/handle/10665/344802.
41. World Health Organization Food and Agriculture Organisation of the United Nations & World Organisation for Animal Health. Foodborne parasitic infections: cystic and alveolar echinococcosis. 2021. https://apps.who.int/iris/handle/10665/341874.
42. Pan American Health Organization & World Health Organization. Guideline for preventive chemotherapy for the control of *Taenia solium* taeniasis. 2021; doi: https://doi.org/10.37774/9789275123720. https://iris.paho.org/handle/10665.2/54800.
43. World Health Organization. Fact sheet on taeniasis/ cysticercosis (updated February 2018). Wkly Epidemiol Rec. 2018;93 46:630-2. https://apps.who.int/iris/handle/10665/275879.

44. World Health Organization. The selection and use of essential medicines: Report of the WHO Expert Committee on Selection and Use of Essential Medicines, 2021 (including the 22nd WHO model list of essential medicines and the 8th WHO model list of essential medicines for children). 2021. https://apps.who.int/iris/handle/10665/351172.

EML: WHO Model List of Essential Medicines, 2021 [44], IM intramuscular, IV intravenous, PC: preventive chemotherapy, WASH: Clean water, sanitation and hygiene.

Table S3: Cure and egg reduction rates in Phase 2 studies evaluating moxidectin efficacy against *Strongyloides stercoralis, Trichuris trichiura* and concomitant helminths; and against *Schistosoma haematobium* and *S. mansoni*

Helminth	Treatment* (n analysed)	CR (%) (95% CI)	% GM ERR/LRR (95% CI)	Additional information
S. stercoralis	IVM 200 µg/kg (62)	95.1 (86.5- 99.0)		Laos<br>Adolescents and adults
	Moxi 8 mg (63)	93.6 (84.5-98.2)		Randomized, single blind, single dose
Hookworm	IVM 200 µg/kg (34)	55.9 (52.1-84.7)	79.4 (61-88)	Baermann assay, Kato-Katz, 2 samples (blinded)
	Moxi 8 mg (37)	56.7 (55.9-79.7)	74.6 (61-90)	21-25 days post-Tx
Opistorchis. viverrini	IVM 200 µg/kg (46)	6.5 (6.4-25.4)	0 (–40-2)	ISRCTN11983645
	Moxi 8 mg (56)	17.8 (11.2-32.2)	12.5 (–2-30)	[2]
T. trichiura	Moxi 8mg + Alb 400mg (197)	50.8 (43.6–57.9)	98.5 (98.0–98.9)	Tanzania<br>12-18 years
	Alb 400mg + OxP 25 mg/kg (200)	83.0 (77.1–87.9)	99.8 (99.6–99.9)	Randomized, single blind, single dose
	Moxi 8mg + Tri [200mg <15 yrs, 400 mg ≥15 yrs] (119)	22.7 (15.5–31.3)	91.6 (88.2–93.9)	Kato-Katz, 2 samples, 2 smears each (blinded)
	Moxi 8 mg (118)	14.4 (8.6–22.1)	83.2 (77.9–87.6)	14-21 days post-Tx
**Hookworm**	Moxi 8mg + Alb 400mg (95)	76.8 (66.2–85.4)	98.9 (98.0- 99.5)	ISRCTN20398469
	Alb 400mg + OxP 25 mg/kg (94)	75.9 (65.3–84.6)	98.6 (97.4- 99.3)	[3]
	Moxi 8mg + Tri [200mg <15 yrs, 400 mg ≥15 yrs] (55)	88.2 (76.1–95.6)	99.4 (98.7- 99.8)	
	Moxi 8 mg (51)	34.0 (20.8–49.3)	86.8 (72.7–93.9)	
A. lumbricoides	Moxi 8mg + Alb 400mg (133)	96.6 (91.5–99.1)	>99.9 (99.98–99.99)	
	Alb 400mg + OxP 25 mg/kg (129)	96.6 (91.4–99.1)	>99.9 (99.97–99.99)	
	Moxi 8mg + Tri [200mg <15 yrs, 400 mg ≥15 yrs] (77)	97.1 (90.2–99.6)	>99.9 (99.9–100.0)	
	Moxi 8 mg (71)	98.4 (91.4–99.9)	>99.9 (99.9–100.0)	
T. trichiura	Placebo (40)	12.5	45.9	Tanzania

Helminth	Treatment* (n analysed)	CR (%) (95% CI)	% GM ERR/LRR (95% CI)	Additional information
		(4.2–26.8)	(11.3–67.0)	16-18 years
	Moxi 8 mg (41)	46.3 (30.7–62.6)	94.3 (87.8–97.5)	Randomized, single blind, single dose
	Moxi 16 mg (38)	50.0 (33.4–66.6	95.0 (90.3–97.6)	Kato-Katz, 2 samples, 2 smears, 13-20 days post-Tx,
	Moxi 24 mg (41)	43.9 (28.5–60.3)	95.7 (91.8–97.8)	Blinded
	Moxi 8 mg + Alb 400 mg (40)	62.5 (45.8–77.3)	97.4 (94.2–99.0)	NCT03501251
	Moxi 16 mg + Alb 400 mg (42)	61.9 (45.6–76.4)	98.4 (96.7–99.3)	[4]
	Moxi 24 mg + Alb 400 mg (39)	69.2 (52.4–83.0)	98.6 (97.2–99.4)	
Hookworm	Placebo (12)	25	68.6	
	Moxi 8 mg (12)	50	88.2	
	Moxi 16 mg (11)	25	73.7	
	Moxi 24 mg (12)	33.3	81.3	
	Moxi 8 mg + Alb 400 mg (10)	81.8	99.7	
	Moxi 16 mg + Alb 400 mg (15)	80	93.8	
	Moxi 24 mg + Alb 400 mg (10)	90	99.6	
A. lumbricoides	Placebo (5)	20	89.3	
	Moxi 8 mg (4)	100	100	
	Moxi 16 mg (4)	75	100	
	Moxi 24 mg (6)	100	100	
	Moxi 8 mg + Alb 400 mg (7)	100	100	
	Moxi 16 mg + Alb 400 mg (4)	75	100	
	Moxi 24 mg + Alb 400 mg (7)	100	100	
S. stercoralis	Placebo (29)	14 (4–32)	27·0 (–2·2 to 48·3)	Laos Adults
	Moxi 2 mg (30)	73 (54–88)	98·4 (93·7–99·9)	Randomized, single blind, single dose
	Moxi 4 mg (29)	90 (73–98)	99·4 (98·1–100·0)	Baermann assay, Kato-Katz, 2 samples, 2 smears
	Moxi 6 mg (32)	84 (67–95)	99·8 (99·3–100·0)	28 days post-Tx
	Moxi 8 mg (29)	83 (64–94)	97·8 (93·2–99·9)	NCT04056325
	Moxi 10 mg (30)	97	98·5	[5]

Helminth	Treatment* (n analysed)	CR (%) (95% CI)	% GM ERR/LRR (95% CI)	Additional information
		(83–100)	(95·0–100·0)	
	Moxi 12 mg (30)	87 (69–96)	98·6 (95·7–99·9)	
Hookworm	Placebo (17)	0		
	Moxi 2 mg (17)	0		
	Moxi 4 mg (18)	11		
	Moxi 6 mg (21)	14		
	Moxi 8 mg (17)	0		
	Moxi 10 mg (20)	10		
	Moxi 12 mg (21)	29		
O. viverrini	Placebo (17)	14		
	Moxi 2 mg (17)	12		
	Moxi 4 mg (18)	0		
	Moxi 6 mg (21)	4		
	Moxi 8 mg (17)	-5		
	Moxi 10 mg (20)	-5		
	Moxi 12 mg (21)	14		
S. mansoni	Moxi 8 mg (31)	12.9 0.03–0.3)	70.9 0.4–0.9)	Côte d'Ivoire 12-18 years
	Syn 3 daily doses + PZQ 40 mg/kg (26)	27.0 0.1–0.5)	77.6 0.5–1.1)	Randomized, single blind
	Syn 3 daily doses (30)	6.7 0.01–0.2)	64.9 0.4–0.8)	Kato-Katz, 2 samples, 2 smears, 21 days post-Tx
	PZQ 40 mg/kg (29)	27.6 0.1–0.5)	87.5 0.8–1)	
S. hematobium	Moxi 8 mg (27)	14.8 (0.04–0.3)	8.7 (-0.4–0.6)	3 urine samples, urine filtration
	Syn 3 daily doses + PZQ 40 mg/kg (30)	60 (0.4–0.8)	96 (0.8–1.0)	ISRCTN 63657086
	Syn 3 daily doses (27)	11.1 (0.02–0.3)	0 (-0.8–0.6)	[6]
	PZQ 40 mg/kg (26)	38.5 (0.2–0.6)	93.5 (0.8–1.0)	

* single dose unless otherwise specified.

Alb albendazole, CR cure rate, GM ERR geometric mean-based egg reduction rate, IVM ivermectin, LRR larval reduction rate, Moxi moxidectin, OxP oxantel pamoate, PZQ praziquantel, Syn synriam (150 mg arterolane + 750 mg piperaquine, Tri tribendimidine, Tx treatment

1 Anti-infective drugs for diseases for which preventive chemotherapy is the main strategic core intervention strategy

The strategic core intervention for many NTDs is or includes preventive chemotherapy (PC), i.e., drug administration to specified (eligible, at risk) populations without individual diagnosis. These include NTDs targeted for eradication (yaws), for elimination (i.e., interruption of transmission, onchocerciasis, leprosy), for elimination as a public health

problem (LF, schistosomiasis, STH, trachoma) and for control (food-borne trematodiases, scabies, taeniasis and cysticercosis) by or beyond 2030 (Table S2) [7].

Many drugs were originally developed for veterinary use. Prior registration for veterinary use accelerates and, to some extent, reduces costs and risk for development for human use because of significant overlap between regulatory requirements for veterinary and human drugs for non-clinical studies to characterize the drug toxicity profile (dose-response relationship, affected organs, reversibility of effects [8]). Continued large scale use of the same drug for animal and human health and development of resistance is one of the aspects that require 'One Health' approaches [7, 9]. Monitoring drug susceptibility, including variability of response, is a challenge for PC programmes given the lack of suitable diagnostics for this and other purposes. The WHO NTD department has formed a Diagnostics Technical Advisory Committee. WHO makes the target product profiles and preferred product characteristics emerging from this committee as well as others available within its 'Global Observatory on Health Research and Development (https://www.who.int/observatories/global-observatory-on-health-research-and-development, https://www.who.int/observatories/global-observatory-on-health-research-and-development/analyses-and-syntheses/target-product-profile/who-target-product-profiles) [7, 10].

1.1 Ivermectin

Ivermectin, a macrocyclic lactone discovered in 1975 [11], is a semisynthetic anthelmintic derived from avermectin, a fermentation product of *Streptomyces avermitilis*. Macrocyclic lactones have activity against a broad spectrum of endo- and ecto-parasites. They are agonists of the glutamate-gated chloride channel, present in the neurons and pharyngeal muscles of nematodes and arthropods, but not of humans. Activation of the channel inhibits movement and pharyngeal pumping, leading to paralysis [12, 13, 14]. The role of the human immune system in the efficacy of ivermectin against filarial nematodes is still under investigation [15, 16, 17, 18]. Consideration for development for onchocerciasis started in 1978 [19, 20] before introduction into the veterinary market in 1981 [21, 22].

In heavily *Loa loa* infected individuals, ivermectin treatment can result in severe and potentially fatal adverse reactions [23, 24, 25]. This prohibits ivermectin use in loiasis endemic areas that are not onchocerciasis meso- and hyperendemic due to the overall risk-benefit for the population [26]. Until the advent of drugs safe in *Loa loa* co-infected individuals, alternative treatment strategies are needed [27].

Concern has been raised about *O. volvulus* 'suboptimal response' or potentially emerging resistance to ivermectin's embryostatic effect (i.e., time to resumption of microfilariae production and release by the macrofilariae) in some regions after long term use of ivermectin [23, 28, 29, 30, 31, 32, 33]. However, genome-wide association analyses suggest that *O. volvulus* response to ivermectin is a polygenically determined quantitative trait with different identical or related molecular pathways determining the extent of ivermectin response in different *O. volvulus* populations [34]. Furthermore, 'suboptimal response' to ivermectin was observed in some *O. volvulus* infected individuals in areas without ivermectin treatment history [35, 36]. This highlights the need to include variability of response in monitoring of drug response and interpretation of the results.

1.2 Albendazole, mebendazole and triclabendazole

Albendazole, mebendazole, and triclabendazole belong to the class of benzimidazoles. The class was originally developed as plant fungicides and later as veterinary anthelminthics [37]. Benzimidazole exposure results in inhibition of beta tubulin polymerase causing disruption of cytoplasmic microtubule formation [12, 38]. This leads to the killing of adult stages of gut-

dwelling helminths, as well as sterilization or killing of the eggs and larvae [37]. **Albendazole** is a broad-spectrum anthelminthic, first approved for use in humans in 1982. In its current formulation for human use it is poorly absorbed [38]. **Mebendazole** is also a broad-spectrum anthelminthic. Its mode of action involves multiple targets including glucose uptake in nematodes and cestodes in addition to inhibition of tubulin polymerization [39]. **Triclabendazole** is a narrow-spectrum anthelminthic originally developed for animal fasciolosis. Its mode of action is not completely understood. Triclabendazole and its metabolites are thought to cross the tegument of the immature and adult worms, resulting in resting membrane potential alternation, interference with microtubule structure and function, inhibition of protein synthesis and ultimately death [40, 41]. Development for human fascioliasis, one of the most widespread foodborne trematode infections, was initiated in the 1990ies by WHO in collaboration with Chemische Industrie Basel (CIBA) after a fascioliasis epidemic in Iran in 1989. Regulatory approval for this indication was obtained in Egypt in 1997 and in France in 2002 [41] . Triclabendazole was approved for treatment of fascioliasis in patients 6 years or older by the US FDA in 2019 [40]. Triclabendazole is currently the only drug available able to kill early immature and adult *Fasciola hepatica* [41]. Triclabendazole is under consideration for repurposing for drug-resistant bacterial infections [42].

1.3 Diethylcarbamazine

Diethylcarbamazine (DEC), discovered in 1947, is a piperazine derivative anthelmintic evaluated for its efficacy and safety for onchocerciasis beginning in the 1950ies [43]. *In vitro* experiments at therapeutic concentrations demonstrated the loss of the microfilarial sheath with subsequent damage of organelles and apoptosis of the filarial nematode *Wuchereria bancrofti* [44], the cause of 90% of lymphatic filariasis cases globally [45]. The Global Programme to Eliminate Lymphatic Filariasis advocated for two elimination strategies in areas not co-endemic for either loiasis or onchocerciasis: annual mass drug administration (MDA) of a single dose of DEC or DEC with albendazole, estimated to require 4-6 years, or substitution of table/cooking salt by DEC-fortified salt (0.2-0.4% w/w) estimated to require 6-12 months [Ottesen et al. 1997]. By 2020, MDA had been implemented in at least one endemic area in 69/72 endemic countries and 17/72 countries had met the criteria for elimination of LF as a public health problem [46]. The data from studies and pilot/small scale use of DEC-fortified salt were encouraging [47, 48]. However, implementation for large scale LF control may have been limited [49] to four countries: Taiwan [48], China, where elimination of LF as a public health problem has been partly attributed to the use of DEC-fortified salt [50], Haiti [51], and some areas in India [52, 53, 54].

1.4 Praziquantel

Praziquantel is a chiral pyrazine-isoquinoline derivative discovered in 1972 and first developed for veterinary use. It has a broad spectrum of activity against trematodes and cestodes [55]. The anti-helminthic action is not fully understood and may include binding to calcium channels, tegument disruption, binding and polymerization of actin and exposure of surface membrane antigens [56, 57, 58]. As for other NTDs, the extent to which long-term preventive chemotherapy affects parasite drug susceptibility is unknown [59].

The WHO recommended single dose of 40 mg/kg praziquantel (Table S2) achieves 95%, 94.1% and 86.3% ERR in *Schistosoma. japonicum*, *S. haematobium* and *S. mansoni*, respectively [57]. A dose of 60mg/kg did not increase efficacy against *S. mansoni* or *S. japonicum* [60, 61, 62]. Known limitations of praziquantel are its inactivity against immature parasites [57].

Praziquantel is well tolerated, but in individuals with cysticercosis and cysts in the central nervous system or eyes, the inflammatory reaction to dying *Taenia solium* can result in seizures, and/or cerebral infarction and permanent eye lesion [57].

The commercially available tablets include both (R)-praziquantel (L) with anthelmintic activity and the inactive (S)-praziquantel (D) which contributes to the bitter taste and a 600 mg tablet size that is unsuitable for pre-school children [63]. The Paediatric Praziquantel Consortium (https://www.pediatricpraziquantelconsortium.org/) has developed a paediatric formulation [64, 65].

1.5 Azithromycin

Azithromycin is a macrolide antibiotic with a 15-member lactone ring structure with two sugars attached via a glycosidic bond that is semi-synthetically produced from erythromycin A. It is the single compound in its azalide subclass. Azithromycin binds to the 50S ribosomal subunit at the peptidyl transferase centre, preventing protein synthesis [66, 67]. Besides broad-spectrum activity against Gram-positive and Gram-negative bacteria, azithromycin has activity against the apicomplexan parasites *Toxoplasma gondii* and *Malaria* spp. [67, 68, 69, 70]. Azithromycin can be taken orally and has a safety profile [71, 72] which supports inclusion of pregnant women and children in MDA [7]. The absence of drug-drug interactions, allows integrating azithromycin, ivermectin, diethylcarbamazine and albendazole MDA [71].

Since studies in Papua New Guinea and Ghana [73, 74] showed non-inferiority to penicillin G, azithromycin is the preferred antibiotic for treating yaws [74, 75, 76]. A single 20 mg/kg dose is highly effective in treating trachoma infections [77, 78]. Yaws and trachoma can be co-endemic and research has shown that 20 mg/kg is as effective as 30 mg/kg against Yaws [79].

1.6 Benzathine penicillin

Benzathine penicillin (penicillin G) was discovered in 1951 [80]. It is a bactericidal beta-lactam that inhibits bacterial peptidoglycan transpeptidases, preventing cell wall formation during cell division [81]. While oral formulations are available, benzathine penicillin is frequently administered intravenously or intramuscularly due to its poor oral bioavailability. Slow-release formulations provide effective serum levels measurable for at least 14 days. Benzathine penicillin has a broad spectrum of activity against Gram-positive and Gram-negative bacteria, including the causative agents of yaws and trachoma [75, 80]. Despite the need for trained health professionals and discomfort of injections for the patient, benzathine penicillin mass treatment of cases and contacts was implemented in 46 countries from 1952-1964 and reduced global yaws and other treponematoses burden by 95% [75]. Care must be taken to ensure individuals with known penicillin sensitivity or history of allergic reactions, including anaphylaxis, are excluded and immediate access to required interventions is available to avoid fatalities [64, 65].

1.7 Topical scabicides

1.7.1 Permethrin

Permethrin is a synthetic pyrethroid insecticide, based on pyrethrum extracts, designed to increase insecticidal activity, lower mammalian toxicity and provide the photostability required for agricultural use. Permethrin acts on the nerve cell membrane of arthropods to disrupt the sodium channel current that regulates the polarization of the membrane. This results in delayed repolarization and subsequent paralysis and death of the parasites. Permethrin is an active ingredient of mosquito nets [82, 83, 84, 85, 86].

Permethrin is available in topical products for human use. The WHO EML 2021 lists a 1% lotion and a 5% cream [1]. A permethrin cream (5%) was approved for the treatment of scabies in children two months of age or older by the US FDA in 1989 [87]. Safety in younger infants is an open question [88, 89] The absorption of permethrin through the skin is limited to 2% of the amount applied with the fraction absorbed being eliminated via rapid metabolism [87, 88].

Five percent (5%) permethrin cream applied head-to-toe including in intimate areas is highly effective for treatment of scabies cases and reducing the risk of infection of contacts [90]. However, large scale use in endemic community settings faces a number challenges ranging from cost to individual acceptance and compliance with the treatment regimen [91, 92, 93, 94]. This limits its utility and drives considerations for use only in individuals for which ivermectin is contra-indicated [91, 95, 96].

1.7.2 Benzyl benzoate

Benzyl benzoate is an ester of benzoic acid and benzyl alcohol which is neurotoxic to mites [97]. *In vitro*, 100% of mites were killed after 3 hours of exposure to 25% benzyl benzoate [98]. The drug has been used for scabies since the late 1930ies [99] and is available as 10-25% lotions or emulsions [100, 101]. Characterized as safe in children ≥1 month [102], a 5% topical cream was identified as the treatment of choice for infants >2 months for whom safe and effective ivermectin doses have not been identified and as an option for infants younger than 2 months with application for four hours [96]. Severe skin irritation can occur within minutes of application [101]. An informal consultation on a framework for scabies control was held by WHO in 2019 to review current data and gather expert views. The experts recommended that, in view of the inferior efficacy and higher rate of adverse effects of benzyl benzoate compared to permethrin, benzyl benzoate should only be used when topical treatment is indicated (i.e. for individuals for which ivermectin is not approved) and permethrin is unavailable [91, 95, 96].

1.7.3 Malathion

Malathion is an organophosphate insecticide. Its toxic metabolite malaoxon irreversibly inhibits acetylcholinesterase, resulting in acetylcholine accumulation and disruption of the nervous system function [103, 104, 105].

Malathion, as an aqueous lotion 0.5% w/v, was first developed for human use in the treatment of headlice. The justification for the use of malathion in scabies comes from a study conducted in 1978 that demonstrated an 83% cure rate in a population of 30 individuals with scabies [106]. A 2013 review did not identify sufficient evidence to assess the relative efficacy of malathion and other scabies treatment options [107]. Malathion is listed as an alternative rather than a recommended treatment of scabies in the European recommendations [108]. In 2015 malathion was classified as probably carcinogenic to humans by the WHO International Agency for Research on Cancer [105, 109].

1.7.4 Sulphur ointment

Sulphur is the oldest antiscabietic, reported to have been used already around 25 AD. The reduction of sulphur to hydrogen sulphide by bacteria on the skin results in killing the scabies mite [97]. Topical sulphur treatments for scabies contain between 2-33% sulphur. They were used in the youngest children (at strengths of <10% sulphur), including those under 2 months as well as pregnant women due to a perceived safety profile resulting from the lack of absorption following dermal application [97]. It was recently used during MDA for a scabies outbreak in Ethiopia for treatment of children under 10 years of age and pregnant and breast-feeding women [110].

The efficacy of sulphur is inferior to that of permethrin and ivermectin [97]. Sulphur is listed as an alternative rather than a recommended treatment of scabies in the European recommendations [108]. Its use can be limited by its strong and unpleasant odour that results from the formation of hydrogen sulphide as well as the fact that it can stain clothing [111].

2 Anti-infective drugs for diseases for which case management is the main control- and elimination strategy

2.1 Antibiotics

2.1.1 Rifampicin

Rifampicin, derived from *Nocardia mediterranei*, was discovered in 1957 and synthesized in 1965. Activity against *Mycobacterium tuberculosis* was determined *in vitro* and *in vivo* in mice, guinea pigs, and rabbits before trials in humans, initiated in 1966, demonstrated efficacy against *M. tuberculosis* strains resistant against all other anti-tuberculosis drugs at the time [112]. Rifampicin inhibits bacterial DNA-dependent RNA synthesis, thus preventing transcription and inhibiting bacterial protein synthesis, resulting in a bactericidal effect. Differences in antimicrobial activity against Gram-positive and Gram-negative bacteria are not related to different binding sites on the RNA polymerase but to other factors like efflux pumps. Different mechanisms of resistance have been described, including changes of the binding pocket of rifampicin on the RNA polymerases [113]. Rifampicin activates the nuclear pregnane X receptor increasing the expression of genes whose products are involved in drug metabolism and transport, inducing cytochrome P450 2B6 (CYP2B6), CYP 3A4 and P-glycoprotein. The resulting potential for drug-drug interaction needs to be considered in particular in treatment of TB and HIV co-infected patients [114].

A systematic review concluded that a single rifampicin dose reduced leprosy incidence in contacts of patients in the first two years by 57% [115]. Rifampicin-associated adverse effects include flu-like syndrome, gastrointestinal and dermatological events, as well as hepatitis and cholestasis [112].

2.1.2 Dapsone

Dapsone, discovered in 1908, is a sulphone bacteriostatic antibiotic and anti-inflammatory agent. Its bacteriostatic effect is due to its sulphonamide-like ability to compete with para-aminobenzoic acid for the active site of dihydropteroate synthetase, thus inhibiting dihydrofolic acid synthesis. The anti-inflammatory effect may be based on different mechanisms including inhibition of chemokine production, neutrophil response to chemotactic signals and adherence to endothelium, generation of toxic and oxygen-derived radicals. Dapsone is metabolized by cytochrome P450, making it susceptible to drug-drug interactions. The resulting hydroxylamines are considered responsible for the dose-dependent adverse effects agranulocytosis, methemoglobinemia and haemolysis. The most important other dose-dependent adverse effects occurring at low frequency within the dose range considered effective for leprosy is peripheral neuropathy. Life-threatening dapsone hypersensitivity syndrome occurs in 0.5% to 3.6% of patients [116]. This syndrome has been linked to *HLA-B*13-1* polymorphism which could provide a path to pre-treatment screening [117, 118, 119].

Dapsone resistance of *Mycobacterium leprae* has been known since 1977 [120, 121] and has been attributed to a mutation of folP1, a gene coding for dihydropteroate synthase [122].

2.1.3 Clofazimine

Clofazimine, first described in 1957, is a riminophenazine antibiotic. It is active against slowly and rapidly growing mycobacteria, as well as many other Gram-positive bacteria *in vitro* but not against Gram-negative bacteria. The primary site of action has been proposed to

be the outer membrane. Putative targets include ion transporters and the bacterial respiratory chain. Besides anti-infective activity, clofazimine also has anti-inflammatory properties benefitting the treatment of leprosy. Its high lipophilicity enables clofazimine to accumulate in skin and nerves which contributes to its efficacy against erythema nodosum leprosum. Adverse effects of clofazimine include reversible discolouration of the skin and conjunctiva and gastrointestinal events which are usually mild to moderate but may in some cases be severe including bleeding, splenic infarction, and bowel obstruction [123, 124]. The primary mechanism of resistance was described to be a mutation in the rv0678 gene, a gene that encodes a transcriptional repressor for the efflux pump MmpL5 [125].

2.1.4 Clarithromycin

Clarithromycin is a macrolide antibiotic which differs from erythromycin through substitution of the hydroxy group at the lactone ring by an O-methyl group. This confers greater acid stability resulting in better oral availability and may contribute to improved intracellular activity. Clarithromycin is metabolized by hepatic cytochrome P450 enzymes. Its metabolite 14-hydroxy-clarithromycin has activity with additive or synergistic activity with clarithromycin. Macrolides inhibit protein synthesis by reversible binding to the 50S ribosomal subunit of susceptible bacteria [126] and have immunomodulatory effects [127]. The potential for drug-drug interactions is based on binding and inhibition of macrolides to cytochrome CYP3A4 isoforms. Clarithromycin has less affinity to CYP3A4 than erythromycin [128, 129]

2.1.5 Moxifloxacin

Moxifloxacin is a fluoroquinolone antibiotic, approved for human use by the US FDA in 1999 (https://www.accessdata.fda.gov/drugsatfda_docs/nda/99/21-085_Avelox.cfm). Compared to fluoroquinolones available at that time it has enhanced activity against Gram-positive and atypical bacteria with a comparable spectrum of activity against Gram-negative bacteria. Fluoroquinolones inhibit DNA replication by affecting DNA gyrase and topoisomerase IV with topoisomerase IV being the primary target in Gram-positive bacteria and DNA gyrase that in Gram-negative bacteria. Data obtained in *S. pneumoniae* and *E. coli* suggest moxifloxacin may have equal and simultaneous activity on both enzymes. Fluoroquinolone resistance is based on alteration of the genes coding for the target enzymes and the gene coding for the efflux pump [130].

2.1.6 Tetracycline

Tetracycline is an antibiotic that was discovered in 1953. The term has also been used to describe the structurally related family of antibiotics, which inhibit the bacterial protein biosynthesis by preventing the attachment of the aminoacyl-tRNA to the ribosomal acceptor site. They are broad-spectrum antibiotics, exhibiting activity against a wide range of Gram-positive and Gram-negative bacteria, as well as atypical pathogens like *Chlamydia* [131]. Before the mid-1950s, pathogens resistant to tetracyclines were rare, but since then, many different tetracycline-resistant genes have been characterized. [131].

2.2 Anti-trypanosomal drugs

2.2.1 Fexinidazole

Fexinidazole is 2-substituted 5-nitroimidazole synthesized in the 1970s by Hoechst AG (now Sanofi). Its anti-trypanosomal activity was initially identified in the 1980s and later confirmed [132, 133]. Fexinidazole is metabolized rapidly to sulfoxide and sulfone derivatives [132, 134]. The subsequent metabolism and mechanism of action in trypanosomes are unknown [135].

In 2005, DNDi initiated development for human African trypanosomiasis (HAT). After completion of preclinical studies and an agreement between DNDi and Sanofi for joint development, fexinidazole entered clinical trials in 2009 and obtained an EMA positive ‘scientific opinion’ in 2018 through the Article 58 of Regulation (EC) No 726/2004 procedure in the context of cooperation with WHO (now referred to as EU-M4all) for treatment of first and second stage *Trypanosoma brucei gambiense* HAT in adults and children ≥6 years and weighing ≥20 kg. Through the EU-M4all procedure, the EMA assesses drugs not intended for marketing in the European Union according to the same criteria used for drugs for the European market. The EMA scientific opinion facilitated regulatory approval in *T. b. gambiense* endemic countries [135, 136, 137]. In 2019 fexinidazole was added to the WHO EML and included in the ‘WHO interim guidelines for the treatment of gambiense human African trypanosomiasis’ [138]. In 2020, fexinidazole received US FDA approval [139]. In contrast to all other available treatments for *T.b. gambiense* HAT, fexinidazole is an oral treatment.

A Phase 2/3 study to evaluate the safety and efficacy of fexinidazole for first and second stage *T. b. rhodesiense* HAT (https://clinicaltrials.gov/ct2/show/record/NCT03974178) is expected to be completed in mid 2022. The trial is co-funded by the European & Developing Countries Clinical Trials Partnership (EDCTP) and the Portuguese Fundação para a Ciência e a Tecnologia (https://dndi.org/research-development/portfolio/fexinidazole-tb-rhodesiense/, accessed March 3 2022).

2.2.2 Pentamidine

Pentamidine, a synthetic aromatic diamidine, was introduced in 1940 [140]. The exact target and mode of action is unknown, but may include DNA binding/damage, loss of kinetoplast DNA, and disruption of mitochondrial membrane potential [141, 142]. Loss of kinetoplast DNA has been suggested to precede the loss of mitochondrial membrane potential, but it is unclear if this effect is strictly sequential or if pentamidine also has direct effects on the mitochondrial membrane [141]. Despite 80 years of use, it is still highly effective (cure rate = 93-98%) in treating first-stage *T. b. gambiense* HAT [140, 143], but today is the 1st line treatment only for children <6 years or <20 kg for whom fexinidazole is not yet registered. Pentamidine is administered once daily intramuscularly for 7-10 days [140]. If given intravenously, care must be taken that it is not given as a bolus, but rather slowly over 60 minutes to avoid a possible induction of hypoglycemia. An oral analogue of pentamidine had comparable efficacy to the injected form, but was too toxic [144]. As there is little economic incentive in pentamidine, its production was almost discontinued, but in 2001, Sanofi-Aventis agreed to continue producing it for the WHO [143]. It cannot pass the blood-brain barrier and therefore is not effective for second-stage infections [140].

2.2.3 Suramin

Suramin is a polysulfonated napththyl urea introduced to treat *T. b. gambiense* and *T. brucei rhodesiense* HAT in 1922 and is one of the first anti-infective agents developed from trypan blue and trypane red in one of the first medicinal chemistry programs at Bayer [140, 145]. Due to its six negative charges at physiological pH, it does not pass the blood brain barrier and, therefore, was only effective against first-stage trypanosomiasis disease [145]. Suramin is a multifunctional compound with activity against other parasitic diseases, viruses, cancers, and snakebites, and even autism [145]. Due to the many targets, the mode of action of suramin is not well understood in general and even less-so in trypanosomes [145]. In trypanosomes it has been shown to inhibit cytokinesis as demonstrated by cells with two nuclei [141]. Suramin has been shown to inhibit glycolytic enzymes and inhibit oxidative phosphorylation, although it is not understood how the large and highly negatively charged

molecule can pass the membranes of the glycosomes and mitochondria where the enzymes are located [145]. Life threatening reactions to the 7-day course for *T. b. rhodesiense* HAT and lethal outcomes are rare. Pyrexia and usually mild and reversible nephrotoxicity are driven by concentrations in the kidneys [140]. As *T. b. rhodesiense* HAT progresses quickly to the second stage, treatment of pregnant women with first-stage HAT cannot be delayed until after birth of the child [140].

Suramin administered for 6 weeks is macrofilaricidal in *O. volvulus*, but the adverse reactions make it unsuitable for large scale use [146, 147]. Recent research shows activity against *Leishmania major* and *L. donovani*, and that suramin can block host cell invasion by *Plasmodium falciparum* [145]. Suramin is instable in air and must be administered for HAT by slow intravenous injection every 3-7 days for 4 weeks [140, 144].

2.2.4 Melarsoprol

Melarsoprol is a trivalent organic arsenical compound that, since 1949, was the first-line drug of choice over other arsenic derivatives to treat second-stage *T.b. gambiense* and *T. b. rhodesiense* HAT [140, 143, 144]. The mode of action of melarsoprol is still being elucidated. It forms adducts with trypanothione and is an indiscriminate inhibitor of kinases (mainly dithiol containing trypanosomal enzymes); the latter indicating involvement of signaling cascades [140, 141]. Treatment of trypanosomes results in a defect in mitosis shown by an increased number of cells with replicated but unsegregated nuclear genomes [141]. The compound is liposoluble and administered intravenously in propylene glycol, an irritant. This makes melarsoprol not only difficult to administer, requiring hospitalization, but painful to the patients [143]. For several decades, melarsoprol treatment followed the regimens of other arsenicals and varied from country to country, including serial drug application with 1-week intervals without drug [140, 148]. Pharmacokinetic studies supported the hypothesis that a shorter, uninterrupted treatment regimen could be equally effective [149, 150]. This informed studies which showed the safety and efficacy of a 10-day treatment for second stage *T. b. gambiense* HAT [149, 150, 151] and *T. b. rhodesiense* HAT [148]. The most feared adverse reaction is reactive encephalopathy that can occur in up to 10% of patients with a median fatality rate of 50% [140, 143]. Melarsoprol is still the only drug available to treat second-stage *T. b. rhodesiense* HAT [140] but is today only a rescue treatment for *T. b. gambiense* HAT patients who have failed treatment with Nifurtimox-Eflornithine Combination Treatment (NECT) and fexinidazole [138]. There are few data on the safety of melarsoprol during pregnancy, but theoretically it is contraindicated. However, due to the severity of *T. b. rhodesiense* HAT, its use as treatment cannot be delayed until after the birth of the child [140].

2.2.5 Eflornithine

Eflornithine (D,L-α-difluoromethly ornithine) was at one time evaluated as a cancer drug. It's antitrypanosomal effect was discovered in 1980 through WHO/TDR funded studies on the polyamine metabolism of trypanosomes [152]. Eflornithine irreversibly inhibits ornithine decarboxylase of trypanosomes [143], blocking production of the polyamine putrescine and subsequent DNA synthesis. This results in the parasites entering a dormant state susceptible to the host immune system [140]. Trypanosomes are more sensitive to the drug than mammalian cells, probably due to a slower ornithine decarboxylase turnover rate [144].

Eflornithine's effect on patients with second stage *T. b. gambiense* HAT [153] earned it the name 'resurrection drug'. Despite that, and the fact that public funding provided by WHO/TDR contributed to eflornithine development [153, 154], eflornithine was no longer available after the manufacturer Marion Merrell Dow merged with Hoechst and Roussel. WHO was not able to identify an alternative manufacturer for an affordable price [152, 155].

It took the discovery that eflornithine was being manufactured for a cream to remove unwanted facial hair in women, which had received regulatory approval in 2000 [156], and subsequent intense advocacy by Médecins Sans Frontières and WHO for eflornithine to become available again to save the lives of patients with HAT [152, 157, 158]. Currently, supply until 2025 has been secured through renewal of the WHO and Sanofi agreement [159]. Eflornithine is not used to treat second-stage *T. b. rhodesiense* HAT as these parasites are less susceptible to the compound [143]. Although an oral formulation was available, it did not ensure high enough levels in the cerebral spinal fluid, and eflornithine is therefore given intravenously [140]. As monotherapy, it requires administration 4 times a day every six hours for 14 days [144], which is difficult in the health centers in the remote rural areas where HAT is endemic. Through combination with 10 days of oral nifurtimox treatment every 8 hours (NECT), eflornithine eflornithine infusion can be reduced to twice daily for 7-days [160, 161]. Little information is available on the safety of eflornithine during pregnancy and, when possible, watchful waiting (regular monthly clinical assessment) is used. If the health of the mother is at risk, eflornithine or NECT is given, otherwise pentamidine is used after the first trimester to prevent infection of the fetus [138, 140].

2.2.6 Benznidazole

Benznidazole is a nitroimidazole antiparasitic drug for treatment of acute and early chronic Chagas disease [7]. It is reduced to reactive metabolites by nitroreductases, but the mechanism of its action is not well understood [162, 163, 164]. Despite availability since 1971, its role in the treatment of chronic disease remains under discussion [165, 166]. Its better tolerance in infants and children than adults has been attributed to a faster hepatic elimination compared to adults [167]. A paediatric formulation was developed and registered in Brazil in 2011, in the US in 2017 (for ages 2-12 years https://www.accessdata.fda.gov/drugsatfda_docs/nda/2017/209570Orig1s000TOC.cfm, accessed 20 June 2022) , and in Argentina in 2018 [168]. A recent study concluded that shorter treatment durations and/or lower doses of benznidazole could have similar antiparasitic effects with better tolerability than the current standard treatment, a promising finding to be confirmed via further studies [169, 170].

2.2.7 Nifurtimox

Nifurtimox is a 5-nitrofuran derivative, introduced for treatment of Chagas disease in the late 1960ies [171]. Following demonstration of the efficacy and safety of NECT, the combination of oral nifurtimox with intravenous eflornithine, for treatment of second stage *T. b. gambiense* HAT [160, 161], this combination was added to the WHO EML in 2009 [172] and became the treatment of choice for this indication [158, 173]. Nifurtimox was added to the WHO EML for children for African trypanosomiasis in 2013 [174] after data became available demonstrating that in children NECT efficacy is comparable and safety comparable or better in children than adults [173, 175, 176, 177]. The mechanism of action for both *T. brucei* and *T. cruzi* involves reduction by an NADH-dependent bacterial-like nitroreductase and generation of a cytotoxic, unsaturated open-chain nitrile derivative [133].

2.3 Antileishmanial drugs

2.3.1 Pentavalent antimoniate

The first use of a pentavalent antimonial (urea stibamine) for leishmaniasis dates back to 1922 [178]. Pentavalent antimony (Sb(V)) complexes currently in use include N-methyl-D-glucamine (meglumine antimoniate, Glucantime®, 100 mg Sb/mL) and sodium gluconate (sodium stibogluconate, Pentostam®, 85 mg Sb^{+}/mL) [179, 180]. Pentavalent antimonials may be prodrugs converted to the active trivalent antimony, act directly, and/or via

stimulation of the immune system [Frezard et al. 2009] [Haldar et al. 2011]. As for other antileishmanial drugs, efficacy and role of pentavalent antimoniates in case management depends on clinical form, pathology, causative species, geographic region, use history, and co-infections [178, 181, 182, 183].

2.3.2 Amphotericin B and liposomal amphotericin B

Amphotericin B is a polyene antibiotic with efficacy against fungal infections as well as *T. cruzi*, *Schistosoma mansoni*, *Echinococcus multilocularis* and *Leishmania spp* [184]. The long-established notion that Amphotericin B mechanism of action is via cell membrane pore formation after binding to ergosterol has been questioned in favour of pleiotropic effects including induction of oxidative damage and an immunomodulatory effect [184]. It is available in several formulations: deoxycholate solution, colloidal dispersion with cholesterol sulphate, a lipid complex with two phospholipids, and in unilamellar liposomes formed from cholesterol and other phospholipids [178, 184, 185].

Liposomal amphotericin B was originally developed for severe systemic and deep mycoses. It was first used to treat a European with mediterranean visceral leishmaniasis who had failed treatment with antimonials, pentamidine and paromomycin [186, 187]. This and subsequent experience motivated a clinical development programme to obtain the data needed for registration of liposomal amphotericin B for treatment of visceral leishmaniasis in collaboration between the company and WHO/TDR [186]. Liposomal amphotericin B (Ambisome) was approved by the US FDA in 1997 for three indications including visceral leishmaniasis (https://www.accessdata.fda.gov/drugsatfda_docs/nda/97/050740_ambisome_toc.cfm). A single dose of liposomal amphotericin B has been shown to be efficacious as well as safe and effective for visceral leishmaniasis in India and Bangladesh [188, 189, 190] and has become the preferred first-line treatment, replacing miltefosine [191]. Further studies are needed to define the optimum treatment liposomal amphotericin B regimen for other clinical presentations, pathology, causative species, geographic region, use history and co-infections. [192].

2.3.3 Miltefosine

Miltefosine (hexadecylphosphocholine) is an alkyl phosphocholine compound and a structural analogue of lecithin [193]. It was evaluated from the 1980ies as a cancer drug resulting in registration of a topical formulation for treatment of skin lesion from breast cancer [194, 195]. Research into miltefosine's antileishmanial effect also dates back to the 1980ies [196].

Mechanisms involved in its antileishmanial action include incorporation into membrane lipid bilayers, disturbance of membrane metabolism and induction of apoptosis-like cell death as well as host-mediated immunomodulation [193].

Dose limiting gastrointestinal adverse reactions during long treatment resulted in discontinuation of development of oral formulations for cancer. The pre-clinical as well as clinical data obtained during that development [194] provided a valuable basis for development of miltefosine as the first oral treatment for visceral leishmaniasis [197]. Miltefosine is teratogenic in rats, but not rabbits. Contraindication during pregnancy is one of its main limitations [194, 198]. The availability of an oral treatment was one of the factors that lead to a regional strategic framework for elimination of visceral leishmaniasis from the Indian Subcontinent [199, 200].

Miltefosine development for treatment of visceral leishmaniasis was accomplished in collaboration between Asta Medica (Zentaris, Germany) and WHO/TDR with WHO/TDR providing both relevant expertise as well as funding. The collaboration was based on a

Memorandum of Understanding concluded in 1995 between WHO and the company which included provisions for availability and affordability of miltefosine for the public health systems in endemic countries, should development be successful and result in registration [195, 201]. Registration was achieved initially in Germany and in India in 2002 and later in other endemic countries. However, affordable access was only temporary. In contrast, the incentives provided by the US congress for investment into the development of drugs for neglected diseases via the 'Priority Review Voucher' (https://www.fda.gov/about-fda/center-drug-evaluation-and-research-cder/tropical-disease-priority-review-voucher-program) resulted in a company (Knight Therapeutics) who had never invested in miltefosine development, nor committed to making miltefosine available at affordable prices, benefitting from these incentives to the tune of US$125 Million [195, 202]. This has resulted in calls to change the conditions under which a 'Priority Review Voucher' is awarded [202, 203].

2.3.4 Paromomycin

Paromomycin, a highly hydrophilic and lipid insoluble aminoglycoside also known as aminosidine, is a broad-spectrum antibiotic . Its antileishmanial effect was discovered in 1960 [180]. The mechanism of action of paromomycin is inhibition of protozoan protein synthesis by binding to the 30S ribosomal subunit resulting in the accumulation of abnormal 30S–50S ribosomal complexes and finally causing cell death [180]. Paromomycin can be used in pregnant leishmaniasis patients [180].

Starting in 1993, WHO/TDR supported studies of topical paromomycin treatment for cutaneous leishmaniasis **[204, 205, 206]** as well as studies for qualifying injectable formulations for treatment of visceral leishmaniasis (including mutagenicity and genotoxicity studies, a study on the bioequivalence of two injectable formulations and a multi-centre study comparing the efficacy and safety of injectable paromomycin with that of amphotericin B **[154, 207, 208]**. Lack of funding resulted in development being put on hold after completion of a Phase II study in 2000 and was resumed in 2001-2002 in partnership with the Institute of One World Health had received a grant from the Bill and Melinda Gates Foundation (https://www.gatesfoundation.org/ideas/media-center/press-releases/2002/08/institute-for-oneworld-health-receives-grant, accessed 28 June 2022) **[209, 210]**. Paromomycin was registered for visceral leishmaniasis in 2006 **[178]** Paromomycin can be used in pregnant leishmaniasis patients **[180]**.

2.4 Antifungal drugs

2.4.1 Itraconazole

Itraconazole is a broad-spectrum antifungal azole drug, patented in 1978 and approved by the US FDA in 1992 (https://www.accessdata.fda.gov/scripts/cder/daf/index.cfm?event=overview.process&ApplNo=020083, accessed 28 June 2022). Azoles inhibit the synthesis of ergosterol via the inhibition of lanosterol 14α-demethylase resulting in fungal membrane destruction. Itraconazole also impacts several metabolic pathways important for human cell proliferation resulting in interest in repurposing itraconazole for cancer therapy [211] Itraconazole, like other azoles, is metabolized by cytochome P450 3A4. Therefore, drug-drug interactions with other drugs metabolized by CYP450 3A4 need to be considered [212].

3 References

1. World Health Organization. The selection and use of essential medicines: Report of the WHO Expert Committee on Selection and Use of Essential Medicines, 2021 (including the 22nd WHO model list of essential medicines and the 8th WHO model

list of essential medicines for children). 2021. https://apps.who.int/iris/handle/10665/351172.

2. Barda B, Sayasone S, Phongluxa K, Xayavong S, Keoduangsy K, Odermatt P, et al. Efficacy of moxidectin versus ivermectin against *Strongyloides stercoralis* infections: A randomized, controlled noninferiority trial. Clin Infect Dis. 2017;65 2:276-81; doi: 10.1093/cid/cix278. https://www.ncbi.nlm.nih.gov/pubmed/28369530.
3. Barda B, Ame SM, Ali SM, Albonico M, Puchkov M, Huwyler J, et al. Efficacy and tolerability of moxidectin alone and in co-administration with albendazole and tribendimidine versus albendazole plus oxantel pamoate against *Trichuris trichiura* infections: a randomised, non-inferiority, single-blind trial. Lancet Infect Dis. 2018;18 8:864-73; doi: 10.1016/S1473-3099(18)30233-0. https://www.ncbi.nlm.nih.gov/pubmed/29858149.
4. Keller L, Palmeirim MS, Ame SM, Ali SM, Puchkov M, Huwyler J, et al. Efficacy and safety of ascending dosages of moxidectin and moxidectin-albendazole against *Trichuris trichiura* in adolescents: A randomized controlled trial. Clin Infect Dis. 2020;70 6:1193-201; doi: 10.1093/cid/ciz326. https://www.ncbi.nlm.nih.gov/pubmed/31044235.
5. Hofmann D, Sayasone S, Sengngam K, Chongvilay B, Hattendorf J, Keiser J. Efficacy and safety of ascending doses of moxidectin against *Strongyloides stercoralis* infections in adults: a randomised, parallel-group, single-blinded, placebo-controlled, dose-ranging, phase 2a trial. Lancet Infect Dis. 2021;in press; doi: 10.1016/S1473-3099(20)30691-5. https://www.ncbi.nlm.nih.gov/pubmed/33798487.
6. Barda B, Coulibaly JT, Puchkov M, Huwyler J, Hattendorf J, Keiser J. Efficacy and safety of moxidectin, synriam, synriam-praziquantel versus praziquantel against *Schistosoma haematobium* and *S. mansoni* infections: A randomized, exploratory phase 2 trial. PLoS Negl Trop Dis. 2016;10 9:e0005008; doi: 10.1371/journal.pntd.0005008. https://www.ncbi.nlm.nih.gov/pubmed/27636542.
7. World Health Organization. Ending the neglect to attain the Sustainable Development Goals – A road map for neglected tropical diseases 2021–2030. 2020:177. https://www.who.int/neglected_diseases/WHONTD-roadmap-2030/en/.
8. Kuesel AC. Research for new drugs for elimination of onchocerciasis in Africa. Int J Parasitol Drugs Drug Resist. 2016;6 3:272-86; doi: 10.1016/j.ijpddr.2016.04.002. https://www.ncbi.nlm.nih.gov/pubmed/27693536.
9. World Health Organization. Road map for neglected tropical diseases 2021–2030. 2020;73 A73/8:1. https://apps.who.int/gb/ebwha/pdf_files/WHA73/A73(33)-en.pdf.
10. World Health Organization. Second meeting of the WHO diagnostic technical advisory group for neglected tropical diseases, 13 October 2020. 2021. https://apps.who.int/iris/handle/10665/341389.
11. Campbell WCBRWF, M. H.; Dybas, R.A. The discovery of ivermectins and other avermectins. 1984;255.
12. Abongwa M, Martin RJ, Robertson AP. A Brief review on the mode of action of antinematodal drugs. Acta Vet (Beogr). 2017;67 2:137-52; doi: 10.1515/acve-2017-0013. https://www.ncbi.nlm.nih.gov/pubmed/29416226.

13. Ottesen EA, Campbell WC. Ivermectin in human medicine. J Antimicrob Chemother. 1994;34 2:195-203; doi: 10.1093/jac/34.2.195. https://www.ncbi.nlm.nih.gov/pubmed/7814280.
14. Prichard R, Menez C, Lespine A. Moxidectin and the avermectins: Consanguinity but not identity. Int J Parasitol Drugs Drug Resist. 2012;2:134-53; doi: 10.1016/j.ijpddr.2012.04.001. https://www.ncbi.nlm.nih.gov/pubmed/24533275.
15. Ali MM, Mukhtar MM, Baraka OZ, Homeida MM, Kheir MM, Mackenzie CD. Immunocompetence may be important in the effectiveness of Mectizan (ivermectin) in the treatment of human onchocerciasis. Acta Trop. 2002;84 1:49-53; doi: 10.1016/s0001-706x(02)00117-1. https://www.ncbi.nlm.nih.gov/pubmed/12387910.
16. Reaves BJ, Wallis C, McCoy CJ, Lorenz WW, Rada B, Wolstenholme AJ. Recognition and killing of *Brugia malayi* microfilariae by human immune cells is dependent on the parasite sample and is not altered by ivermectin treatment. Int J Parasitol Drugs Drug Resist. 2018;8 3:587-95; doi: 10.1016/j.ijpddr.2018.09.002. https://www.ncbi.nlm.nih.gov/pubmed/30279092.
17. Wilson NE, Reaves BJ, Wolstenholme AJ. Lack of detectable short-term effects of a single dose of ivermectin on the human immune system. Parasit Vectors. 2021;14 1:304; doi: 10.1186/s13071-021-04810-6. https://www.ncbi.nlm.nih.gov/pubmed/34090504.
18. Wolstenholme AJ, Maclean MJ, Coates R, McCoy CJ, Reaves BJ. How do the macrocyclic lactones kill filarial nematode larvae? Invert Neurosci. 2016;16 3:7; doi: 10.1007/s10158-016-0190-7. https://www.ncbi.nlm.nih.gov/pubmed/27279086.
19. Fujisaki T, Reich MR, Research UNWBWSPf, Training in Tropical D: TDR's contribution to the development of ivermectin for onchocerciasis : a report / prepared by Tomoko Fujisaki and Michael Reich. Geneva: World Health Organization; 1998.
20. UNICEF/UNDP/World Bank/WHO Special Programme for Research and Training in Tropical Diseases (TDR). Making a difference. 30 Years of research and capacity building in tropical diseases. 2007.
21. Sutherland IH. Veterinary use of ivermectin. Acta Leiden. 1990;59 1-2:211-6. https://www.ncbi.nlm.nih.gov/pubmed/2198752.
22. Sutherland IH, Campbell WC. Development, pharmacokinetics and mode of action of ivermectin. Acta Leiden. 1990;59 1-2:161-8. https://www.ncbi.nlm.nih.gov/pubmed/2378205.
23. Boussinesq M, Gardon J, Gardon-Wendel N, Chippaux JP. Clinical picture, epidemiology and outcome of *Loa*-associated serious adverse events related to mass ivermectin treatment of onchocerciasis in Cameroon. Filaria J. 2003;2 Suppl 1:S4; doi: 10.1186/1475-2883-2-S1-S4. http://www.ncbi.nlm.nih.gov/pubmed/14975061.
24. Boussinesq M, Kamgno J, Pion SD, Gardon J. What are the mechanisms associated with post-ivermectin serious adverse events? Trends Parasitol. 2006;22 6:244-6; doi: 10.1016/j.pt.2006.04.006. https://www.ncbi.nlm.nih.gov/pubmed/16632406.
25. Gardon J, Gardon-Wendel N, Demanga N, Kamgno J, Chippaux JP, Boussinesq M. Serious reactions after mass treatment of onchocerciasis with ivermectin in an area endemic for *Loa loa* infection. Lancet. 1997;350 9070:18-22. 9217715

26. Mectizan Expert Committee and APOC Technical Consultative Committee: Recommendations for the treatment of onchocerciasis with Mectizan in areas co-endemic for onchocerciasis and loiasis. https://mectizan.org/wp-content/uploads/2018/06/englishmectccloarecs-june04.pdf (2004). Accessed 7/11/2022 2022.
27. Boussinesq M, Fobi G, Kuesel AC. Alternative treatment strategies to accelerate the elimination of onchocerciasis. Int Health. 2018;10 suppl_1:i40-i8; doi: 10.1093/inthealth/ihx054. https://www.ncbi.nlm.nih.gov/pubmed/29471342.
28. Awadzi K, Attah SK, Addy ET, Opoku NO, Quartey BT, Lazdins-Helds JK, et al. Thirty-month follow-up of sub-optimal responders to multiple treatments with ivermectin, in two onchocerciasis-endemic foci in Ghana. Ann Trop Med Parasitol. 2004;98 4:359-70. 15228717
29. Awadzi K, Boakye DA, Edwards G, Opoku NO, Attah SK, Osei-Atweneboana MY, et al. An investigation of persistent microfilaridermias despite multiple treatments with ivermectin, in two onchocerciasis-endemic foci in Ghana. Ann Trop Med Parasitol. 2004;98 3:231-49. 15119969
30. Frempong K, Walker M, Cheke R, Tettevi E, Gyan E, Owusu E, et al. Does increasing treatment frequency address sub-optimal responses to ivermectin for the control and elimination of River Blindness? Clin Inf Dis. 2016;in press.
31. Osei-Atweneboana MY, Awadzi K, Attah SK, Boakye DA, Gyapong JO, Prichard RK. Phenotypic evidence of emerging ivermectin resistance in *Onchocerca volvulus*. PLoS Negl Trop Dis. 2011;5 3:e998; doi: 10.1371/journal.pntd.0000998. http://www.ncbi.nlm.nih.gov/pubmed/21468315.
32. Osei-Atweneboana MY, Eng JK, Boakye DA, Gyapong JO, Prichard RK. Prevalence and intensity of *Onchocerca volvulus* infection and efficacy of ivermectin in endemic communities in Ghana: a two-phase epidemiological study. Lancet. 2007;369 9578:2021-9. http://www.ncbi.nlm.nih.gov/entrez/query.fcgi?cmd=Retrieve&db=PubMed&dopt=Citation&list_uids=17574093
33. Abong RA, Amambo GN, Chounna Ndongmo PW, Njouendou AJ, Ritter M, Beng AA, et al. Differential susceptibility of *Onchocerca volvulus* microfilaria to ivermectin in two areas of contrasting history of mass drug administration in Cameroon: relevance of microscopy and molecular techniques for the monitoring of skin microfilarial repopulation within six months of direct observed treatment. BMC Infect Dis. 2020;20 1:726; doi: 10.1186/s12879-020-05444-2. https://www.ncbi.nlm.nih.gov/pubmed/33008333.
34. Doyle SR, Bourguinat C, Nana-Djeunga HC, Kengne-Ouafo JA, Pion SDS, Bopda J, et al. Genome-wide analysis of ivermectin response by *Onchocerca volvulus* reveals that genetic drift and soft selective sweeps contribute to loss of drug sensitivity. PLoS Negl Trop Dis. 2017;11 7:e0005816; doi: 10.1371/journal.pntd.0005816. https://www.ncbi.nlm.nih.gov/pubmed/28746337.
35. Bakajika D, Kanza EM, Opoku NO, Howard HM, Mambandu GL, Nyathirombo A, et al. Effect of a single dose of 8 mg moxidectin or 150 mug/kg ivermectin on *O. volvulus* skin microfilariae in a randomized trial: Differences between areas in the Democratic Republic of the Congo, Liberia and Ghana and impact of intensity of infection. PLoS

Negl Trop Dis. 2022;16 4:e0010079; doi: 10.1371/journal.pntd.0010079 [doi];PNTD-D-21-01732 [pii]. http://www.ncbi.nlm.nih.gov/pubmed/35476631.
36. Opoku NO, Bakajika DK, Kanza EM, Howard H, Mambandu GL, Nyathirombo A, et al. Single dose moxidectin versus ivermectin for *Onchocerca volvulus* infection in Ghana, Liberia, and the Democratic Republic of the Congo: a randomised, controlled, double-blind phase 3 trial. Lancet. 2018;392 10154:1207-16; doi: 10.1016/S0140-6736(17)32844-1. https://www.ncbi.nlm.nih.gov/pubmed/29361335.
37. Horton J. Albendazole: a review of anthelmintic efficacy and safety in humans. Parasitology. 2000;121 Suppl:S113-32. http://www.ncbi.nlm.nih.gov/entrez/query.fcgi?cmd=Retrieve&db=PubMed&dopt=Citation&list_uids=11386684
38. Dayan AD. Albendazole, mebendazole and praziquantel. Review of non-clinical toxicity and pharmacokinetics. Acta Trop. 2003;86 2-3:141-59; doi: 10.1016/s0001-706x(03)00031-7. https://www.ncbi.nlm.nih.gov/pubmed/12745134.
39. Van den Bossche H, Rochette F, Horig C. Mebendazole and related anthelmintics. Adv Pharmacol Chemother. 1982;19:67-128; doi: 10.1016/s1054-3589(08)60021-6. https://www.ncbi.nlm.nih.gov/pubmed/6762073.
40. US FDA Center for Drug Evaluation and Research. Approval package for Egaten (triclabendazole) 250 mg tablets. 2019; doi: https://www.accessdata.fda.gov/drugsatfda_docs/appletter/2019/208711Orig1s000ltr.pdf. https://www.accessdata.fda.gov/drugsatfda_docs/nda/2018/208711Orig1s000TOC.cfm.
41. Gandhi P, Schmitt EK, Chen CW, Samantray S, Venishetty VK, Hughes D. Triclabendazole in the treatment of human fascioliasis: a review. Trans R Soc Trop Med Hyg. 2019;113 12:797-804; doi: 10.1093/trstmh/trz093. https://www.ncbi.nlm.nih.gov/pubmed/31638149.
42. Pi H, Ogunniyi AD, Savaliya B, Nguyen HT, Page SW, Lacey E, et al. Repurposing of the fasciolicide triclabendazole to treat infections caused by *Staphylococcus* spp. and vancomycin-resistant Enterococci. Microorganisms. 2021;9 8; doi: 10.3390/microorganisms9081697. https://www.ncbi.nlm.nih.gov/pubmed/34442776.
43. Higazi T, Geary T, Mackenzie CD. Chemotherapy in the treatment, control and elimination of human onchocerciasis. Res Rep Trop Med. 2014;5:77-93; doi: 10.2147/RRTM.S3664. https://www.ncbi.nlm.nih.gov/pmc/articles/PMC7337210/pdf/RRTM-5-77.pdf.
44. Florencio MS, Peixoto CA. The effects of diethylcarbamazine on the ultrastructure of microfilariae of *Wuchereria bancrofti*. Parasitology. 2003;126 Pt 6:551-4. https://www.ncbi.nlm.nih.gov/pubmed/12866792.
45. Weaver A, Brown P, Huey S, Magallon M, Bollman EB, Mares D, et al. A low-tech analytical method for diethylcarbamazine citrate in medicated salt. PLoS Negl Trop Dis. 2011;5 2:e1005; doi: 10.1371/journal.pntd.0001005. https://www.ncbi.nlm.nih.gov/pubmed/21347443.
46. World Health Organization. Global programme to eliminate lymphatic filariasis: Progress report, 2020. Wkly Epidemiol Rec. 2021;96 41:497-508; doi: doi:

10.1093/cid/ciw835. https://www.who.int/publications/i/item/who-wer9641-497-508.
47. Adinarayanan S, Critchley J, Das PK, Gelband H. Diethylcarbamazine (DEC)-medicated salt for community-based control of lymphatic filariasis. Cochrane Database Syst Rev. 2007; 1:CD003758; doi: 10.1002/14651858.CD003758.pub2. https://www.ncbi.nlm.nih.gov/pubmed/17253495.
48. Gelband H. Diethylcarbamazine salt in the control of lymphatic filariasis. Am J Trop Med Hyg. 1994;50 6:655-62; doi: 10.4269/ajtmh.1994.50.655. https://www.ncbi.nlm.nih.gov/pubmed/8024057.
49. Lammie P, Milner T, Houston R. Unfulfilled potential: using diethylcarbamazine-fortified salt to eliminate lymphatic filariasis. Bull World Health Organ. 2007;85 7:545-9. http://dx.doi.org/10.2471/BLT.06.034108.
50. Fang Y, Zhang Y. Lessons from lymphatic filariasis elimination and the challenges of post-elimination surveillance in China. Infect Dis Poverty. 2019;8 1:66; doi: 10.1186/s40249-019-0578-9. https://www.ncbi.nlm.nih.gov/pubmed/31387644.
51. Sharma S, Smith ME, Reimer J, O'Brien DB, Brissau JM, Donahue MC, et al. Economic performance and cost-effectiveness of using a DEC-salt social enterprise for eliminating the major neglected tropical disease, lymphatic filariasis. PLoS Negl Trop Dis. 2019;13 7:e0007094; doi: 10.1371/journal.pntd.0007094. https://www.ncbi.nlm.nih.gov/pubmed/31260444.
52. Panicker KN, Arunachalam N, Kumar NP, Prathibha J, Sabesan S. Efficacy of diethylcarbamazine-medicated salt for microfilaraemia of *Brugia malayi*. Natl Med J India. 1997;10 6:275-6. https://www.ncbi.nlm.nih.gov/pubmed/9481098.
53. Ramaiah KD, Thiruvengadam B, Vanamail P, Subramanian S, Gunasekaran S, Nilamani N, et al. Prolonged persistence of residual *Wuchereria bancrofti* infection after cessation of diethylcarbamazine-fortified salt programme. Journal Article workform. 2009;14 8:870-6; doi: 10.1111/j.1365-3156.2009.02307.x. https://www.ncbi.nlm.nih.gov/pubmed/19552662.
54. Shriram AN, Krishnamoorthy K, Vijayachari P. Diurnally subperiodic filariasis among the Nicobarese of Nicobar district - epidemiology, vector dynamics & prospects of elimination. Indian J Med Res. 2015;141 5:598-607; doi: 10.4103/0971-5916.159537. https://www.ncbi.nlm.nih.gov/pubmed/26139777.
55. Chai JY. Praziquantel treatment in trematode and cestode infections: an update. Infect Chemother. 2013;45 1:32-43; doi: 10.3947/ic.2013.45.1.32 [doi]. http://www.ncbi.nlm.nih.gov/pubmed/24265948.
56. El Ridi RA, Tallima HA. Novel therapeutic and prevention approaches for schistosomiasis: review. J Adv Res. 2013;4 5:467-78; doi: 10.1016/j.jare.2012.05.002 [doi];S2090-1232(12)00031-8 [pii]. http://www.ncbi.nlm.nih.gov/pubmed/25685454.
57. McManus DP, Dunne DW, Sacko M, Utzinger J, Vennervald BJ, Zhou XN. Schistosomiasis. Nat Rev Dis Primers. 2018;4 1:13; doi: 10.1038/s41572-018-0013-8 [doi];10.1038/s41572-018-0013-8 [pii]. http://www.ncbi.nlm.nih.gov/pubmed/30093684.
58. Siqueira LDP, Fontes DAF, Aguilera CSB, Timoteo TRR, Angelos MA, Silva LCPB, et al. Schistosomiasis: Drugs used and treatment strategies. Acta Trop. 2017;176:179-87;

doi: S0001-706X(17)30681-2 [pii];10.1016/j.actatropica.2017.08.002 [doi]. http://www.ncbi.nlm.nih.gov/pubmed/28803725.

59. Vale N, Gouveia MJ, Rinaldi G, Brindley PJ, Gartner F, Correia da Costa JM. Praziquantel for Schistosomiasis: Single-Drug Metabolism Revisited, Mode of Action, and Resistance. Antimicrob Agents Chemother. 2017;61 5; doi: AAC.02582-16 [pii];10.1128/AAC.02582-16 [doi]. http://www.ncbi.nlm.nih.gov/pubmed/28264841.

60. Cioli D, Pica-Mattoccia L, Basso A, Guidi A. Schistosomiasis control: Praziquantel forever? Mol Biochem Parasitol. 2014;195 1:23-9; doi: S0166-6851(14)00075-9 [pii];10.1016/j.molbiopara.2014.06.002 [doi]. http://www.ncbi.nlm.nih.gov/pubmed/24955523.

61. Olliaro PL, Coulibaly JT, Garba A, Halleux C, Keiser J, King CH, et al. Efficacy and safety of single-dose 40 mg/kg oral praziquantel in the treatment of schistosomiasis in preschool-age versus school-age children: An individual participant data meta-analysis. PLoS Negl Trop Dis. 2020;14 6:e0008277; doi: 10.1371/journal.pntd.0008277 [doi];PNTD-D-19-02157 [pii]. http://www.ncbi.nlm.nih.gov/pubmed/32569275.

62. Olliaro PL, Vaillant MT, Belizario VJ, Lwambo NJ, Ouldabdallahi M, Pieri OS, et al. A multicentre randomized controlled trial of the efficacy and safety of single-dose praziquantel at 40 mg/kg vs. 60 mg/kg for treating intestinal schistosomiasis in the Philippines, Mauritania, Tanzania and Brazil. PLoS Negl Trop Dis. 2011;5 6:e1165. http://www.ncbi.nlm.nih.gov/pubmed/21695161.

63. Olliaro P, Delgado-Romero P, Keiser J. The little we know about the pharmacokinetics and pharmacodynamics of praziquantel (racemate and R-enantiomer). J Antimicrob Chemother. 2014;69 4:863-70; doi: dkt491 [pii];10.1093/jac/dkt491 [doi]. http://www.ncbi.nlm.nih.gov/pubmed/24390933.

64. Mahende MK, Huber E, Kourany-Lefoll E, Ali A, Hayward B, Bezuidenhout D, et al. Comparative palatability of orally disintegrating tablets (ODTs) of Praziquantel (L-PZQ and Rac-PZQ) versus current PZQ tablet in African children: A randomized, single-blind, crossover study. PLoS Negl Trop Dis. 2021;15 6:e0007370; doi: 10.1371/journal.pntd.0007370 [doi];PNTD-D-19-00509 [pii]. http://www.ncbi.nlm.nih.gov/pubmed/34106922.

65. Reinhard-Rupp J, Klohe K. Developing a comprehensive response for treatment of children under 6 years of age with schistosomiasis: research and development of a pediatric formulation of praziquantel. Infect Dis Poverty. 2017;6 1:122; doi: 10.1186/s40249-017-0336-9 [doi];10.1186/s40249-017-0336-9 [pii]. http://www.ncbi.nlm.nih.gov/pubmed/28768535.

66. Bulkley D, Innis CA, Blaha G, Steitz TA. Revisiting the structures of several antibiotics bound to the bacterial ribosome. Proc Natl Acad Sci U S A. 2010;107 40:17158-63; doi: 10.1073/pnas.1008685107.

67. Firth A, Prathapan P. Azithromycin: The first broad-spectrum therapeutic. Eur J Med Chem. 2020;207:112739; doi: 10.1016/j.ejmech.2020.112739.

68. Dunay IR, Gajurel K, Dhakal R, Liesenfeld O, Montoya JG. Treatment of toxoplasmosis: Historical perspective, animal models, and current clinical practice. Clin Microbiol Rev. 2018;31 4; doi: 10.1128/cmr.00057-17.

69. Kennedy K, Cobbold SA, Hanssen E, Birnbaum J, Spillman NJ, McHugh E, et al. Delayed death in the malaria parasite *Plasmodium falciparum* is caused by disruption of prenylation-dependent intracellular trafficking. PLoS Biol. 2019;17 7:e3000376; doi: 10.1371/journal.pbio.3000376. https://www.ncbi.nlm.nih.gov/pubmed/31318858.
70. Sidhu ABS, Sun Q, Nkrumah LJ, Dunne MW, Sacchettini JC, Fidock DA. *In vitro* efficacy, resistance selection, and structural modeling studies implicate the malarial parasite apicoplast as the target of azithromycin. J Biol Chem. 2007;282 4:2494-504; doi: https://doi.org/10.1074/jbc.M608615200. https://www.sciencedirect.com/science/article/pii/S0021925820721198.
71. John LN, Bjerum C, Martinez PM, Likia R, Silus L, Wali C, et al. Pharmacokinetic and safety study of co-administration of albendazole, diethylcarbamazine, ivermectin and azithromycin for the integrated treatment of Neglected Tropical Diseases. Clin Infect Dis. 2020; doi: 10.1093/cid/ciaa1202.
72. Girard AE, Girard D, English AR, Gootz TD, Cimochowski CR, Faiella JA, et al. Pharmacokinetic and *in vivo* studies with azithromycin (CP-62,993), a new macrolide with an extended half-life and excellent tissue distribution. Antimicrob Agents Chemother. 1987;31 12:1948-54; doi: doi:10.1128/AAC.31.12.1948. https://journals.asm.org/doi/abs/10.1128/AAC.31.12.1948
73. Abdulai AA, Agana-Nsiire P, Biney F, Kwakye-Maclean C, Kyei-Faried S, Amponsa-Achiano K, et al. Community-based mass treatment with azithromycin for the elimination of yaws in Ghana—Results of a pilot study. PLOS Negl Trop Dis. 2018;12 3:e0006303; doi: 10.1371/journal.pntd.0006303. https://doi.org/10.1371/journal.pntd.0006303.
74. Mitjà O, González-Beiras C, Godornes C, Kolmau R, Houinei W, Abel H, et al. Effectiveness of single-dose azithromycin to treat latent yaws: a longitudinal comparative cohort study. Lancet Glob Health. 2017;5 12:e1268-e74; doi: 10.1016/s2214-109x(17)30388-1.
75. Asiedu K, Fitzpatrick C, Jannin J. Eradication of yaws: Historical efforts and achieving WHO's 2020 target. PLOS Negl Trop Dis. 2014;8 9:e3016; doi: 10.1371/journal.pntd.0003016. https://doi.org/10.1371/journal.pntd.0003016.
76. Maxfield L, Corley JE, Crane JS: Yaws. https://www.ncbi.nlm.nih.gov/books/NBK526013/ (2022). Accessed 7/19/2022 2022.
77. Solomon AW, Holland MJ, Alexander NDE, Massae PA, Aguirre A, Natividad-Sancho A, et al. Mass treatment with single-dose azithromycin for Trachoma. N Engl J Med. 2004;351 19:1962-71; doi: 10.1056/NEJMoa040979. https://www.nejm.org/doi/full/10.1056/NEJMoa040979.
78. Fraser-Hurt N, Bailey RL, Cousens S, Mabey D, Faal H, Mabey DC. Efficacy of oral azithromycin versus topical tetracycline in mass treatment of endemic trachoma. Bull World Health Organ. 2001;79 7:632-40. https://pubmed.ncbi.nlm.nih.gov/11477966.
79. Marks M, Mitjà O, Bottomley C, Kwakye C, Houinei W, Bauri M, et al. Comparative efficacy of low-dose versus standard-dose azithromycin for patients with yaws: a randomised non-inferiority trial in Ghana and Papua New Guinea. Lancet Glob

Health. 2018;6 4:e401-e10; doi: https://doi.org/10.1016/S2214-109X(18)30023-8. https://www.sciencedirect.com/science/article/pii/S2214109X18300238.

80. Fletcher AP, Knappett CR. N,N'-dibenzylethylenediamine penicillin; a new repository form of penicillin. Br Med J. 1953;1 4803:188-9; doi: 10.1136/bmj.1.4803.188.

81. Gartlan WA, Rahman S, Reti K. Benzathine penicillin. StatPearls. Treasure Island (FL): StatPearls Publishing, Copyright © 2022, StatPearls Publishing LLC.; 2022.

82. Djenontin A, Ahoua Alou LP, Koffi A, Zogo B, Duarte E, N'Guessan R, et al. Insecticidal and sterilizing effect of Olyset Duo(R), a permethrin and pyriproxyfen mixture net against pyrethroid-susceptible and -resistant strains of Anopheles gambiae s.s.: a release-recapture assay in experimental huts. Parasite. 2015;22:27; doi: 10.1051/parasite/2015027 [doi];parasite150026 [pii]. http://www.ncbi.nlm.nih.gov/pubmed/26489479.

83. Pennetier C, Bouraima A, Chandre F, Piameu M, Etang J, Rossignol M, et al. Efficacy of Olyset® plus, a new long-lasting insecticidal net incorporating permethrin and piperonil-butoxide against multi-resistant malaria vectors. PloS One. 2013;8 10:e75134; doi: 10.1371/journal.pone.0075134. https://go.exlibris.link/wjpy1c9F.

84. World Health Organization. Report of the thirteenth [13th] WHOPES working group meeting : WHO/HQ, Geneva, 28-30 July 2009 : review of Olyset LN, Dawaplus 2.0 LN, Tianjin Yorkool LN. 2009. https://apps.who.int/iris/handle/10665/44212.

85. World Health Organization. Report of the fourteenth [14th] WHOPES working group meeting : WHO/HQ, Geneva, 11-15 April2011 : review of Spinosad EC, Lifenet LN, Magnet LN, Royal Sentry LN, Yahe LN. 2011. https://apps.who.int/iris/handle/10665/44669.

86. World Health Organization. Report of the fifteenth WHOPES working group meeting: WHO/HQ, Geneva, 18-22 June 2012: review of Olyset plus, Interceptor LN, Malathion 440 EW, Vectobac GR. 2012. https://apps.who.int/iris/handle/10665/75304.

87. US FDA Center for Drug Evaluation and Research. Approval package for ANDA 074806 Permethrin Cream 5%. 1998. https://www.accessdata.fda.gov/drugsatfda_docs/anda/98/074806ap.pdf.

88. Currie BJ, McCarthy JS. Permethrin and ivermectin for scabies. N Engl J Med. 2010;362 8:717-25; doi: 362/8/717 [pii];10.1056/NEJMct0910329 [doi]. http://www.ncbi.nlm.nih.gov/pubmed/20181973.

89. Ogbuefi N, Kenner-Bell B. Common pediatric infestations: update on diagnosis and treatment of scabies, head lice, and bed bugs. Curr Opin Pediatr. 2021;33 4:410-5; doi: 10.1097/MOP.0000000000001031. https://www.ncbi.nlm.nih.gov/pubmed/34074914.

90. Romani L, Whitfeld MJ, Koroivueta J, Kama M, Wand H, Tikoduadua L, et al. Mass Drug Administration for Scabies - 2 Years of Follow-up. N Engl J Med. 2019;381 2:186-7; doi: 10.1056/NEJMc1808439 [doi]. http://www.ncbi.nlm.nih.gov/pubmed/31242358.

91. Engelman D, Cantey PT, Marks M, Solomon AW, Chang AY, Chosidow O, et al. The public health control of scabies: priorities for research and action. Lancet. 2019;394 10192:81-92; doi: S0140-6736(19)31136-5 [pii];10.1016/S0140-6736(19)31136-5 [doi]. http://www.ncbi.nlm.nih.gov/pubmed/31178154.

92. Engelman D, Steer AC. Control strategies for scabies. Trop Med Infect Dis. 2018;3 3:98; doi: 10.3390/tropicalmed3030098. https://pubmed.ncbi.nlm.nih.gov/30274494.
93. La Vincente S, Kearns T, Connors C, Cameron S, Carapetis J, Andrews R. Community management of endemic scabies in remote aboriginal communities of northern Australia: low treatment uptake and high ongoing acquisition. PLoS Negl Trop Dis. 2009;3 5:e444-e; doi: 10.1371/journal.pntd.0000444. https://go.exlibris.link/rP5SLXYR.
94. Thomas J, Peterson GM, Walton SF, Carson CF, Naunton M, Baby KE. Scabies: an ancient global disease with a need for new therapies. BMC Infect Dis. 2015;15 1:250-; doi: 10.1186/s12879-015-0983-z. https://bmcinfectdis.biomedcentral.com/articles/10.1186/s12879-015-0983-z.
95. Engelman D, Marks M, Steer AC, Beshah A, Biswas G, Chosidow O, et al. A framework for scabies control. PLoS Negl Trop Dis. 2021;15 9:e0009661; doi: 10.1371/journal.pntd.0009661.
96. World Health Organization. WHO informal consultation on a framework for scabies control: World Health Organization Regional Office for the Western Pacific: Manila, Philippines, 19–21 February 2019: meeting report. 2020. https://apps.who.int/iris/handle/10665/333154.
97. Karthikeyan K. Treatment of scabies: newer perspectives. Postgraduate Med J. 2005;81 951:7-11; doi: 10.1136/pgmj.2003.018390. https://pmj.bmj.com/content/postgradmedj/81/951/7.full.pdf.
98. Walton SF, Myerscough MR, Currie BJ. Studies *in vitro* on the relative efficacy of current acaricides for *Sarcoptes scabiei* var. hominis. Trans R Soc Trop Med Hyg. 2000;94 1:92-6. http://www.ncbi.nlm.nih.gov/pubmed/10748911.
99. King RE. The benzyl benzoate treatment of scabies. Br Med J. 1940;2 4166:626-7; doi: 10.1136/bmj.2.4166.626. https://www.ncbi.nlm.nih.gov/pmc/articles/PMC2179642/.
100. Bernigaud C, Fang F, Fischer K, Lespine A, Aho LS, Dreau D, et al. Preclinical study of single-dose moxidectin, a new oral treatment for scabies: Efficacy, safety, and pharmacokinetics compared to two-dose ivermectin in a porcine model. PLoS Negl Trop Dis. 2016;10 10:e0005030; doi: 10.1371/journal.pntd.0005030. https://www.ncbi.nlm.nih.gov/pubmed/27732588.
101. Chandler DJ, Fuller LC. A Review of Scabies: An Infestation More than Skin Deep. Dermatology. 2019;235 2:79-90; doi: 10.1159/000495290. https://www.karger.com/DOI/10.1159/000495290.
102. Bernigaud C, Fischer K, Chosidow O. The management of Scabies in the 21st century: Past, advances and potentials. Acta Derm Venereol. 2020;100 9:adv00112; doi: 10.2340/00015555-3468. https://www.ncbi.nlm.nih.gov/pubmed/32207535.
103. Buratti FM, D'Aniello A, Volpe MT, Meneguz A, Testai E. Malathion bioactivation in the human liver: the contribution of different cytochrome p450 isoforms. Drug Metab Dispos. 2005;33 3:295-302; doi: dmd.104.001693 [pii];10.1124/dmd.104.001693 [doi]. http://www.ncbi.nlm.nih.gov/pubmed/15557345.

104. Krstic DZ, Colovic M, Kralj MB, Franko M, Krinulovic K, Trebse P, et al. Inhibition of AChE by malathion and some structurally similar compounds. J Enzyme Inhib Med Chem. 2008;23 4:562-73; doi: 791964418 [pii];10.1080/14756360701632031 [doi]. http://www.ncbi.nlm.nih.gov/pubmed/18608787.
105. WHO International Agency for Research on Cancer: Some organophosphate insecticides and herbicides. vol. 1122017.
106. Hanna NF, Clay JC, Harris JR. *Sarcoptes scabiei* infestation treated with malathion liquid. Br J Vener Dis. 1978;54 5:354; doi: 10.1136/sti.54.5.354.
107. Johnstone P, Strong M. Scabies. BMJ Clin Evid. 2014;2014; doi: 1707 [pii]. http://www.ncbi.nlm.nih.gov/pubmed/25544114.
108. Salavastru CM, Chosidow O, Boffa MJ, Janier M, Tiplica GS. European guideline for the management of scabies. J Eur Acad Dermatol. 2017;31 8:1248-53; doi: 10.1111/jdv.14351. https://onlinelibrary.wiley.com/doi/10.1111/jdv.14351.
109. WHO International Agency for Research on Cancer: IARC News: IARC Monographs Volume 112: evaluation of five organophosphate insecticides and herbicides. https://www.iarc.who.int/news-events/iarc-monographs-volume-112-evaluation-of-five-organophosphate-insecticides-and-herbicides/ (2015).
110. Enbiale W, Baynie TB, Ayalew A, Gebrehiwot T, Getanew T, Ayal A, et al. "Stopping the itch": mass drug administration for scabies outbreak control covered for over nine million people in Ethiopia. J Infect Dev Ctries. 2020;14 6.1:28S-35S; doi: 10.3855/jidc.11701 [doi]. http://www.ncbi.nlm.nih.gov/pubmed/32614793.
111. Pruksachatkunakorn C, Damrongsak M, Sinthupuan S. Sulfur for scabies outbreaks in orphanages. Pediatr Dermatol. 2002;19 5:448-53; doi: 10.1046/j.1525-1470.2002.00205.x. https://onlinelibrary.wiley.com/doi/full/10.1046/j.1525-1470.2002.00205.x.
112. Grobbelaar M, Louw GE, Sampson SL, van Helden PD, Donald PR, Warren RM. Evolution of rifampicin treatment for tuberculosis. Infect Genet Evol. 2019;74:103937; doi: S1567-1348(19)30158-3 [pii];10.1016/j.meegid.2019.103937 [doi]. http://www.ncbi.nlm.nih.gov/pubmed/31247337.
113. Mosaei H, Zenkin N. Inhibition of RNA polymerase by rifampicin and rifamycin-like molecules. EcoSal Plus. 2020;9 1; doi: 10.1128/ecosalplus.ESP-0017-2019 [doi]. http://www.ncbi.nlm.nih.gov/pubmed/32342856.
114. Maartens G, Boffito M, Flexner CW. Compatibility of next-generation first-line antiretrovirals with rifampicin-based antituberculosis therapy in resource limited settings. Curr Opin HIV AIDS. 2017;12 4:355-8; doi: 10.1097/COH.0000000000000376 [doi]. http://www.ncbi.nlm.nih.gov/pubmed/28403028.
115. Ferreira SMB, Yonekura T, Ignotti E, Oliveira LB, Takahashi J, Soares CB. Effectiveness of rifampicin chemoprophylaxis in preventing leprosy in patient contacts: a systematic review of quantitative and qualitative evidence. JBI Database System Rev Implement Rep. 2017;15 10:2555-84; doi: 10.11124/JBISRIR-2016-003301 [doi];01938924-201710000-00018 [pii]. http://www.ncbi.nlm.nih.gov/pubmed/29035966.
116. Molinelli E, Paolinelli M, Campanati A, Brisigotti V, Offidani A. Metabolic, pharmacokinetic, and toxicological issues surrounding dapsone. Expert Opin Drug

Metab Toxicol. 2019;15 5:367-79; doi: 10.1080/17425255.2019.1600670 [doi]. http://www.ncbi.nlm.nih.gov/pubmed/30943794.

117. Sataporngpong P, Pratoomwun J, Rerknimitr P, Klaewsongkram J, Nakkam N, Rungrotmongkol T, et al. HLA-B*13 :01 is a predictive marker of dapsone-induced severe cutaneous adverse reactions in Thai patients. Front Immunol. 2021;12:661135; doi: 10.3389/fimmu.2021.661135 [doi]. http://www.ncbi.nlm.nih.gov/pubmed/34017337.

118. Tangamornsuksan W, Lohitnavy M. Association Between HLA-B*1301 and Dapsone-Induced Cutaneous Adverse Drug Reactions: A Systematic Review and Meta-analysis. JAMA Dermatol. 2018;154 4:441-6; doi: 2674263 [pii];10.1001/jamadermatol.2017.6484 [doi]. http://www.ncbi.nlm.nih.gov/pubmed/29541744.

119. Zhang FR, Liu H, Irwanto A, Fu XA, Li Y, Yu GQ, et al. HLA-B*13:01 and the dapsone hypersensitivity syndrome. N Engl J Med. 2013;369 17:1620-8; doi: 10.1056/NEJMoa1213096 [doi]. http://www.ncbi.nlm.nih.gov/pubmed/24152261.

120. Pearson JM, Cap JA, Haile GS, Rees RJ. Dapsone-resistant leprosy and its implications for leprosy control programmes. Lepr Rev. 1977;48 2:83-94; doi: 10.5935/0305-7518.19770010 [doi]. http://www.ncbi.nlm.nih.gov/pubmed/331000.

121. Pearson JM, Haile GS, Rees RJ. Primary dapsone-resistant leprosy. Lepr Rev. 1977;48 2:129-32; doi: 10.5935/0305-7518.19770016 [doi]. http://www.ncbi.nlm.nih.gov/pubmed/330999.

122. Cambau E, Carthagena L, Chauffour A, Ji B, Jarlier V. Dihydropteroate synthase mutations in the folP1 gene predict dapsone resistance in relapsed cases of leprosy. Clin Infect Dis. 2006;42 2:238-41; doi: CID36286 [pii];10.1086/498506 [doi]. http://www.ncbi.nlm.nih.gov/pubmed/16355335.

123. Cholo MC, Mothiba MT, Fourie B, Anderson R. Mechanisms of action and therapeutic efficacies of the lipophilic antimycobacterial agents clofazimine and bedaquiline. J Antimicrob Chemother. 2017;72 2:338-53; doi: dkw426 [pii];10.1093/jac/dkw426 [doi]. http://www.ncbi.nlm.nih.gov/pubmed/27798208.

124. Cholo MC, Steel HC, Fourie PB, Germishuizen WA, Anderson R. Clofazimine: current status and future prospects. J Antimicrob Chemother. 2012;67 2:290-8; doi: dkr444 [pii];10.1093/jac/dkr444 [doi]. http://www.ncbi.nlm.nih.gov/pubmed/22020137.

125. Zhang S, Chen J, Cui P, Shi W, Zhang W, Zhang Y. Identification of novel mutations associated with clofazimine resistance in *Mycobacterium tuberculosis*. J Antimicrob Chemother. 2015;70 9:2507-10; doi: dkv150 [pii];10.1093/jac/dkv150 [doi]. http://www.ncbi.nlm.nih.gov/pubmed/26045528.

126. Sturgill MG, Rapp RP. Clarithromycin: review of a new macrolide antibiotic with improved microbiologic spectrum and favorable pharmacokinetic and adverse effect profiles. Ann Pharmacother. 1992;26 9:1099-108; doi: 10.1177/106002809202600912 [doi]. http://www.ncbi.nlm.nih.gov/pubmed/1421677.

127. Zimmermann P, Ziesenitz VC, Curtis N, Ritz N. The Immunomodulatory Effects of Macrolides-A Systematic Review of the Underlying Mechanisms. Front Immunol. 2018;9:302; doi: 10.3389/fimmu.2018.00302 [doi]. http://www.ncbi.nlm.nih.gov/pubmed/29593707.

128. Pai MP, Graci DM, Amsden GW. Macrolide drug interactions: an update. Ann Pharmacother. 2000;34 4:495-513; doi: 10.1345/aph.19138 [doi]. http://www.ncbi.nlm.nih.gov/pubmed/10772438.
129. Westphal JF. Macrolide - induced clinically relevant drug interactions with cytochrome P-450A (CYP) 3A4: an update focused on clarithromycin, azithromycin and dirithromycin. Br J Clin Pharmacol. 2000;50 4:285-95; doi: bcp261 [pii];10.1046/j.1365-2125.2000.00261.x [doi]. http://www.ncbi.nlm.nih.gov/pubmed/11012550.
130. Blondeau JM, Hansen GT. Moxifloxacin: a review of the microbiological, pharmacological, clinical and safety features. Expert Opin Pharmacother. 2001;2 2:317-35; doi: 10.1517/14656566.2.2.317 [doi]. http://www.ncbi.nlm.nih.gov/pubmed/11336589.
131. Chopra I, Roberts M. Tetracycline antibiotics: mode of action, applications, molecular biology, and epidemiology of bacterial resistance. Microbiol Mol Biol Rev. 2001;65 2:232-60 ; second page, table of contents; doi: 10.1128/MMBR.65.2.232-260.2001. https://www.ncbi.nlm.nih.gov/pubmed/11381101.
132. Kaiser M, Bray MA, Cal M, Bourdin TB, Torreele E, Brun R. Antitrypanosomal activity of fexinidazole, a new oral nitroimidazole drug candidate for treatment of sleeping sickness. Antimicrob Agents Chemother. 2011;55 12:5602-8; doi: AAC.00246-11 [pii];10.1128/AAC.00246-11 [doi]. http://www.ncbi.nlm.nih.gov/pubmed/21911566.
133. Wyllie S, Foth BJ, Kelner A, Sokolova AY, Berriman M, Fairlamb AH. Nitroheterocyclic drug resistance mechanisms in *Trypanosoma brucei*. J Antimicrob Chemother. 2016;71 3:625-34; doi: dkv376 [pii];10.1093/jac/dkv376 [doi]. http://www.ncbi.nlm.nih.gov/pubmed/26581221.
134. Torreele E, Bourdin TB, Tweats D, Kaiser M, Brun R, Mazue G, et al. Fexinidazole--a new oral nitroimidazole drug candidate entering clinical development for the treatment of sleeping sickness. PLoS Negl Trop Dis. 2010;4 12:e923; doi: 10.1371/journal.pntd.0000923 [doi]. http://www.ncbi.nlm.nih.gov/pubmed/21200426.
135. Dickie EA, Giordani F, Gould MK, Maser P, Burri C, Mottram JC, et al. New drugs for Human African Trypanosomiasis: A twenty first century success story. Trop Med Infect Dis. 2020;5 1; doi: tropicalmed5010029 [pii];10.3390/tropicalmed5010029 [doi]. http://www.ncbi.nlm.nih.gov/pubmed/32092897.
136. Committee for Medicinal Products for Human Use (CHMP). CHMP Summary of opinion for Fexinidazole Winthrop. 2018. https://www.ema.europa.eu/en/opinion-medicine-use-outside-EU/human/fexinidazole-winthrop.
137. Valverde MO, Tarral A, Strub-Wourgaft N. Development and Introduction of Fexinidazole into the Global Human African Trypanosomiasis Program. Am J Trop Med Hyg. 2022;106 (5 Suppl):61-6; doi: 10.4269/ajtmh.21-1176. https://www.ncbi.nlm.nih.gov/pmc/articles/PMC9154641/.
138. World Health Organization. WHO interim guidelines for the treatment of gambiense human African trypanosomiasis. 2019. https://apps.who.int/iris/handle/10665/326178.
139. Ferreira LLG, Andricopulo AD. Drugs and vaccines in the 21st century for neglected diseases. Lancet Infect Dis. 2019;19 2:125-7; doi: S1473-3099(19)30005-2

[pii];10.1016/S1473-3099(19)30005-2 [doi]. http://www.ncbi.nlm.nih.gov/pubmed/30712832.

140. World Health Organization. Control and surveillance of human African trypanosomiasis: report of a WHO expert committee. vol. 984. Italy: World Health Organization; 2013.

141. Thomas JA, Baker N, Hutchinson S, Dominicus C, Trenaman A, Glover L, et al. Insights into antitrypanosomal drug mode-of-action from cytology-based profiling. PLoS Negl Trop Dis. 2018;12 11:e0006980; doi: 10.1371/journal.pntd.0006980.

142. Worthen C, Jensen BC, Parsons M. Diverse effects on mitochondrial and nuclear functions elicited by drugs and genetic knockdowns in bloodstream stage *Trypanosoma brucei*. PLOS Neglected Tropical Diseases. 2010;4 5:e678; doi: 10.1371/journal.pntd.0000678. https://doi.org/10.1371/journal.pntd.0000678.

143. Lutje V, Seixas J, Kennedy A. Chemotherapy for second-stage human African trypanosomiasis. Cochrane Database Syst Rev. 2013;2013 6:Cd006201; doi: 10.1002/14651858.CD006201.pub3.

144. Barrett MP, Croft SL. Management of trypanosomiasis and leishmaniasis. Br Med Bull. 2012;104 1:175-96; doi: 10.1093/bmb/lds031.

145. Wiedemar N, Hauser DA, Mäser P. 100 years of suramin. Antimicrob Agents Chemother. 2020;64 3:e01168-19; doi: doi:10.1128/AAC.01168-19. https://journals.asm.org/doi/abs/10.1128/AAC.01168-19.

146. World Health Organization. WHO Expert Committee on Onchocerciasis, 3rd Report. Technical Report Series 752. 1987;752.

147. World Health Organization. Onchocerciasis and its control. Report of a WHO Expert Committee on Onchocerciasis Control. Technical Report Series 852. 1995;852. https://apps.who.int/iris/handle/10665/37346.

148. Kuepfer I, Schmid C, Allan M, Edielu A, Haary EP, Kakembo A, et al. Safety and efficacy of the 10-Day melarsoprol schedule for the treatment of second stage rhodesiense sleeping sickness. PLOS Negl Trop Dis. 2012;6 8:e1695; doi: 10.1371/journal.pntd.0001695. https://doi.org/10.1371/journal.pntd.0001695.

149. Burri C, Baltz T, Giroud C, Doua F, Welker HA, Brun R. Pharmacokinetic properties of the trypanocidal drug melarsoprol. Chemotherapy. 1993;39 4:225-34; doi: 10.1159/000239130 [doi]. http://www.ncbi.nlm.nih.gov/pubmed/8391966.

150. Burri C, Onyango JD, Auma JE, Burudi EM, Brun R. Pharmacokinetics of melarsoprol in uninfected vervet monkeys. Acta Trop. 1994;58 1:35-49; doi: 0001-706X(94)90120-1 [pii];10.1016/0001-706x(94)90120-1 [doi]. http://www.ncbi.nlm.nih.gov/pubmed/7863853.

151. Schmid C, Richer M, Bilenge CM, Josenando T, Chappuis F, Manthelot CR, et al. Effectiveness of a 10-day melarsoprol schedule for the treatment of late-stage human African trypanosomiasis: confirmation from a multinational study (IMPAMEL II). J Infect Dis. 2005;191 11:1922-31; doi: JID33782 [pii];10.1086/429929 [doi]. http://www.ncbi.nlm.nih.gov/pubmed/15871127.

152. UNDP/World Bank/WHO Special Programme for Research and Training in Tropical Diseases. Tropical disease research : progress 1999-2000 : fifteenth programme

report / UNDP/World Bank/WHO Special Programme for Research and Training in Tropical Diseases. 2001. https://apps.who.int/iris/handle/10665/66942.
153. Doua F, Boa FY, Schechter PJ, Miezan TW, Diai D, Sanon SR, et al. Treatment of human late stage gambiense trypanosomiasis with alpha-difluoromethylornithine (eflornithine): efficacy and tolerance in 14 cases in Cote d'Ivoire. Am J Trop Med Hyg. 1987;37 3:525-33; doi: 10.4269/ajtmh.1987.37.525 [doi]. http://www.ncbi.nlm.nih.gov/pubmed/3120607.
154. UNDP/World Bank/WHO Special Programme for Research and Training in Tropical Diseases. Tropical disease research : progress 1975-94, highlights 1993-94 , twelfth programme report of the UNDP/World Bank/WHO Special Programme for Research and Training in Tropical Diseases (TDR). 1995. https://apps.who.int/iris/handle/10665/36921.
155. World Health Organization. Human African Trypanosomiasis Treatment and Drug Resistance Network: Report of the first meeting, Geneva, Switzerland, 14-15 April 1999. 1999. https://apps.who.int/iris/handle/10665/66003.
156. US FDA Center for Drug Evaluation and Research. Letter and reviews relating to Vaniqua (eflornithin hydrochlorine) cream to Bristol-Myers Squibb. 2000. https://www.accessdata.fda.gov/drugsatfda_docs/appletter/2000/21145ltr.pdf
https://www.accessdata.fda.gov/drugsatfda_docs/nda/2000/21145_Vaniqa.cfm.
157. Ebikeme C. The death and life of the resurrection drug. PLOS Negl Trop Dis. 2014;8 7:e2910; doi: 10.1371/journal.pntd.0002910 [doi];PNTD-D-13-01790 [pii]. http://www.ncbi.nlm.nih.gov/pubmed/25010692.
158. Simarro PP, Franco J, Diarra A, Postigo JA, Jannin J. Update on field use of the available drugs for the chemotherapy of human African trypanosomiasis. Parasitology. 2012;139 7:842-6; doi: S0031182012000169 [pii];10.1017/S0031182012000169 [doi]. http://www.ncbi.nlm.nih.gov/pubmed/22309684.
159. World Health Organization: Neglected tropical diseases: WHO and Sanofi renew decades-long collaboration to sustain elimination efforts. https://www.who.int/news/item/15-12-2020-neglected-tropical-diseases-who-and-sanofi-renew-decades-long-collaboration-to-sustain-elimination-efforts (2020). Accessed 6/20/2022 2022.
160. Priotto G, Kasparian S, Mutombo W, Ngouama D, Ghorashian S, Arnold U, et al. Nifurtimox-eflornithine combination therapy for second-stage African *Trypanosoma brucei gambiense* trypanosomiasis: a multicentre, randomised, phase III, non-inferiority trial. Lancet. 2009;374 9683:56-64; doi: S0140-6736(09)61117-X [pii];10.1016/S0140-6736(09)61117-X [doi]. http://www.ncbi.nlm.nih.gov/pubmed/19559476.
161. Kansiime F, Adibaku S, Wamboga C, Idi F, Kato CD, Yamuah L, et al. A multicentre, randomised, non-inferiority clinical trial comparing a nifurtimox-eflornithine combination to standard eflornithine monotherapy for late stage *Trypanosoma brucei gambiense* human African trypanosomiasis in Uganda. Parasit Vectors. 2018;11 1:105; doi: 10.1186/s13071-018-2634-x. https://doi.org/10.1186/s13071-018-2634-x.

162. Caldas IS, Santos EG, Novaes RD. An evaluation of benznidazole as a Chagas disease therapeutic. Expert Opin Pharmacother. 2019;20 15:1797-807; doi: 10.1080/14656566.2019.1650915 [doi]. http://www.ncbi.nlm.nih.gov/pubmed/31456439.

163. Rajao MA, Furtado C, Alves CL, Passos-Silva DG, de Moura MB, Schamber-Reis BL, et al. Unveiling benznidazole's mechanism of action through overexpression of DNA repair proteins in *Trypanosoma cruzi*. Environ Mol Mutagen. 2014;55 4:309-21; doi: 10.1002/em.21839 [doi]. http://www.ncbi.nlm.nih.gov/pubmed/24347026.

164. Trochine A, Creek DJ, Faral-Tello P, Barrett MP, Robello C. Benznidazole biotransformation and multiple targets in *Trypanosoma cruzi* revealed by metabolomics. PLoS Negl Trop Dis. 2014;8 5:e2844; doi: 10.1371/journal.pntd.0002844 [doi];PNTD-D-13-02034 [pii]. http://www.ncbi.nlm.nih.gov/pubmed/24853684.

165. Galvan IL, Alonso-Padilla J, Cortes-Serra N, Alonso-Vega C, Gascon J, Pinazo MJ. Benznidazole for the treatment of Chagas disease. Expert Rev Anti-infect Ther. 2021;19 5:547-56; doi: 10.1080/14787210.2021.1834849.

166. Vallejo M, Reyes PP, Martinez GM, Gonzalez Garay AG. Trypanocidal drugs for late-stage, symptomatic Chagas disease (Trypanosoma cruzi infection). Cochrane Database Syst Rev. 2020;12:CD004102; doi: 10.1002/14651858.CD004102.pub3 [doi]. http://www.ncbi.nlm.nih.gov/pubmed/33305846.

167. Altcheh J, Moscatelli G, Moroni S, Garcia-Bournissen F, Freilij H. Adverse events after the use of benznidazole in infants and children with Chagas disease. Pediatrics. 2011;127 1:e212-8; doi: 10.1542/peds.2010-1172. https://www.ncbi.nlm.nih.gov/pubmed/21173000.

168. Drugs for Neglected Diseases *initiative*: Chagas disease: Paediatric benznidazole. https://dndi.org/research-development/portfolio/paediatric-benznidazole/ (2021). Accessed 1/08/2022 2022.

169. Torrico F, Gascon J, Barreira F, Blum B, Almeida IC, Alonso-Vega C, et al. New regimens of benznidazole monotherapy and in combination with fosravuconazole for treatment of Chagas disease (BENDITA): a phase 2, double-blind, randomised trial. Lancet Infect Dis. 2021;21 8:1129-40; doi: 10.1016/S1473-3099(20)30844-6. https://www.ncbi.nlm.nih.gov/pubmed/33836161.

170. Malone CJ, Nevis I, Fernandez E, Sanchez A. A rapid review on the efficacy and safety of pharmacological treatments for Chagas disease. Trop Med Infect Dis. 2021;6 3; doi: 10.3390/tropicalmed6030128. https://www.ncbi.nlm.nih.gov/pubmed/34287382.

171. Stass H, Just S, Weimann B, Ince I, Willmann S, Feleder E, et al. Clinical investigation of the biopharmaceutical characteristics of nifurtimox tablets - Implications for quality control and application. Eur J Pharm Sci. 2021;166:105940; doi: S0928-0987(21)00243-8 [pii];10.1016/j.ejps.2021.105940 [doi]. http://www.ncbi.nlm.nih.gov/pubmed/34265407.

172. World Health Organization. The selection and use of essential medicines : report of the WHO Expert Committee, March 2009 (including the 16th WHO model list of essential medicines and the 2nd WHO model list of essential medicines for children). 2009. https://apps.who.int/iris/handle/10665/44287.

173. Franco JR, Simarro PP, Diarra A, Ruiz-Postigo JA, Samo M, Jannin JG. Monitoring the use of nifurtimox-eflornithine combination therapy (NECT) in the treatment of second stage gambiense human African trypanosomiasis. Res Rep Trop Med. 2012;3:93-101; doi: 10.2147/RRTM.S34399 [doi];rrtm-3-093 [pii]. http://www.ncbi.nlm.nih.gov/pubmed/30100776.
174. World Health Organization. WHO model list of essential medicines for children : 4th list, April 2013. 2013. https://apps.who.int/iris/handle/10665/93143.
175. Alirol E, Schrumpf D, Amici HJ, Riedel A, de PC, QUERE M, et al. Nifurtimox-eflornithine combination therapy for second-stage gambiense human African trypanosomiasis: Medecins Sans Frontieres experience in the Democratic Republic of the Congo. Clin Infect Dis. 2013;56 2:195-203; doi: cis886 [pii];10.1093/cid/cis886 [doi]. http://www.ncbi.nlm.nih.gov/pubmed/23074318.
176. Schmid C, Kuemmerle A, Blum J, Ghabri S, Kande V, Mutombo W, et al. In-hospital safety in field conditions of nifurtimox eflornithine combination therapy (NECT) for T. b. gambiense sleeping sickness. PLoS Negl Trop Dis. 2012;6 11:e1920; doi: 10.1371/journal.pntd.0001920 [doi];PNTD-D-12-00537 [pii]. http://www.ncbi.nlm.nih.gov/pubmed/23209861.
177. World Health Organization. The selection and use of essential medicines: report of the WHO Expert Committee, 2013 (including the 18th WHO model list of essential medicines and the 4th WHO model list of essential medicines for children). 2014. https://apps.who.int/iris/handle/10665/112729.
178. WHO Expert Committee on the Control of the Leishmaniases & World Health Organization. Control of the leishmaniases: Report of a meeting of the WHO Expert Commitee on the Control of Leishmaniases, Geneva, 22-26 March 2010. WHO technical report series. 2010. https://apps.who.int/iris/handle/10665/44412.
179. Frézard F, Demicheli C, Ribeiro RR. Pentavalent antimonials: new perspectives for old drugs. Molecules (Basel, Switzerland). 2009;14 7:2317-36; doi: 10.3390/molecules14072317. https://go.exlibris.link/CBSNsqzf.
180. Kip AE, Schellens JHM, Beijnen JH, Dorlo TPC. Clinical pharmacokinetics of systemically administered antileishmanial drugs. Clin Pharamcokinet. 2018;57 2:151-76; doi: 10.1007/s40262-017-0570-0. https://pubmed.ncbi.nlm.nih.gov/28756612/.
181. World Health Organization. WHO guideline for the treatment of visceral leishmaniasis in HIV co-infected patients in East Africa and South-East Asia. 2022. https://apps.who.int/iris/handle/10665/354703.
182. World Health Organization. WHO guideline for the treatment of visceral leishmaniasis in HIV co-infected patients in East Africa and South-East Asia: web annex B: evidence-to-decision tables. 2022. https://apps.who.int/iris/handle/10665/354547.
183. World Health Organization & Cochrane Response. WHO guideline for the treatment of visceral leishmaniasis in HIV co-infected patients in East Africa and South-East Asia: web annex A: a systematic review on the treatment of visceral leishmaniasis in HIV-Leishmania co-infected persons in East Africa and South-East Asia. 2022. https://apps.who.int/iris/handle/10665/354546.
184. Mesa-Arango AC, Scorzoni L, Zaragoza O. It only takes one to do many jobs: Amphotericin B as antifungal and immunomodulatory drug. Front Microbiol.

2012;3:286; doi: 10.3389/fmicb.2012.00286 [doi]. http://www.ncbi.nlm.nih.gov/pubmed/23024638.
185. Bern C, Adler-Moore J, Berenguer J, Boelaert M, den BM, Davidson RN, et al. Liposomal amphotericin B for the treatment of visceral leishmaniasis. Clin Infect Dis. 2006;43 7:917-24; doi: CID38167 [pii];10.1086/507530 [doi]. http://www.ncbi.nlm.nih.gov/pubmed/16941377.
186. Berman JD, Badaro R, Thakur CP, Wasunna KM, Behbehani K, Davidson R, et al. Efficacy and safety of liposomal amphotericin B (AmBisome) for visceral leishmaniasis in endemic developing countries. Bull World Health Organ. 1998;76 1:25-32. https://www.ncbi.nlm.nih.gov/pubmed/9615494.
187. Davidson RN, Croft SL, Scott A, Maini M, Moody AH, Bryceson AD. Liposomal amphotericin B in drug-resistant visceral leishmaniasis. Lancet. 1991;337 8749:1061-2; doi: 10.1016/0140-6736(91)91708-3.
188. Mondal D, Alvar J, Hasnain MG, Hossain MS, Ghosh D, Huda MM, et al. Efficacy and safety of single-dose liposomal amphotericin B for visceral leishmaniasis in a rural public hospital in Bangladesh: a feasibility study. Lancet Glob Health. 2014;2 1:e51-e7; doi: S2214-109X(13)70118-9 [pii];10.1016/S2214-109X(13)70118-9 [doi]. http://www.ncbi.nlm.nih.gov/pubmed/25104636.
189. Sundar S, Chakravarty J, Agarwal D, Rai M, Murray HW. Single-dose liposomal amphotericin B for visceral leishmaniasis in India. N Engl J Med. 2010;362 6:504-12; doi: 362/6/504 [pii];10.1056/NEJMoa0903627 [doi]. http://www.ncbi.nlm.nih.gov/pubmed/20147716.
190. Sundar S, Singh A, Agrawal N, Chakravarty J. Effectiveness of single-dose liposomal amphotericin B in visceralLeishmaniasis in Bihar. Am J Trop Med Hyg. 2019;101 4:795-8; doi: 10.4269/ajtmh.19-0179 [doi]. http://www.ncbi.nlm.nih.gov/pubmed/31436156.
191. Hirve S, Kroeger A, Matlashewski G, Mondal D, Banjara MR, Das P, et al. Towards elimination of visceral leishmaniasis in the Indian subcontinent-Translating research to practice to public health. PLoS Negl Trop Dis. 2017;11 10:e0005889; doi: 10.1371/journal.pntd.0005889 [doi];PNTD-D-17-01049 [pii]. http://www.ncbi.nlm.nih.gov/pubmed/29023446.
192. Mosimann V, Neumayr A, Paris DH, Blum J. Liposomal amphotericin B treatment of Old World cutaneous and mucosal leishmaniasis: A literature review. Acta Trop. 2018;182:246-50; doi: S0001-706X(17)31351-7 [pii];10.1016/j.actatropica.2018.03.016 [doi]. http://www.ncbi.nlm.nih.gov/pubmed/29550282.
193. Palic S, Beijnen JH, Dorlo TPC. An update on the clinical pharmacology of miltefosine in the treatment of leishmaniasis. Int J Antimicrob Agents. 2022;59 1:106459; doi: S0924-8579(21)01292-9 [pii];10.1016/j.ijantimicag.2021.106459 [doi]. http://www.ncbi.nlm.nih.gov/pubmed/34695563.
194. Sindermann H, Engel J. Development of miltefosine as an oral treatment for leishmaniasis. Trans R Soc Trop Med Hyg. 2006;100 Suppl 1:S17-S20; doi: S0035-9203(06)00109-X [pii];10.1016/j.trstmh.2006.02.010 [doi]. http://www.ncbi.nlm.nih.gov/pubmed/16730362.

195. Sunyoto T, Potet J, Boelaert M. Why miltefosine-a life-saving drug for leishmaniasis-is unavailable to people who need it the most. BMJ Glob Health. 2018;3 3:e000709-e; doi: 10.1136/bmjgh-2018-000709. https://pubmed.ncbi.nlm.nih.gov/29736277.
196. Croft SL, Engel J. Miltefosine--discovery of the antileishmanial activity of phospholipid derivatives. Trans R Soc Trop Med Hyg. 2006;100 Suppl 1:S4-S8; doi: S0035-9203(06)00197-0 [pii];10.1016/j.trstmh.2006.03.009 [doi]. http://www.ncbi.nlm.nih.gov/pubmed/16904717.
197. Sundar S, Jha TK, Thakur CP, Engel J, Sindermann H, Fischer C, et al. Oral miltefosine for Indian visceral leishmaniasis. N Engl J Med. 2002;347 22:1739-46; doi: 10.1056/NEJMoa021556 [doi];347/22/1739 [pii]. http://www.ncbi.nlm.nih.gov/pubmed/12456849.
198. Scientific Working Group on Leishmaniasis UNDP/World Bank/WHO Special Programme for Research and Training in Tropical Diseases. Report of the Scientific Working Group meeting on Leishmaniasis, Geneva, 2-4 February, 2004. 2004. https://apps.who.int/iris/handle/10665/68897.
199. World Health Organization Regional Office for South-East Asia. Regional strategic framework for elimination of kala azar from the South-East Asia Region (2005-2015). 2005; SEA-VBC-85. https://apps.who.int/iris/handle/10665/205825.
200. World Health Organization Regional Office for South-East Asia. Regional strategic framework for elimination of kala-azar from the South-East Asia Region (2011-2015). 2012. https://apps.who.int/iris/handle/10665/205826.
201. Gutteridge WE. TDR collaboration with the pharmaceutical industry. Trans R Soc Trop Med Hyg. 2006;100 Suppl 1:S21-S5; doi: S0035-9203(06)00112-X [pii];10.1016/j.trstmh.2006.02.013 [doi]. http://www.ncbi.nlm.nih.gov/pubmed/16730039.
202. Doshi P. US incentive scheme for neglected diseases: a good idea gone wrong? BMJ. 2014;349:g4665; doi: 10.1136/bmj.g4665 [doi]. http://www.ncbi.nlm.nih.gov/pubmed/25099712.
203. Ridley DB. Priorities for the priority review voucher. Am J Trop Med Hyg. 2017;96 1:14-5; doi: 10.4269/ajtmh.16-0600 [doi]. http://www.ncbi.nlm.nih.gov/pubmed/27573624.
204. Asilian A, Jalayer T, Nilforooshzadeh M, Ghassemi RL, Peto R, Wayling S, et al. Treatment of cutaneous leishmaniasis with aminosidine (paromomycin) ointment: double-blind, randomized trial in the Islamic Republic of Iran. Bull World Health Organ. 2003;81 5:353-9. https://www.ncbi.nlm.nih.gov/pubmed/12856053.
205. Asilian A, Jalayer T, Whitworth JA, Ghasemi RL, Nilforooshzadeh M, Olliaro P. A randomized, placebo-controlled trial of a two-week regimen of aminosidine (paromomycin) ointment for treatment of cutaneous leishmaniasis in Iran. Am J Trop Med Hyg. 1995;53 6:648-51; doi: 10.4269/ajtmh.1995.53.648. https://www.ncbi.nlm.nih.gov/pubmed/8561269.
206. Ben Salah A, Zakraoui H, Zaatour A, Ftaiti A, Zaafouri B, Garraoui A, et al. A randomized, placebo-controlled trial in Tunisia treating cutaneous leishmaniasis with paromomycin ointment. Am J Trop Med Hyg. 1995;53 2:162-6; doi: 10.4269/ajtmh.1995.53.162. https://www.ncbi.nlm.nih.gov/pubmed/7677218.

207. UNDP/World Bank/WHO Special Programme for Research Training in Tropical D. Tropical disease research : progress 1995-96 : thirteenth programme report / UNDP/WORLD BANK/WHO Special Programme for Research and Training in Tropical Diseases. 1997. https://apps.who.int/iris/handle/10665/42037.
208. UNDP/World Bank/WHO Special Programme for Research Training in Tropical D. Tropical disease research : progress 1997-98 : fourteenth programme report / UNDP/WORLD BANK/WHO Special Programme for Research and Training in Tropical Diseases. 1999; TDR/PR14/99.1. https://apps.who.int/iris/handle/10665/65970.
209. UNDP/World Bank/WHO Special Programme for Research Training in Tropical Diseases. Tropical disease research : progress 1999-2000 : fifteenth programme report / UNDP/World Bank/WHO Special Programme for Research and Training in Tropical Diseases. 2001; TDR/GEN/01.15. https://apps.who.int/iris/handle/10665/66942.
210. UNDP/World Bank/WHO Special Programme for Research Training in Tropical D. Progress 2001-2002 : sixteenth programme report : tropical disease research / UNDP/World Bank/WHO Special Programme for Research and Training in Tropical Diseases. 2003; TDR/GEN/03.1. https://apps.who.int/iris/handle/10665/68380.
211. Nunes M, Henriques AM, Bartosch C, Ricardo S. Recycling the purpose of old drugs to treat ovarian cancer. Int J Mol Sci. 2020;21 20; doi: ijms21207768 [pii];10.3390/ijms21207768 [doi]. http://www.ncbi.nlm.nih.gov/pubmed/33092251.
212. Pierard GE, Arrese JE, Pierard-Franchimont C. Itraconazole. Expert Opin Pharmacother. 2000;1 2:287-304; doi: 10.1517/14656566.1.2.287 [doi]. http://www.ncbi.nlm.nih.gov/pubmed/11249550.
